# Degradable Vinyl-Based Polymers by Radical Ring-Opening Polymerization: A User Guide

**DOI:** 10.1021/acspolymersau.5c00191

**Published:** 2026-04-11

**Authors:** Bastien Luzel, Sophia Kouider, Franck D’Agosto, Didier Gigmes, Muriel Lansalot, Christopher M. Bates, Elise Ackerman, Steven Labalme, Jeremiah A. Johnson, Jia Niu, Julien Nicolas, Yohann Guillaneuf, Catherine Lefay

**Affiliations:** † 128791 Aix-Marseille Université, CNRS, Institut de Chimie Radicalaire, UMR 7273, F-13397 Marseille, France; ‡ Universite Claude Bernard Lyon 1, CPE Lyon, CNRS, UMR 5128, Catalysis, Polymerization, Processes and Materials, 69616 Villeurbanne, France; § Materials Research Laboratory, 8786 University of California, Santa Barbara, California 93106, United States; ∇ Materials Department, University of California, Santa Barbara, California 93106, United States; ∥ Department of Chemistry, 2167 Massachusetts Institute of Technology, Cambridge, Massachusetts 02139, United States; ⊥ Department of Chemistry, 6019 Boston College, Chestnut Hill, Massachusetts 02467, United States; # Université Paris-Saclay, CNRS, 27048 Institut Galien Paris-Saclay, F-91400 Orsay, France

**Keywords:** radical ring-opening polymerization rROP, degradable vinyl-based polymers, cyclic ketene acetals CKA, thionolactones, lipoates, recycling, (bio)degradation, radical polymerization, cleavable comonomer, end of life (EoL)

## Abstract

Low weight, low price, and excellent long-term stability are the main advantages of vinyl-based polymers. Such polymers are obtained by chain-growth processes leading to all-carbon backbones, which are non­(bio)­degradable and nonchemically recyclable. Unfortunately, this chemical stability manifests as postuse persistence; coupled with poor waste management practices, polymers including vinyl derivatives pose major environmental problems today. Given that it is very difficult and costly to design entirely new materials that have both desired properties (mechanical, thermal, solvent resistance, etc.) and recyclability and/or biodegradability at the end of their life cycle, it seems worthwhile to transform already known materials into (bio)­degradable/chemically recyclable equivalents. One approach is based on the introduction of cleavable bonds into the polymer backbone, so that degradation (by hydrolysis, for example) produces oligomers which can then be further recycled and/or bioassimilated by micro-organisms. An effective method for incorporating weak bonds randomly into the C–C backbone of a vinyl polymer is the copolymerization of vinyl monomers with cyclic monomers by radical ring-opening polymerization (rROP). This method combines the advantages of ring-opening and radical polymerization, i.e., the production of polymers with heteroatoms and/or functional groups in the main chain, with the robustness, ease of use, and mild polymerization conditions of a radical process. The aim of this tutorial review is to provide polymer chemists with guidelines to use rROP to prepare vinyl-based materials with predictable degradation. This review thus presents the rROP principle, the main families of cyclic monomers copolymerizable with vinyl monomers, and the main applications of the resulting (bio)­degradable/chemically recyclable materials (polymers for packaging, latexes and degradable surfaces, 3D printing, biomaterials and water-soluble polymers).

## Graphical abstract

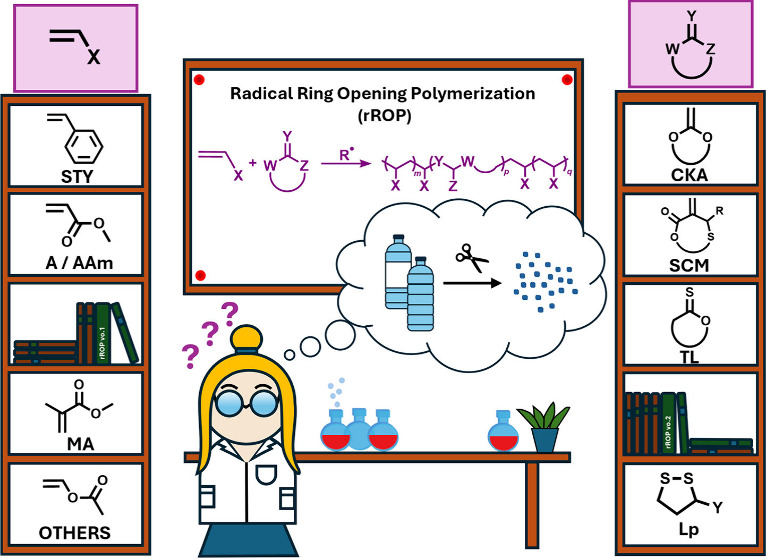

## Introduction

Radical polymerization is a widely used method for synthesizing polymers due to its simplicity and compatibility with a broad range of monomers. It relies on the formation and propagation of free radicals, enabling the production of polymers with diverse properties. Although historically a difficult-to-control process, achievements in the last three decades have significantly improved the ability to harness radical polymerization as a method to control the structure and properties of polymeric materials. Techniques such as reversible–deactivation radical polymerization (RDRP)^1,2^ including reversible addition–fragmentation chain transfer (RAFT),^3,4^ nitroxide-mediated polymerization (NMP),^5^ and atom transfer radical polymerization (ATRP)^6–8^ now allow for precise control over polymer molar mass with user-friendly reaction conditions. Similarly, progress in photopolymerization now facilitates control over chain growth spatiotemporally by accurately activating a reaction locally and/or temporally with exposure to light.^9,10^ In addition, both polymer functionalization and postfunctionalization methods have advanced considerably, making it possible to design materials with tailored properties that meet industrial demands.^11–13^ However, a major challenge remains: controlling the fate of polymers after use through (bio)­degradation and/or recycling.^14–16^

The stability of synthetic polymers leads to an accumulation of plastic waste in the environment, contributing to the issue of microplastics among other negative societal effects. In particular, vinyl polymers, produced via radical polymerization and composed of carbon–carbon (C–C) backbones, are non­(bio)­degradable. Their persistence after use and the poor management of plastic waste in general result in significant environmental concerns, such as ocean pollution caused by (micro)­plastics. Since developing entirely new materials remains challenging and costly, particularly ones that simultaneously exhibit desirable mechanical, thermal, and solvent resistance properties while also being recyclable or biodegradable, modifying existing materials to become biodegradable or recyclable may be a viable alternative to broadly address sustainability.^17^ While the cleavable comonomer approach is increasingly recognized as a promising strategy to address plastic pollution, its environmental benefits remain underexplored compared to conventional waste reduction, as end-of-life management of such new materials is still underdeveloped with only a few proofs of concepts on close-loop recycling and/or efficient biodegradation reported, leaving open questions about real-world bioassimilation, complete mineralization, and potential microplastic formation relative to conventional plastics.^17^

In this context, radical ring-opening polymerization (rROP) has emerged as a promising strategy.^18^ This method enables the introduction of cleavable bonds into the polymer backbone via copolymerization with cyclic monomers (Figure 1), allowing degradation (e.g., via hydrolysis) to produce oligomers that can subsequently be recycled or biodegraded by microorganisms. As a result, this approach has attracted growing interest over the past decade, leading to the synthesis of easily degradable polymers via radical pathways.

**Figure 1:**
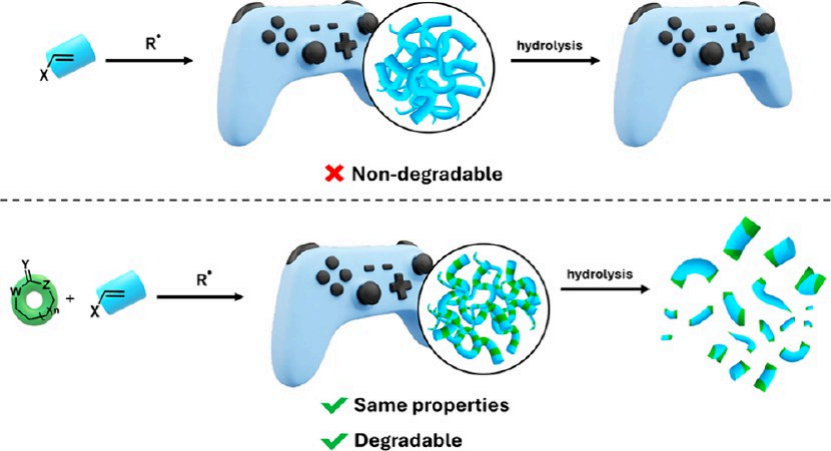
Radical copolymerization of cyclic and vinyl monomers aimed at developing degradable materials.

The addition of heteroatoms at the α position of the initial double bond, referred to as Z and W (Figure 1), allows for the incorporation of various chemical functionalities into the backbone of the polymer structure, such as ketones, esters, amides, or thioesters. Thus, rROP combines the advantages of ring-opening polymerization with those of radical polymerization, retaining the simplicity of a radical process while enabling the incorporation of more diverse functional groups into the polymer backbone. rROP is a technique distinct from conventional vinyl polymerization, primarily due to its two-step propagation mechanism and the use of cyclic monomers. It is also referred to as radical addition–fragmentation polymerization.^19^ A key feature of this type of polymerization is the presence of a double bond or double-bond analogue, which is essential for radical addition. Depending on the position of this double bond within the ring, monomers are generally classified into two main categories (Figure 2).

**Figure 2:**
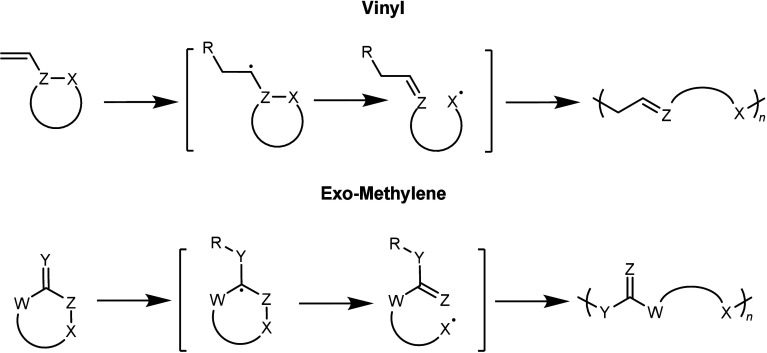
Structures of the two main categories of cyclic monomers that can be polymerized by radical ring-opening polymerization (rROP).

When the double bond is located at the α position of the ring, the compounds are referred to as vinylic monomers, which leads to the incorporation of double bonds along the polymer backbone. In contrast, when the double bond is in the *exo*-methylene position of the ring, the polymerization results in a polymer bearing a pendant double bond.

Since the first studies conducted in the 1980s, it has been shown that two specific conditions are essential for a cyclic monomer to undergo rROP.^18,19^ First, the formation of a thermodynamically stable C=Z group after ring opening is necessary.

Second, the radical formed after fragmentation must be favorably stabilized, which plays a critical role in the success of the reaction. One of the main challenges of rROP lies in the competition between two distinct reaction pathways. The radical produced after addition to the double bond, known as the adduct, can follow two different routes. It can either undergo ring opening via β-scission (a process also referred to as fragmentation or isomerization), thereby incorporating functional groups into the polymer backbone, which is the desired mechanism in rROP. Alternatively, the adduct can react directly with another monomer, leading to conventional vinyl polymerization (also known as 1,2-polymerization or direct polymerization). This latter route does not allow the introduction of functional groups into the polymer structure and results in an aliphatic polymer with pendant rings (Figure 3).

**Figure 3:**
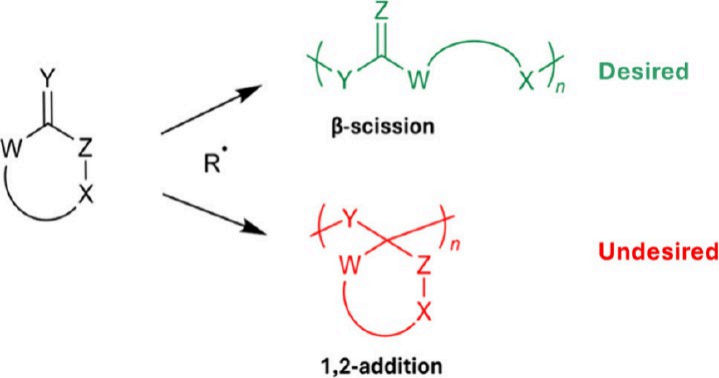
Competition between radical ring-opening (β-scission) and ring retention (1,2-addition).

Therefore, it is crucial to control the reaction mechanism to optimize the functionalization of polymers produced by rROP and to fully harness the potential of this method.

This tutorial review aims to provide an overview of recent advances in radical ring-opening polymerization and to demonstrate how these developments contribute to addressing the challenge of degrading synthetic polymers produced via radical pathways. This article is divided into two sections. The first one presents various cyclic monomers and their synthesis with experimental details provided in tables throughout the manuscript to specifically advise users of the best reaction conditions for polymerization. In a second section, the review offers a didactic overview of the applications made possible by these techniques, focusing on the specific degradation conditions as well as the end-of-life (biodegradation or recycling) of these materials.

## History of rROP

rROP originated in the 1960s, with the first studies conducted on fully carbon-based monomers, which at that time did not yet allow the production of degradable polymers. In 1961, Errede^20^ carried out the first radical polymerization of a *spiro*-di-*o*-xylylene, demonstrating the possibility of forming a semicrystalline polymer through a specific radical approach. At the same time, Van Volkenburgh^21^ suggested that vinyl cyclopropane could polymerize under the influence of peroxides and UV light, a hypothesis confirmed a few years later by Takahashi and Yamashita,^22^ who demonstrated a 1,5-ring-opening mechanism. This early work produced low molecular-weight polymers between 1000 and 5000 g/mol, leading to further research aimed at making monomers more reactive through the addition of substituents.

In 1975, a breakthrough was made by Bailey and Endo,^23^ who successfully carried out the first radical ring-opening polymerization of a heterocycle, the unsaturated spiro-ortho-carbonate (**SOC**). By heating this monomer at 130 °C in the presence of peroxide, they obtained a polycarbonate-*alt*-polyether with pendant double bonds, which favored cross-linking. This work marked the beginning of the integration of heteroatoms into rROP monomers, opening the door to new dual ring-opening polymerization strategies, which enable the incorporation of various functionalities into the polymer structure.

In the 1980s, Bailey and Endo^24^ continued their work with the polymerization of *spiro*-ortho-esters (**SOE**), demonstrating the production of a poly­(ketone-ester) via radical initiation. At the same time, Bailey explored Maillard’s work on the radical ring-opening of 1,3-dioxolane^25^ and found that the addition of olefins led to various addition and transfer products, suggesting a complex radical isomerization mechanism.^25^ This discovery further fueled interest in the radical polymerization of heterocycles and led to research on other monomers. A significant advancement occurred when Bailey et al.^26^ turned their attention to cyclic ketenes acetals (**CKA**), compounds that were initially unstable and difficult to handle due to their spontaneous polymerization.

In 1982,^26^ Bailey et al. demonstrated that the double bond of CKA could enable effective radical polymerization, particularly with 2-methylene-1,3-dioxolane and methylene-1,3-dioxepane (**MDO**). These works led to the radical synthesis of polyesters, a key milestone in the development of biodegradable polymers through rROP. In the following years, new classes of monomers emerged, gradually expanding the field of rROP. In 1983, Cho’s group introduced vinyl oxiranes.^27^ In 1985, Bailey introduced cyclic vinyl ethers (**CVE**), thus opening new avenues for polymerization.^28^ The following year, Cho’s team explored sulfur-containing monomers with cyclic vinylsulfones,^29^ while in 1987, Bailey refined his research by focusing on cyclic α-oxyacrylates (**CαOA**).^30^ In 1994, Rizzardo and his team developed the rROP of sulfides cyclic methacrylate (**SCM**),^31^ followed in 1996 by cyclic allylic sulfides (**CAS**).^32^ In 2015, Tsarevsky’s group^33^ reported the rROP of lipoates (**Lp**) that was later developed by Bates and co-workers.^34^ In 2018, Niu et al.^35^ reported a second generation of SCM that avoids undesired cross-linking due to the presence of the exomethylene present in the monomer by also replacing the propagation of the thiyl radical by a classic carbon radical via the extrusion of SO_2_. Finally, in 2019, a turning point was marked when Roth et al.^36^ and Gutekunst et al.^37^ simultaneously achieved the first rROP of thionolactones (**TL**).

Thus, over the years (Figure 4), rROP has evolved into a key method in the design of degradable polymers, thanks to the strategic incorporation of heteroatoms and fragile bonds into the polymer structure.

**Figure 4:**
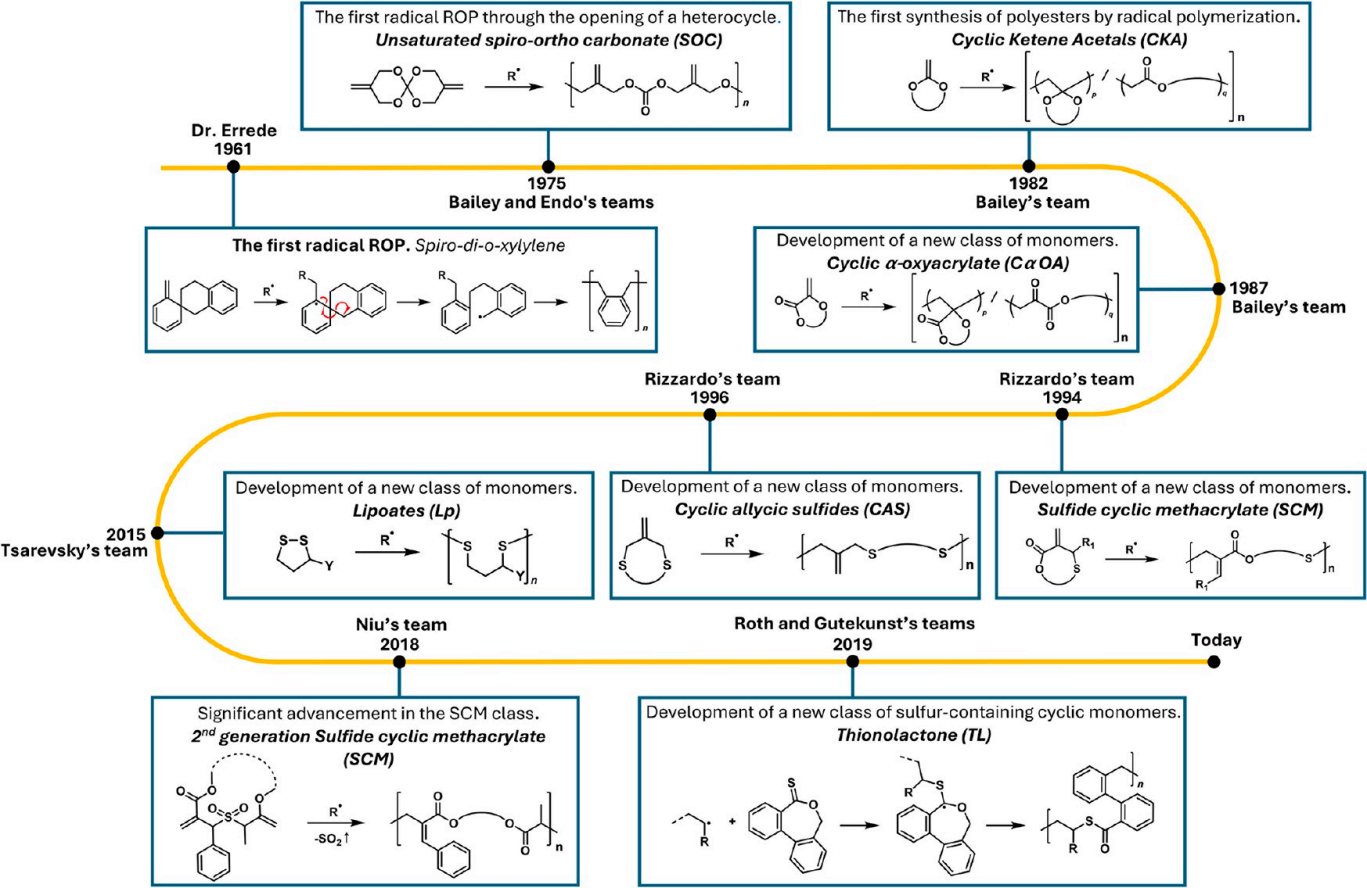
Timeline of the history of rROP with the different families of monomers used.

Research has focused on the identification of new monomers polymerizing via addition–fragmentation, which has allowed for refined control over the structure and properties of resulting polymers. These advancements marked a major transition in radical polymerization, opening new perspectives for the synthesis of functional and degradable polymers.

## Main Monomer Families

Among the different cyclic monomers that have been tested since the late 1970s, only a few monomer families have been extensively studied in the literature and used to prepare materials with degradable features. The first family, both historically and by the number of publications, is cyclic ketene acetals (CKAs).^18,38–40^ The main interest of this family is to obtain, after ring-opening, an ester unit in the polymer backbone whose reactivity and degradability are already well established. Nevertheless, many drawbacks, such as inherent monomer instability, copolymerization behavior, etc., impede these monomers from being used on a large scale, even if industrial production of some monomers is possible.

The second interesting family is a sulfide cyclic methacrylate. The first members of this family were described and used by Rizzardo et al.^31^ and later by Hawker and colleagues.^41^ They were easily prepared (see below) and could copolymerize well with (meth)­acrylic derivatives, but homopolymerization and copolymerization at high feed ratios are hampered by the exomethylene functionality inserted in the backbone of the polymer that leads to cross-linking. This drawback was later solved by Niu and co-workers^35^ who modified the structure by inserting a phenyl ring that impedes the further addition of a propagating macroradical causing the cross-linking. In the same study, Niu et al.^35^ showed that the oxidation of the sulfide group to sulfone enabled replacing the thiyl radical with a carbon-based radical following intramolecular extrusion of SO_2_ (not being detrimental to the polymerization), thus facilitating control of the polymerization. Huang et al. recently proposed a different mechanism based on classic thiyl radicals that react intermolecularly with either isocyanides^42^ or trivalent phosphorus compounds^43^ (triethyl phosphite for example) to undergo desulfurization and generate a stabilized alkyl radical for reversible control. Recently the same author reported a new RAFT agent for controlling the thiyl propagating macroradicals.^44^ These structures, albeit obtained via a multistep synthesis, are particularly efficient.

To increase the copolymerization efficiency between vinyl and cyclic monomers, Roth et al.^36^ and Gutekunst et al.^37^ prepared thionolactone that bears a C=S group instead of an *exo*-methylene bond to enhance their radical accepting ability. Efficient copolymerization with many vinyl-based monomers was achieved, and different modes of degradation are accessible (i.e., basic hydrolysis, aminolysis, red-ox, etc.). The radical homopolymerization is nevertheless difficult.

Lastly, inspired by early work from Stockmayer et al.,^45^ Tobolsky et al.,^46^ and Endo et al.^47,48^ on the copolymerization of various disulfides with vinyl monomers, Tsarevsky et al.^33^ reported the copolymerization of lipoate derivatives with vinyl monomers. Recently Bates, Hawker, Read de Alaniz, and co-workers^49^ developed this approach to prepare degradable materials. A major advantage of this monomer family is the commercial availability of α-lipoic acid, which is bioderived, edible, and sold as a consumer supplement for ∼$0.10/g. As will be discussed in detail below, copolymers of α-lipoic acid contain both S–S and C–S bonds along the backbone, meaning the sequence of diads and degradation conditions play important roles in determining the maximum possible decrease in molecular weight upon cleavage.

In the following sections, we will present the synthesis of cyclic monomers compatible with radical ring-opening polymerization, their homopolymerization, and various reaction possibilities to obtain copolymers, broadly classified by vinyl monomer chemistry. Lastly, some applications will be presented as well as a specific section on (bio)­degradation and recycling.

## Monomer Syntheses

This section of the review focuses on the synthesis of various monomers, classified by family, and highlights those that are most commonly used due to their effectiveness.

### Cyclic Ketene Acetals (CKA)

Within the family of cyclic ketene acetals (CKA), numerous monomers have been synthesized in recent years and are extensively documented in the scientific literature.^18^ However, many of these monomers are not optimal for radical ring-opening polymerization aimed at obtaining degradable polymers. Indeed, their difficulty in undergoing ring opening, leading to ring retention, as well as their poor reactivity with most vinyl monomers, prevents effective degradation of the final polymer. Furthermore, their stability is often complicated by strict requirements in terms of protic conditions.^50^ Therefore, our focus here will be on CKAs specifically studied for degradation performance, with an emphasis on those whose ring-opening is well-controlled and does not result in cationic polymerization (Figure 5). The following research will therefore focus almost exclusively on a few monomers that are also the most studied: 2-methylene-1,3-dioxepane (MDO), 5,6-benzo-2-methylene-1,3-dioxepane (BMDO), 2-methylene-4-phenyl-1,3-dioxolane (MPDL), 2-methylene-1,3,6-trioxocane (MTC), as well as a CKA based on a monosaccharide unit (Glu-CKA). MDO is now commercially available from different chemical providers.

**Figure 5:**
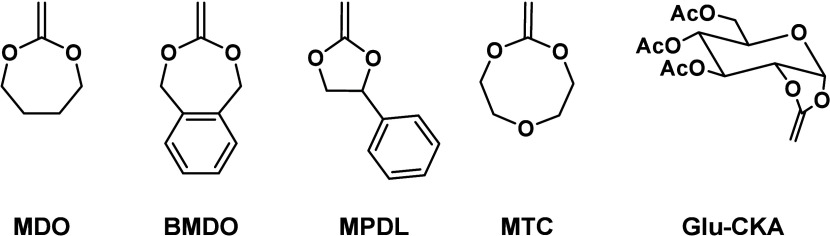
Structures of the most efficient CKAs in rROP.

The first description of the main synthetic method for cyclic ketene acetals (CKA) dates to 1948 and is attributed to McElvain.^51^ This approach is based on an acid-catalyzed transacetalization, such as using *p*-toluenesulfonic acid (pTSA) or DOWEX resin, involving a reaction between dimethyl chloroacetal and diol (Figure 6). Since this reaction is reversible, the removal of generated methanol is essential to promote the formation of cyclic chloromethyl acetal. It is also possible to use diethyl chloroacetal, but this results in the formation of ethanol as a byproduct, which can complicate the process due to its higher boiling point.

**Figure 6:**
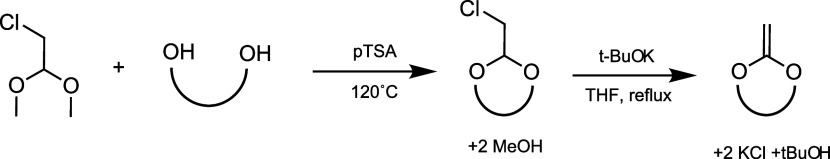
Synthesis of CKA via the transacetalization and dehydrochloration reaction.

The second step of the synthesis involves the elimination of HCl to generate an exomethylene unit, a transformation typically carried out in the presence of a strong base such as KOH or potassium *tert*-butoxide (*t*-BuOK). In some cases, the bromo derivative is preferred, as it facilitates the dehydrohalogenation reaction, although its cost is higher than that of its chlorinated counterpart, which is commercially available. The influence of the haloacetaldehyde dimethyl acetal used in this process was examined by Nicolas and his team during the synthesis of MPDL.^52^ Their study revealed that the yield of the transacetalization remained relatively stable (65% with chloroacetal vs 80% with bromoacetal), but the dehydrohalogenation phase showed notable variations. At 0 °C and after 2 h of reaction, the yield of MPDL ranged from 23% for chloroacetal to 81% for bromoacetal. This yield even reached 85% in just 1.5 h when the iodoacetal derivative was used, which was formed in situ by reacting to bromoacetal with NaI in acetone.^52^

CKAs can also be synthesized via two alternative routes: the acetal route, optimized using the CoCl_2_/TMSCl catalytic system,^53^ which provides good yields at room temperature, and the carbonate route^53,54^ using the Petasis reagent,^55^ which is less versatile and requires greater synthetic effort (Figure 7). Compared to the classical method, this new acetal route improves yields for seven-membered rings, while the carbonate route remains less efficient and more complex.^53^ Gaitzsch^56^ and co-workers used this route to prepare amine-bearing CKAs for preparing pH-responsive polyesters. These structures were not copolymerized with vinyl monomers. Other synthetic methods have also been reported in the literature, but they are rarely used. A notable distinct route to CKA derivatives is the synthesis of Glu-CKA, a fusion of a five-membered CKA with a monosaccharide pyranose structure. Niu’s group,^57^ building on the work of Hecht and Ko,^58,59^ developed an approach that yields Glu-CKA in 94% over just two steps.

**Figure 7:**
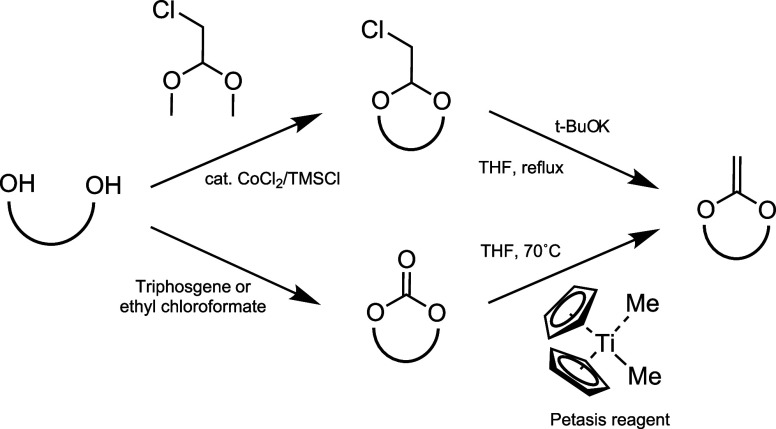
Two other synthesis pathways: a new acetal pathway and carbonate pathway.

This method relies on d-glucose pentaacetate, an inexpensive and readily available precursor, and involves anomeric bromination followed by a nucleophilic attack from the neighboring 2-O-acetate group and then deprotonation. Without requiring column chromatography, this strategy is easily scalable and allows the synthesis to be completed in just 3 h in 94% overall yield over two steps (Figure 8).^57^

**Figure 8:**
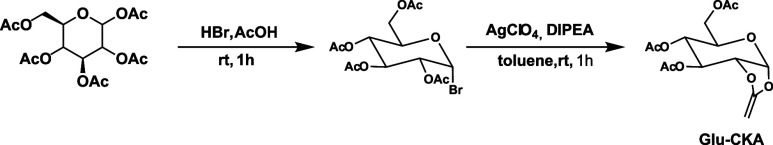
Synthesis pathway of Glu-CKA.

It is also worth noting that Buchard’s group recently reported the synthesis of a cyclic ketene acetal (CKA) derived from d-glucal via the acetal pathway.^60^ However, these monomers were not very efficient since close to 50% were inserted with ring retention.

### Sulfide Cyclic Methacrylate (SCM)

In 1994, Rizzardo and his team^31^ developed a new family of cyclic monomers known as sulfide cyclic methacrylates, sometimes referred to as macrocyclic allylic sulfides (Figure 9). The inherent instability of the C–S bond promotes β-scission, leading to the formation of a C=C double bond and a thiyl radical that continues the propagation. Early research focused on specific structures, highlighting that polymerization occurred exclusively via ring-opening, in contrast to other classes of monomers.^61^ The polymerization mechanism revealed that the ring size slightly influences monomer reactivity, with larger rings exhibiting a higher rate of polymerization.^62,63^

**Figure 9:**
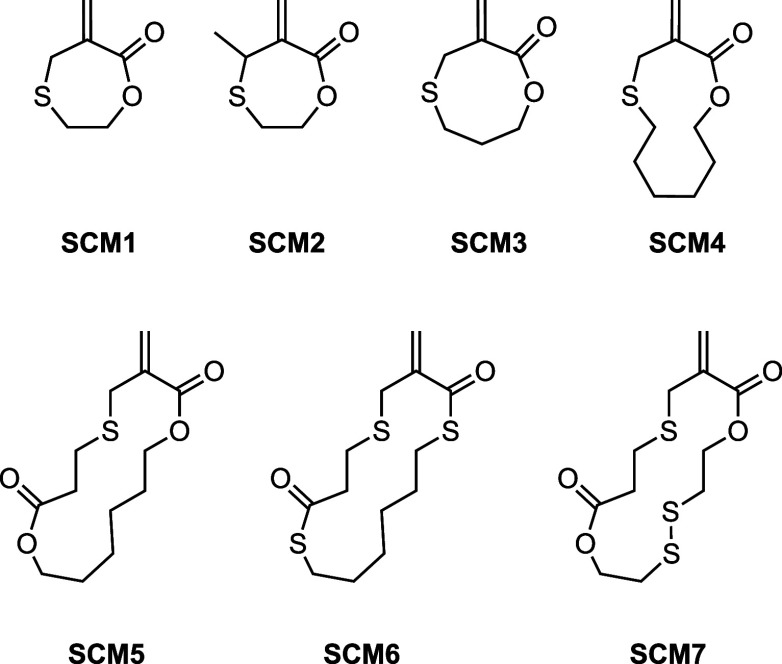
Structures of sulfide cyclic methacrylate monomers.

Subsequently, Hawker et al.^41^ expanded this work by incorporating cyclic monomers bearing various functional groups such as esters, thioesters, and disulfides into their side chains. The synthesis of these compounds is carried out in several steps, typically starting with the reaction of 2-bromomethyl acrylic acid with a thiol alcohol, yielding an α-carboxy-ω-hydroxy-functionalized compound. The monomer is then obtained through an intramolecular esterification performed under high dilution conditions (Figure 11a).

A representative example is 3-methylene-1,9-dioxa-5,12,13-trithiacyclopentadecan-2,8-dione (MDTD or SCM4).^41^ Its synthesis is based on a nucleophilic substitution between 2-bromomethyl acrylic acid and 6-mercapto-1-hexanol to obtain a hydroxyl acid, followed by cyclization under high dilution conditions using the Mukaiyama reagent, resulting in an overall yield of 30%. This methodology can be adapted to design a broader range of monomers incorporating various functional groups (ester, disulfide, thioether, or silyl ether), by adjusting the nature of the intermediate hydroxyl acid and selecting the appropriate diol or dithiol.^41^

More recently, research conducted by Niu et al.^35^ has led to significant advancements, notably through the oxidation of the thioether to a sulfone and the modification of the alkyl side group, including the addition of a phenyl group in the α-position of the double bond (Figure 10A). The synthesis of these compounds involves introducing a phenyl group at the α-position of the double bond by coupling benzaldehyde with methyl acrylate via the Morita–Baylis–Hillman reaction (Figure 11b).

**Figure 10:**
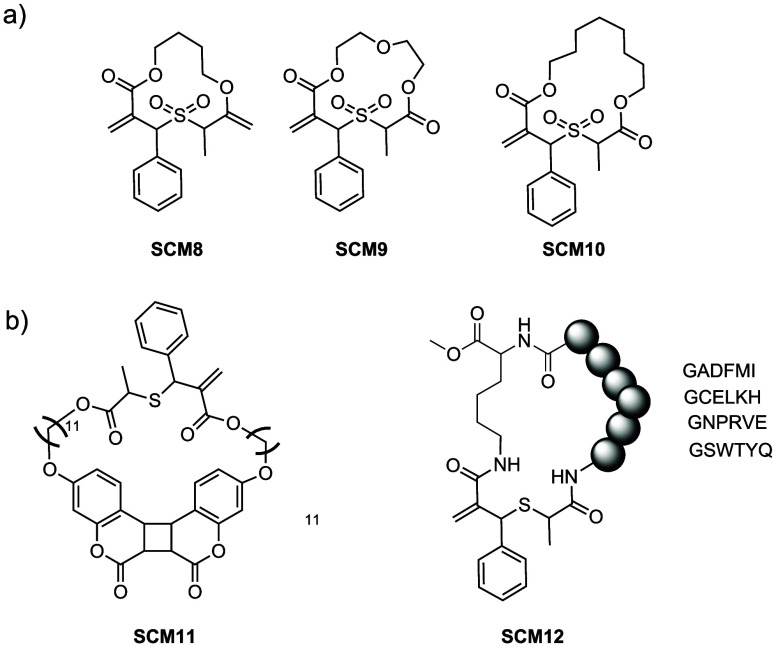
(A, B) Structures of second-generation sulfide cyclic methacrylate monomers by Niu et al.^35^ and Frisch et al.^64,65^

**Figure 11:**
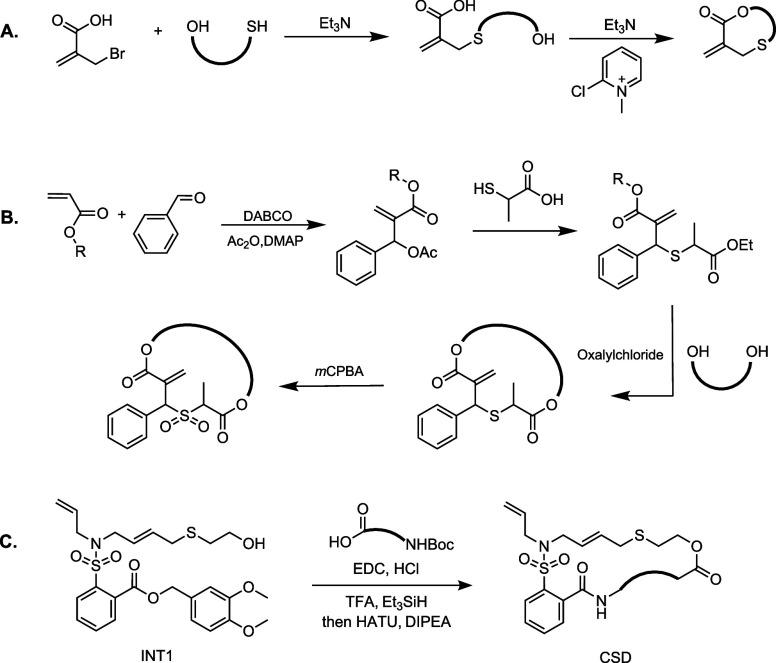
Synthesis of sulfide cyclic methacrylate-type monomers (SCM). a) First generation, b) second generation, and c) cyclic sulfide diene (CSD).

After protecting the hydroxyl group using acetic anhydride, the resulting intermediate reacts with a mercaptoester to form a thioether. Finally, the last step involves cyclizing the α,ω-esterified compound in the presence of diols. Frisch and co-workers extended the cyclization from diols to peptides^64^ or photoadducts^65^ (Figure 10B) for various applications. Besides this structure, Huang, Niu, et al.^66^ proposed a new scaffold combining 1,6-diene with allylic sulfide or allylic sulfone motifs.

This structure facilitated a ring-closing/ring-opening cascade reaction that significantly promotes the ring-opening polymerization of large macrocyclic monomers. Later Huang^67^ and co-workers propose to introduce an allyl group replacing the acryl moiety, and the methyl group that is supposed to block the radical addition onto the produced exomethylene functionality into the backbone was omitted. Compound INT1 as a precursor was synthesized from commercially accessible saccharin after 3 steps with an overall yield of 36%. Since then, many functional monomers cyclic sulfide diene (CSD) were prepared (Figure 11c).

Although more complex than the synthesis of CKAs, this approach offers great structural diversity, which is a major asset for the development of advanced materials.

### Thionolactone (TL)

Thionolactones represent a particularly interesting class of monomers for radical ring-opening polymerization (rROP), as they are not based on a methylene bond. Their structure, characterized by a C=S double bond, gives them a remarkable ability to trap radicals by a mechanism similar to RAFT-type polymerization. Rizzardo et al.^68^ also highlighted the essential role of thionoesters as transfer agents, thereby facilitating the incorporation of a thioester unit at the end of polymer chains. The basic structure of thionolactone monomers is based on the same thionoester function, but with a cyclic structure to continue chain growth of the macroradical. When a radical adds to the thionoester (C=S)–O, β-scission occurs to generate the corresponding thioester (C=O–S), which is accompanied by a visible color change from yellow to colorless. Thionolactones are typically obtained by thionation of precursor lactones. This transformation is mainly carried out using Lawesson’s reagent, although alternative methods have been described, notably the use of P_4_S_10_ in the presence of hexamethyldisiloxane (HMDSO).

To date, only a few thionolactones possess the necessary properties, in terms of reactivity and ring-opening ability, to enable the formation of sufficiently degradable copolymers. As such, subsequent research has focused almost exclusively on a few monomers: dibenzo­[*c,e*]-oxepine-5­(7*H*)-thione (DOT),^36,37^ 7-phenyloxepane-2-thione (POT),^69^ and 10-fluoro-7-(4-(trifluoromethyl)­phenyl) DOT (F-*p*-CF_3_PhDOT).^70^ Among them, DOT is the most used thionolactone in the literature.

It is also reported that thionolactone monomers derived from either ε-caprolactone^71,72^ or glycolide and lactides, such as dl-thionolactide, thionolactide, and dl-dithionolactide,^73–75^ have been used. These monomers are of interest due to the biobased nature of the corresponding lactone and their straightforward synthesis, which relies on a simple thionation reaction, as the corresponding lactones are readily available commercially. However, when copolymerized with various vinyl monomers, these compounds exhibit both ring opening and 1,2-thiocarbonyl propagation (ring retention). Despite this, they still enable relatively efficient degradation of the resulting copolymers, particularly after treatment with bleach.^74,75^

The original synthesis^36,37^ of dibenzo­[*c,e*]-oxepine-5­(7*H*)-thione (DOT) begins with the reduction of diphenic anhydride using NaBH_4_ in anhydrous DMF, followed by acid-catalyzed intramolecular esterification (Figure 12).^76^ After extraction and purification by column chromatography, dibenzo­[c,e]­oxepine-5­(7*H*)-one (DOO) is obtained as white crystals with a 70% yield. In a second step, this lactone undergoes thionation using Lawesson’s reagent in dry acetonitrile heated to 90 °C or toluene at 115 °C.^77^ After filtration, solvent evaporation, and purification by chromatography followed by recrystallization, dibenzo­[c,e]­oxepane-5­(7*H*)-thione is isolated as yellow crystals with a yield of 38%. Recently Johnson et al.^78^ revisited the synthesis of DOT (Figure 12), using a combination of the Tishchenko reaction with Ullmann coupling from 2-bromobenzaldehyde derivatives in the presence of zinc to obtain the corresponding lactone. This approach allowed the preparation of various DOT derivatives (F, OMe, SPr) with different substituents on one or both aromatic rings.^78^

**Figure 12:**
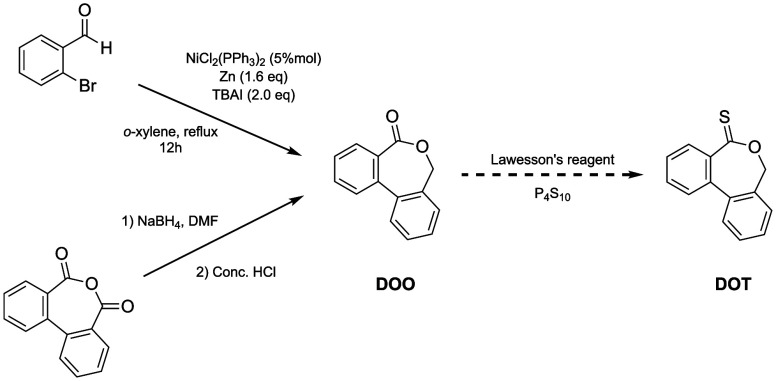
Synthesis of dibenzo­[*c,e*]-oxepine-5­(7*H*)-thione (DOT).

Recently, Guillaneuf and co-workers^69^ reported 7-phenyloxepane-2-thione (POT) that could be synthesized in two steps. First, a Baeyer–Villiger oxidation was performed on 2-phenylcyclohexanone to obtain the corresponding lactone, following a previously published procedure (yield of 93%). Then, the lactone was thionated using Lawesson’s reagent in anhydrous toluene, yielding the POT monomer with a 50% yield (Figure 13), in the form of a brown oil that solidifies upon cooling. The production cost of POT is approximately 50% lower than that of DOT. Para-functional POT derivatives were synthesized.^79^

**Figure 13:**
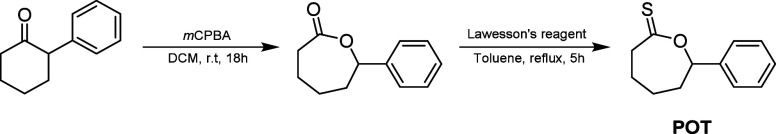
Synthesis of 7-phenyloxepane-2-thione (POT).

Only electron withdrawing groups were inserted (CF_3_ and NO_2_), whereas the thionation of the lactone bearing electron donating groups was unsuccessful.^79^

10-Fluoro-7-(4-(trifluoromethyl) phenyl) DOT (F-*p*-CF_3_PhDOT) is the only thionolactone reported to copolymerize efficiently with methacrylate derivatives.^70^ Its synthesis begins with a palladium-catalyzed Suzuki coupling reaction, using 2-bromostyrene and a fluorinated 2-formylphenylboronic acid as starting reagents. This coupling leads to the formation of the coupling product, 2-vinyl-[1,1-biphenyl]-2-carbaldehyde. Once this compound is obtained, a Grignard reaction is carried out, during which a specific Grignard reagent is added to introduce the fluoro-benzyl substituent. An oxidative lactonization^80^ is then performed, followed by thionation with Lawesson’s reagent, yielding the final F-*p*-CF_3_PhDOT product. This four-step synthesis results in a relatively low overall yield of 13% (Figure 14).^70^

**Figure 14:**
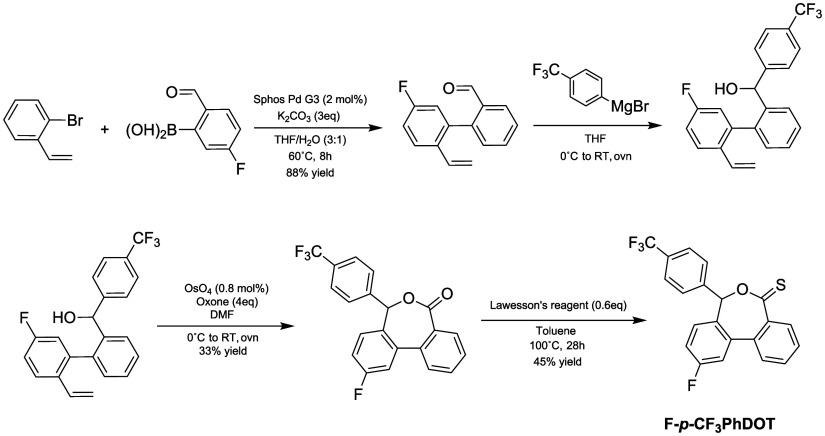
Synthesis of 10-fluoro-7-(4-(trifluoromethyl) phenyl) DOT (F-*p*-CF_3_PhDOT).

### Lipoates

One of the main challenges with the systems described above lies in the need for multistep syntheses to prepare the cyclic comonomers. In contrast, the use of lipoates derived from α-lipoic acid (αLA) offers a clear advantage. This natural compound, found in certain vegetables and involved in redox biological processes, is commonly used as a dietary supplement. It is widely available in the consumer market, produced industrially at a scale of approximately 250 tons per year, and is inexpensive (a few cents per gram). αLA contains a 1,2-dithiolane ring, which is capable of undergoing ring-opening polymerization under the influence of radicals, heat, or UV light. Under suitable conditions, the generated thiyl radicals interact with other dithiolane units to form polymer chains containing disulfide bonds. This property has generated increasing interest, particularly in applications such as elastomers, self-healing materials, and chemical recycling. Nevertheless, although disulfide bonds are degradable, they are also dynamic under the influence of heat, light, or certain bases, which may compromise the thermomechanical stability of αLA-based materials. To date, relatively few studies have explored the use of lipoates in rROP. Only a few monomers have been reported, with simple and well-documented syntheses. In particular, α-lipoic acid (which requires no synthesis) and ethyl lipoate, an ethyl ester of α-lipoic acid, are notable.

The latter was prepared via a classic esterification route using DCC/DMAP coupling (Figure 15) and helps overcome solubility issues associated with αLA and increases the proportion of degradable units incorporated into the resulting polymers. Dove and co-workers^81^ prepared various biosourced multifunctionalized lipoates (Figure 15) via esterification of lipoic acid and use such compounds as UV-printable resins to prepare degradable thermoset 3D objects.

**Figure 15:**
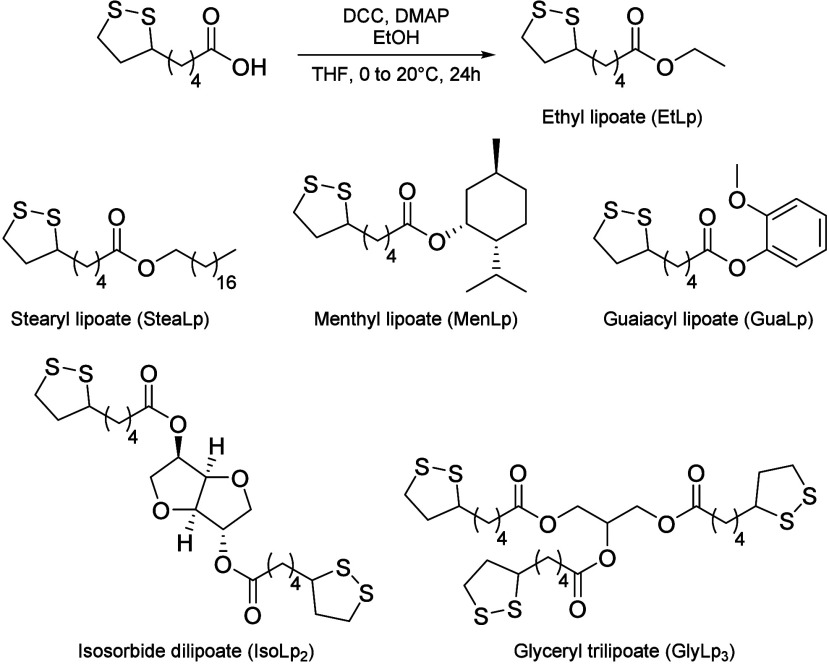
Synthesis of ethyl lipoate and structures of monomers previously reported in the literature.

## Homopolymerization via rROP

The radical homopolymerization of CKAs has been extensively studied since the 1980s, notably by Bailey and his collaborators.^26^ When carried out under appropriate conditions, it enables the formation of aliphatic polyesters via a radical pathway. However, a major issue lies in the competition between ring-opening (desirable) and direct vinyl propagation (undesirable), which leads to the formation of nondegradable polyacetals.^82^ Numerous CKA monomers have been investigated to optimize ring opening based on various parameters. Nevertheless, identifying conditions that promote complete ring opening remains challenging, as results vary depending on experimental conditions such as temperature, solvent, the nature and concentration of the radical initiator, and the characterization techniques used (NMR, FTIR, etc.). Moreover, it has been difficult to clearly establish the relationship between the monomer structure (ring size, stability of the formed radical, and the variety of alkyl substituents) and reactivity, which would help to better understand the ring-opening process.^39,83^ By considering only the ring-opening reaction, it has not been possible to rationalize the ring-opening efficiency of CKA monomers, since this feature is a result of a complex mechanism. The reactivity of CKAs in homopolymerization has been studied through a combination of quantum modeling (DFT) and numerical simulations, enabling the correct prediction of the kinetic competition between ring-opening and ring-retaining pathways (Figure 16).^82^ Unlike previous studies,^83^ which focused solely on ring opening, this approach considers the entire reaction sequence: bimolecular radical addition to the CKA, unimolecular fragmentation (ring-opening), and bimolecular propagation of the radicals to another CKA. This methodology now makes it possible to predict the percentage of ester functionalities within a polymer chain and to optimize the reactivity of CKA monomers in homopolymerization.^82^

**Figure 16:**
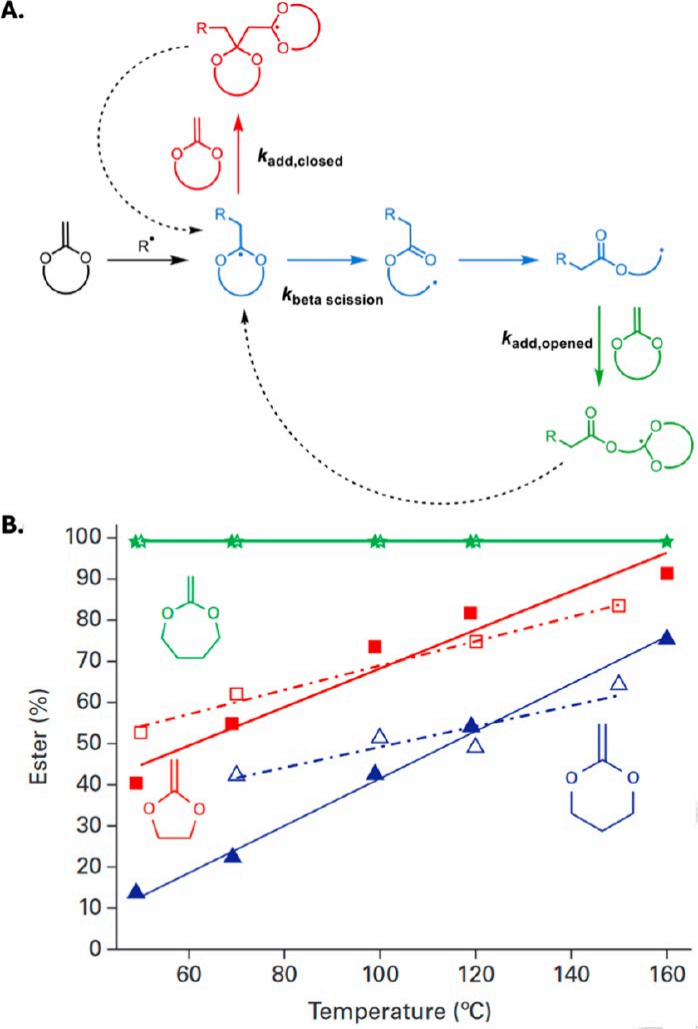
(A) Kinetic competition between vinyl propagation and ring opening. (B) Percentage of ring opening for 5-, 6-, and 7-membered CKA monomers (filled points: experimental data; empty points: theoretical data). Reproduced from ref 82 with permission. Copyright 2020 Wiley-VCH.

Despite these uncertainties, three monomers have proven to produce pure polyesters: MDO (methylene-1,3-dioxepane), BMDO (5,6-benzo-2-methylene-1,3-dioxepane), and MPDL (2-methylene-4-phenyl-1,3-dioxolane), all known to undergo complete ring opening under a wide range of experimental conditions. Furthermore, Thoniyot et al.^84^ confirmed that large-ring CKAs (7–8-membered rings) undergo ring opening more efficiently than smaller-ring counterparts (5–6-membered rings). However, the homopolymerization of MDO produces a polymer similar to PCL, but the radical mechanism leads to hydrogen transfer at the 1,5 and 1,7 positions, causing intra- and intermolecular branching. This prevents crystallization, as demonstrated by Jin and Gonsalves,^85^ and later confirmed by Agarwal et al.^86^ and Guillaneuf et al.^87^ This characteristic has been exploited by researchers to tune crystallinity (see Applications section for details).

To date, most CKAs remain difficult to homopolymerize via conventional free-radical polymerization (FRP), often requiring harsh conditions (long reaction times, low molar masses). To overcome these limitations, controlled radical polymerization (RDRP) methods have been explored. Nicolas and Guillaneuf et al.^88–92^ investigated the compatibility between rROP and NMP, while Jackson and collaborators used RAFT polymerization for macromolecular engineering of CKAs.^93–95^ The monomer developed by the Niu group, Glu-CKA, exhibits good homopolymerization within 24 h when initiated by AIBN, yielding polymers with a number-average molar mass (*M*_n_) of 21,000 g·mol^–1^.^57^

### SCM Homopolymerization

Rizzardo and his team introduced a new category of sulfur-containing cyclic monomers known as sulfide cyclic methacrylates (SCM).^31^ These compounds polymerize exclusively via a ring-opening mechanism (Figure 17). They exhibit particularly high reactivity in homopolymerization, reaching nearly 80% conversion after just 3 h at 70 °C.^31^ However, in the case of the SCM1 monomer, premature cross-linking was observed as early as 20% conversion.^31^ In contrast, this issue does not occur with the SCM2 monomer, whose structure includes a methyl group positioned on the double bond resulting from the ring opening, thereby blocking any secondary radical addition. Polymerization mechanism analysis also revealed that ring size plays a role in monomer reactivity: larger rings lead to slightly faster polymerization while reducing the tendency toward cross-linking.

**Figure 17:**
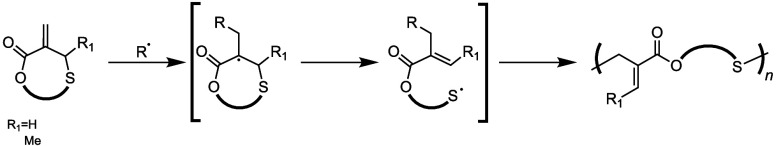
Mechanism of radical polymerization via ring-opening of sulfide cyclic methacrylates (SCM1–SCM7).

Subsequently, significant progress was made by Niu et al.,^35^ who developed new monomers (named SCM 8 to SCM 10) by oxidizing the thioether group to a sulfone and modifying the alkyl side chain. The introduction of a phenyl substituent at the α-position of the double bond helps prevent cross-linking reactions. During polymerization, a radical cascade is initiated: the initial radical undergoes SO_2_ elimination (Figure 18a), leading to a stabilized acrylate-type radical, compatible with controlled polymerization techniques, particularly reversible deactivation radical polymerization (RDRP).^35^

**Figure 18:**
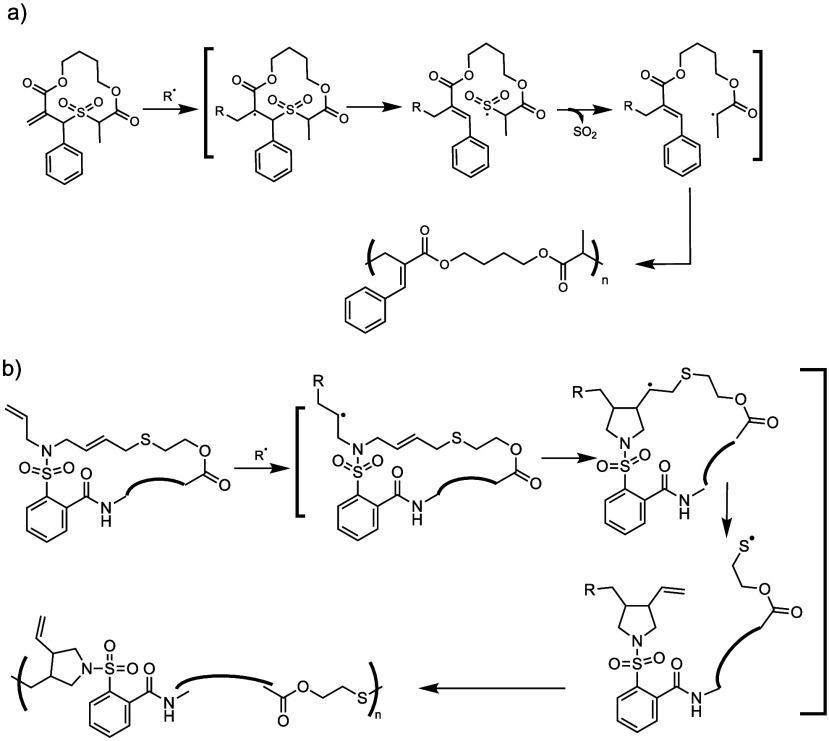
a) Mechanism of radical polymerization via ring-opening of sulfide cyclic methacrylates (SCM8–SCM10). b) Mechanism of radical polymerization via ring opening of CSD monomers.

Huang and co-workers^96^ reported the very efficient homopolymerization of various second generation MCS initiated by trialkylborane/oxygen, that led to high conversion in minutes at room temperature. Lastly the same authors reported the homopolymerization of the CSD monomers (Figure 18b), having different ring sizes and functionality showing the interest of this new platform.^67^ Controlled polymerization of such monomers could be obtained either by in situ desulfurization using isocyanides^42^ or phosphites^43^ or by adding a special SRAFT controlling agent.^44^

### Thionolactone Homopolymerization

The available data on the homopolymerization of thionolactones remains limited. Roth et al.^97^ reported that DOT poorly homopolymerizes, with a conversion rate of less than 10% after 7 days at 60 °C. They also studied the free radical polymerization of DBT initiated by AIBN, which achieved a conversion of 35% after an overnight reaction at 70 °C.^98^ Although the obtained polymer was characterized by NMR, its analysis by SEC was not performed due to solubility issues.^98^

To overcome DOT’s poor ability homopolymerize, Roth and co-workers^99^ present a “single-unit comonomer insertion” strategy to produce majority-DOT polymers containing large backbone regions of poly­(thioester) functionality. Owing to AIBN’s poor ability to initiate DOT, the addition of a reactive comonomer was theorized to produce secondary radical species capable of initiating DOT rROP. In a series of copolymerization trials, the addition of 5–10% comonomer feed of diethylvinyl phosphonate (DEVP) boosted monomer conversion to afford low-dispersity “homo”DOT chains containing 98–99% DOT.

Reineke et al.^100^ reported the use for rROP of thionoisochromanone (TIC), a derivative from a fungi-accessible lactone. Besides its use in copolymerization, its homopolymerization was also examined, and, like DOT, it was found to be slow, with a conversion reaching 75% after 8 days at 70 °C in DMF (Figure 19). Furthermore, the formed polythioester had a relatively low molar mass, ranging between 2000 and 7500 g·mol^–1^. Guillaneuf et al.^69^ reported the homopolymerization of POT at 120 °C in the presence of anisole (33 mol %) to improve the solubilization of the azo initiator VAM-111 (0.5 mol %). It was shown by ^1^H NMR that complete conversion was obtained after 22 h, resulting in the formation of a waxy polymer (Figure 20). The obtained polythioester exhibited a *M*_n_ of 5500 g·mol^–1^, a molar-mass dispersity (*Đ*) of 2, and a glass transition temperature (*T*_g_) between 1 and 2 °C.

**Figure 19:**
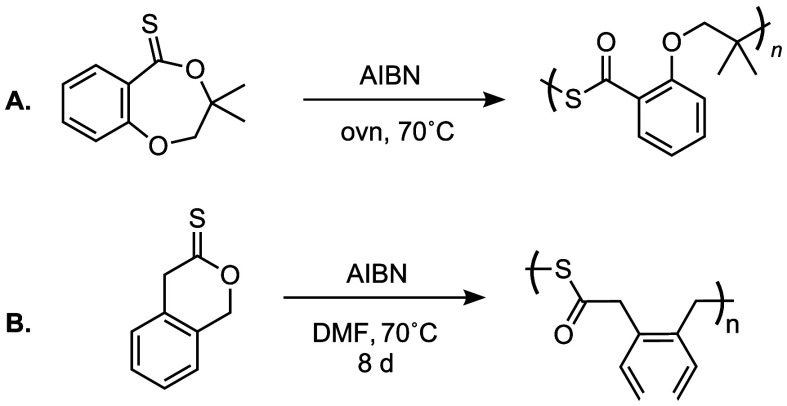
Homopolymerization of (A) DBT and (B) TIC.

**Figure 20:**
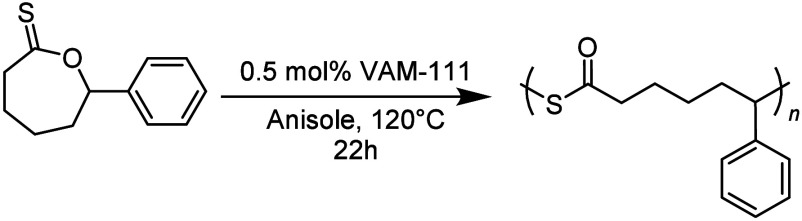
Homopolymerization of POT.

### Lipoate Homopolymerization

Polydisulfides synthesized by the ring-opening polymerization (ROP) of 1,2-dithiolanes readily undergo chemical recycling to yield monomers, commonly utilizing lipoic acid as a cost-effective biogenic feedstock. This monomer may be polymerized using anionic, cationic, and radical ring-opening polymerization methods. The cationic ring-opening polymerization (CROP) of cyclic disulfides has been documented for decades, and recent studies suggest that this method provides recyclable composites or adhesives and ultrahigh molecular-weight (UHMW) polymers in the megadalton range. Tsarevsky and co-workers^101^ investigated in detail the kinetics and thermodynamic aspects of the rROP of lipoates. They showed in particular that an equilibrium is formed between the propagating radicals and the monomer, characterized by a ceiling temperature of 139 °C. The maximum conversion (about 75%) was achieved when V-70 was utilized as the initiator at 40 °C. Thiyl radical–disulfide exchange events that occur during polymerization and accelerate at elevated temperatures can significantly influence the resulting molecular weight distribution. The presence of a monomer–polymer equilibrium causes the polymer to degrade rapidly when heated to 150 °C or even at lower temperatures, particularly in the presence of radical initiators. Recent work has leveraged these insights to develop RAFT conditions for polymerizing ethyl lipoate homopolymer using a trithiocarbonate end-group that is well-controlled, stable during storage, and triggered by light for on-demand depolymerization.^102^ Moreover, the polymer easily degrades in the presence of reducing agents (such as tributyl phosphine), which cleave the disulfide backbone bonds into thiol functionalities.^101^

According to these features, the use of lipoate derivatives in VAT 3D printing is particularly relevant. Dove and co-workers^81^ thus developed a new photopolymer resin consisting of a mixture of monofunctional menthyl lipoate and difunctional isosorbide lipoate. This resin can be 3D-printed into high-resolution part, degraded, and chemically recycled to be subsequently reprinted. This application will be further discussed in the Applications section.

## Reactivity of Monomers in rROP

The copolymerization kinetics between an rROP monomer and vinyl monomers determines the homogeneity of the distribution of degradable bonds along the main polymer chain. To optimize degradation and minimize the average size of the resulting fragments, it is essential that these bonds are evenly distributed throughout the vinyl polymer structure. Any significant variation in their distribution leads to the formation of long sequences of nondegradable vinyl polymer, which greatly reduces the overall biodegradation potential. The study of copolymerization kinetics relies on the analysis of reactivity ratios (Figure 21). These are defined by the coefficients *r*_v_ (reactivity ratio of the vinyl monomer) and *r*_c_ (reactivity ratio of the cleavable monomer or comonomer). The *r*_v_ parameter corresponds to the ratio between the propagation rate constants of a growing vinyl chain adding either to a vinyl monomer (*k*_vv_) or to a cleavable rROP monomer (*k*_vc_). Similarly, *r*_c_ represents the ratio of the propagation rate constants of a growing cleavable rROP chain adding either to another cleavable rROP monomer (*k*_cc_) or to a vinyl monomer (*k*_cv_).

**Figure 21:**
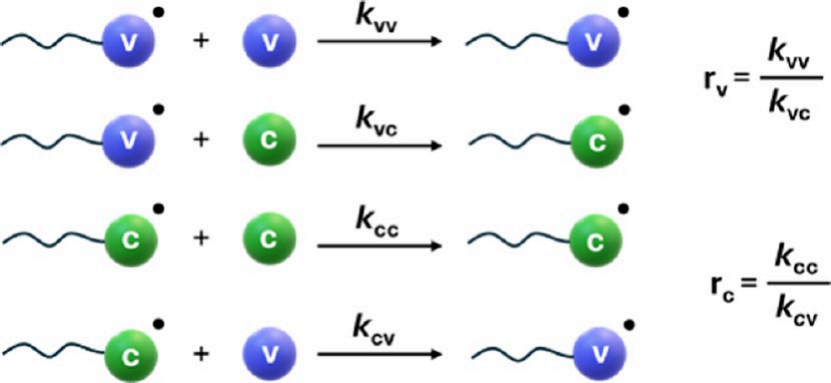
Copolymerization kinetics and associated reactivity ratios.

When the values of *r*_c_ and *r*_v_ are close to 1, all four propagation rate constants of the comonomer pairs are similar. In this case, copolymerization occurs in a random manner, ensuring a homogeneous distribution of the monomer units within the polymer chain. On the other hand, *r*_c_ ≫ 1 and *r*_v_ ≪ 1, or vice versa, *r*_c_ ≪ 1 and *r*_v_ ≫ 1, signals more preferential incorporation of one of the monomers into the polymer. In an uncontrolled polymerization, this inequality in the rate of incorporation of comonomers leads to a nonhomogeneous distribution of degradable bonds along each polymer chain that initiates, grows, and terminates within a short period of time, e.g., ∼1 ms. Furthermore, over time, the preferential incorporation of one monomer leads to a concentration depletion that causes compositional drift, meaning chains that initiate, grow, and terminate at later reaction times have a different average sequence distribution than those formed earlier in the polymerization. Both effects impact the decrease in *M*_n_ observed after subsequent degradation. It is crucial to emphasize that such effects differ with a controlled radical mechanism. Because ideally all chains initiate simultaneously and grow throughout the polymerization, compositional drift in this type of polymerization leads to the formation of polymers with each chain having similar compositional gradients. Thanks to advances in modeling, computer simulations now allow for more precise characterization of polymers. Among the methods used, Monte Carlo-type kinetic simulation (kMC), based on a statistical probabilistic approach, stands out as a particularly effective tool. It not only allows for the precise modeling of the location of monomer units within a polymer chain but also represents a set of polymer chains, thereby faithfully reflecting the material’s structure. A stretched-format visualization is possible, where each line corresponds to a polymer chain, and each colored point symbolizes a monomer unit according to a predefined color code. By having the exact location of the monomers, it becomes possible to theoretically anticipate their degradation and study the evolution of the molecular weight distribution before and after degradation.

Guillaneuf, Lefay, and D’hooge^103^ et al. thus used this technique to investigate the impact of the reactivity ratios and the polymerization mode. They showed first that the polymerization mode does not impact the molar mass distribution of the degraded oligomers (Figure 22).

**Figure 22:**
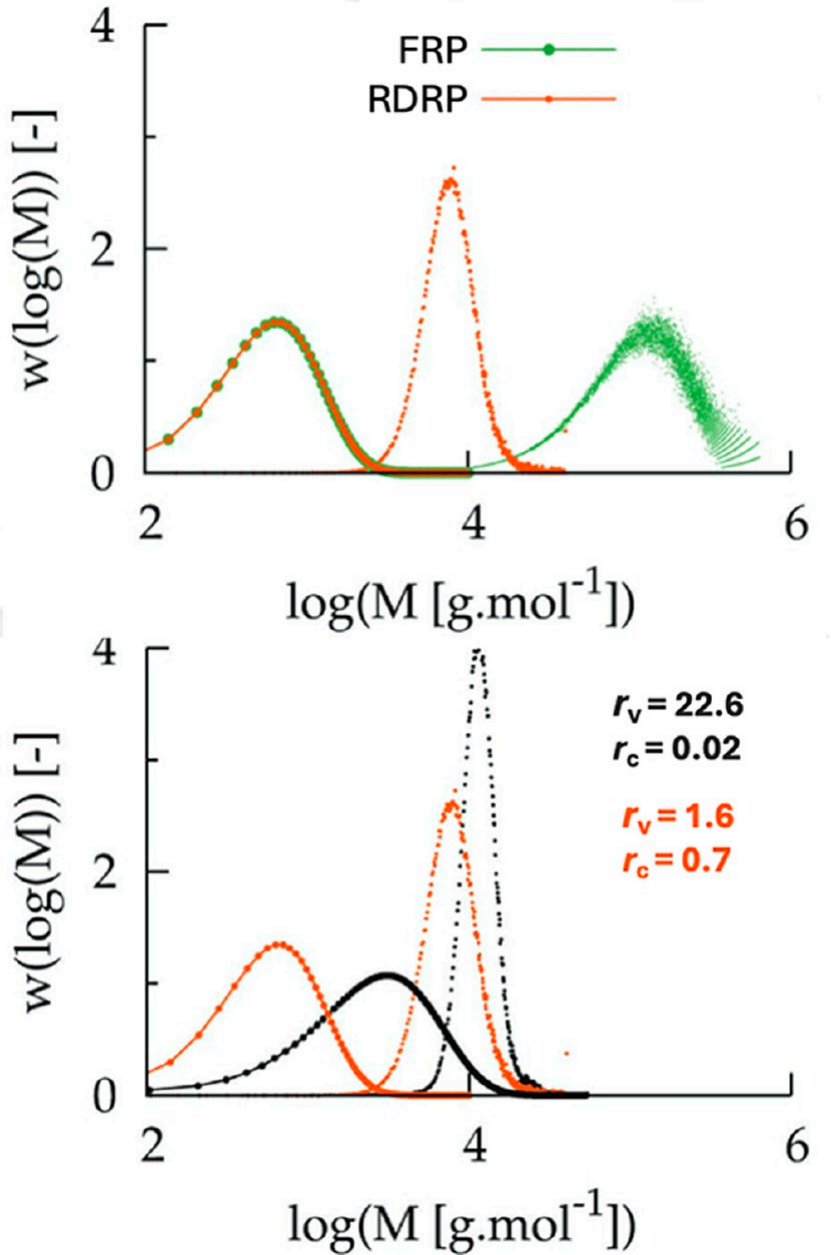
Simulation of individual chain degradation, obtained from kinetic Monte Carlo simulations: Calculated size exclusion chromatography (SEC) traces for polymer chains and degradation products. SEC traces before and after copolymer hydrolysis under various conditions: (top) RDRP (orange) versus uncontrolled (green) radical polymerization. (Bottom) Comparison between the most heterogeneous (black) and most homogeneous (orange) RDRP degradation products. In each panel, the degraded product appears to the left of the corresponding initial polymer (same color). Reproduced from ref 103 with permission. Copyright 2018 Wiley-VCH.

Second, they highlighted the impact of such reactivity ratios by comparing the theoretical degraded oligomers (degree of polymerization after degradation of 7 or 124) obtained from the copolymerization of MDO with either styrene (*r*_c_ ≪ 1 and *r*_v_ ≫ 1) or vinyl ethers (*r*_c_ close to 1 and *r*_v_ close to 1) giving a copolymer with a similar ratio of cleavable bonds (20%) (Figure 22).

Van Herk et al.^104^ compared the kMC simulation method for assessing oligomer length postdegradation with a more accessible analytical solution that facilitates the determination of sequence length distribution using specific equations. They demonstrated that this method offers a direct means of determining the length of small oligomers. The analytical solution neglects the effects of termination occurring during polymerization. To consider the termination event, the authors proposed a correction, namely the probability of chain propagation. By including the additional term, they demonstrated that both the kinetic Monte Carlo simulations and the analytical solutions exhibited strong consistency, regardless of the comonomer pair with moderate to high disparities in reactivity ratios, as well as the composition of the initial monomer feed.^104^

Johnson and co-workers^105^ used a similar Monte Carlo approach to establish broad/universal reactivity-deconstructability relationships, linking reactivity ratios over 4 orders of magnitude to the *M*_n_ decrease between the starting polymer and degraded oligomers as well as the dispersity of the degraded oligomers. They established maps describing such parameters depending on the two reactivity ratios (Figure 23). Considering a high targeted DP of 1,000, they found that the length of the degraded oligomers is dependent on the polymerization mechanism when *r*_v_ < 1 due to compositional drift leading to chains lacking cleavable monomers at the end of an uncontrolled polymerization, whereas only a long fragment lacks cleavable monomers in a gradient-like chain obtained by RDRP.^105^

**Figure 23:**
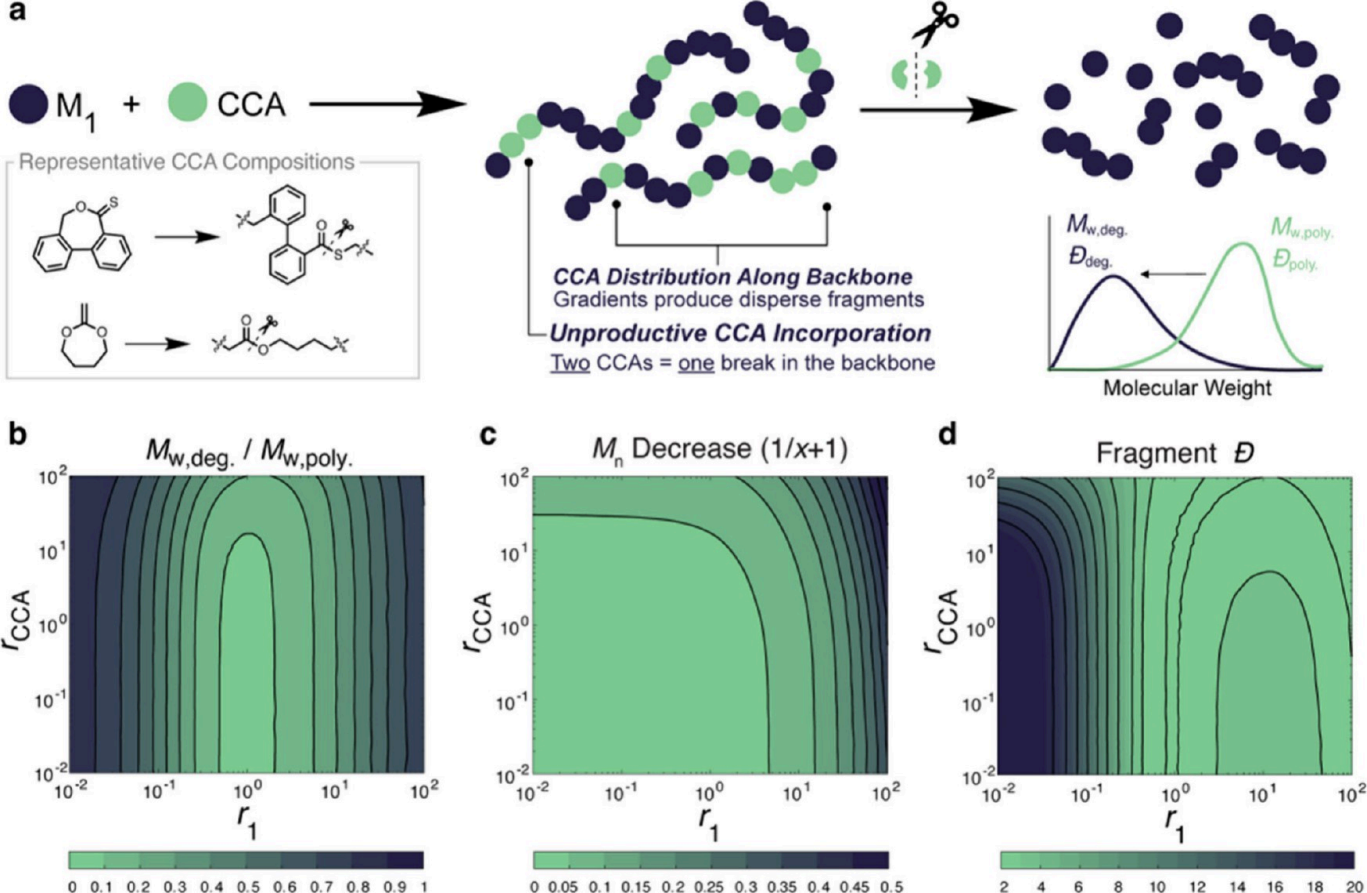
(a) Schematic of the cleavable comonomer additive (CCA) approach for deconstructable copolymers. CCAs copolymerize with standard monomers (“M1”), introducing cleavable sites along the backbone. (b) Relative decrease in molecular weight (*M*_w,deg_/*M*_w,poly_) as a function of reactivity ratio pairs, *r*_1_ and *r*_CCA_, for M1 and CCA, respectively. For all simulations presented, a degree of polymerization of 1000 was targeted with a CCA loading of 2.5 mol %. (c) Fractional decrease in number-average molecular weight (*M*_n,deg_/*M*_n,poly_) as a function of reactivity ratio pairs. (d) Dispersity (*Đ*) of the deconstructed fragments as a function of reactivity ratio pairs. Reproduced from ref 105 with permission. Copyright 2024 American Chemical Society.

They also established the same maps considering cleavable cyclic monomers that exhibit reversible propagation under equilibrium (sulfide cyclic methacrylates and cyclic allyl sulfides for example) or when degradation occurred via a specific sequence (disulfide reduction in the case of lipoates for example). A comparison between the predicted *M*_n_ of degraded oligomers and the experimental values was shown, confirming their established relationships across across different types of monomers, different polymerization mechanisms, etc.^105^

Recent theoretical efforts have built on this foundation by developing efficient simulation tools for handling reversible copolymerizations using deterministic models that are more than an order of magnitude faster than kinetic Monte Carlo while retaining key information about molecular weight and sequence distributions.^106^

Junkers and co-workers^107^ reported a new method utilizing continuous Bayesian optimization of monomer feed in semibatch copolymerization, addressing the composition drift in copolymerization caused by differing reactivity ratios. This method necessitates online monitoring of the reaction and does not require prior kinetic knowledge or modeling of the polymerizations. This method was demonstrated for MMA, BA, and S copolymerized with BMDO, producing copolymers with final *F*_vinyl_ = 0.3, 05, and 0.7 via comonomer feed regulation.

Recent theoretical studies have allowed for the evaluation of thionolactone monomer reactivity in copolymerization with vinyl monomers (such as styrene or acrylates) by analyzing the rate constants governing radical polymerization via ring-opening using DFT calculations.^69,79^ This process includes the addition of propagating radicals to the C=S bond (*k*_add_), the reverse addition, which releases the initial radical and the starting thionolactone (*k*_–add_), and β-scission of the radical intermediate (*k*_beta_), which leads to the incorporation of the thioester bond into the polymer chain and the release of a new propagating alkyl radical (Figure 24a).

**Figure 24:**
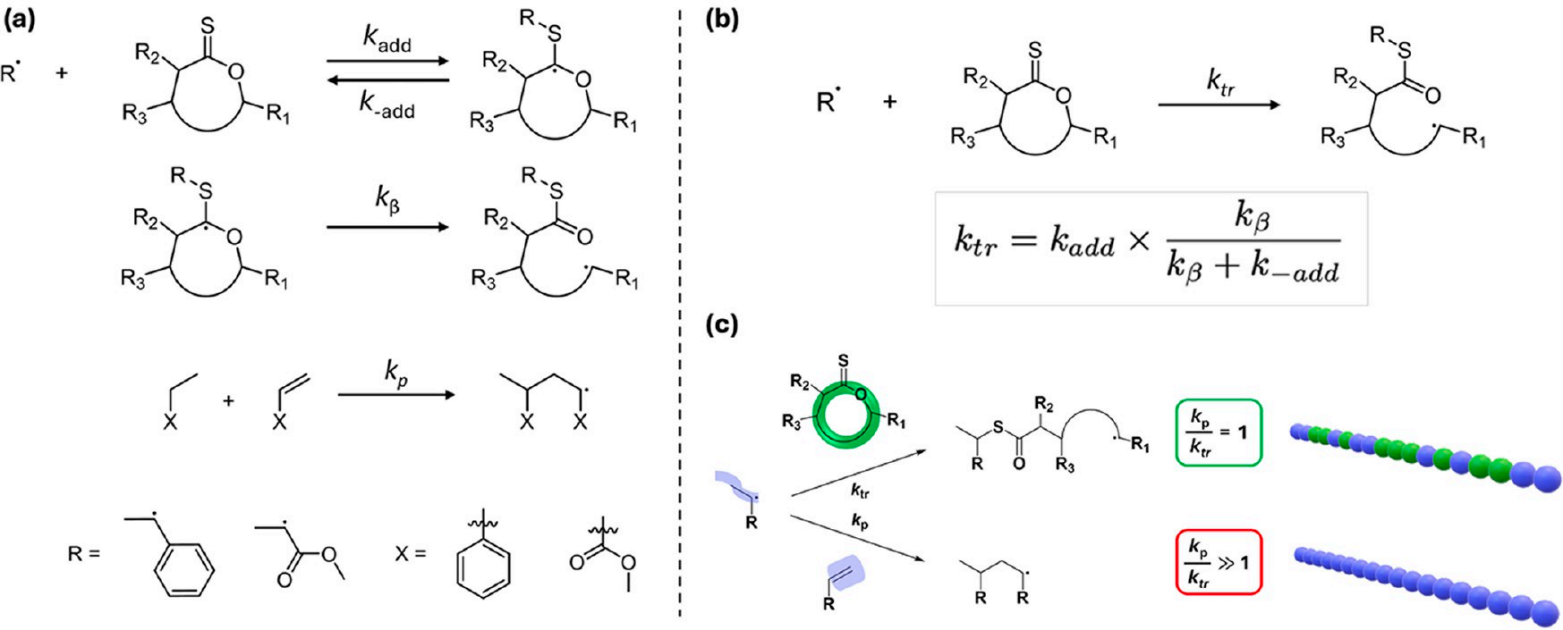
(a) Elementary steps involved in the ring-opening polymerization (ROP) of thionolactones with vinyl monomers, along with the corresponding rate constants: *k*_add_: rate constant for addition, *k*_–add_ rate constant for reverse addition, *k*_β_: rate constant for fragmentation, *k*_p_: rate constant for propagation. (b) Definition of the transfer constant *k*_tr_. (c) Determination of the *k*_p_/*k*_tr_ ratio to estimate copolymerization behavior. Adapted from ref 69 with permission. Copyright 2023 American Chemical Society. To analyze this process, the transfer rate constant (*k*_tr_) was used. This encompasses the three previously mentioned steps and allows for modeling of the addition–fragmentation mechanism. It was then compared to the propagation constant of the vinyl monomer (*k*_p_), providing a relevant criterion to assess the reactivity of the comonomer pair by determining the reactivity ratio *r*_v_. In these systems, the cyclic monomer is typically introduced as an additive in low concentration (less than 10 mol %), meaning that the majority of the growing macroradicals are polyvinyl macroradical. This approach simplified the calculations by avoiding the determination of reactivity ratios specific to thionolactones.

Therefore, comparing the ratio *k*_p_/*k*_tr_ allows for the evaluation of monomer reactivity. If this ratio is ≫ 1, vinyl polymerization predominates. However, when *k*_p_/*k*_tr_ is ≈ 1, it indicates a comparable reactivity between the vinyl monomer and the thionolactone, favoring a random insertion of the thionolactones into the polymer chain (Figure 24b).^69,79^ Another approach based on DFT has recently been proposed by Johnson’s team to design functionalized thionolactones, overcoming a major obstacle to their use: their copolymerization with methacrylates.^70^ Rather than relying on a composite rate constant encompassing the entire radical addition mechanism, this study used DFT calculations to analyze in detail the energetic profile of this reaction and identify the key transition states influencing the reactivity ratio.^70^ Furthermore, this approach enables direct computation of the energetic barrier associated with cross-propagation of copolymers with thionolactones, mitigating inherent reliance on polyvinyl macroradical reactivity values as a reference point.

This approach, combining theoretical and experimental chemistry, represents an interesting strategy, both to optimize the design of monomers by modulating their reactivity ratios with targeted comonomers and to study their structural compatibility to improve the degradation of polymers formed by rROP.

## Synthesis of Copolymers in rROP

In the following section, we have chosen to focus on vinyl monomers rather than cyclic monomers; accordingly, for a selected vinyl-based system, we will compare the various available polymerization chemistries, and at the end of each vinyl monomer family subsection, we present comparative tables summarizing the different polymerization conditions in an effort to rank the most promising approaches, considering solely the polymerization performance and conditions, while degradation aspects will be addressed in the subsequent section.

### Poly­(Styrene)

#### Cyclic Ketene Acetal (CKA)

Owing to its exceptional durability, facile processability, and hydrolytic stability, polystyrene (PS) is extensively used in the packaging, insulation, construction, and food processing sectors. However, its widespread use and stability against degradation also make it a significant contaminant of soils, rivers, lakes, and oceans. Since the 1980s, several studies have focused on the copolymerization of styrene with CKA, but the reported results are often contradictory, especially concerning the incorporation of CKA and the degradability of polystyrene. In 1982, Bailey’s group described the equimolar copolymerization of 2-methylene-1,3-dioxepane (MDO) and styrene (S), resulting in a poly­(MDO-*co*-S) containing 23.4 mol % of MDO.^26^ In the same year, they obtained a degradable poly­(BMDO-*co*-S) with 31.1 mol % of 5,6-benzo-2-methylene-1,3-dioxepane (BMDO), using bulk polymerization at 120 °C for 24 to 36 h with 1 to 3 mol % of di-*tert*-butyl peroxide (DTBP).^108^

However, later work from the same group revealed that a copolymerization carried out with a molar ratio of 80:20 in favor of MDO contained only 10 mol % of MDO in the final polymer, highlighting the very different reactivity ratios of the monomers.^109^ Van Herk and Thoniyot^110^ subsequently identified an error in the ^1^H NMR characterization, repeated in several studies, leading to a misinterpretation of the results on the degradability of CKA-based polystyrene. They demonstrated that the signals associated with the esters depended heavily on the triads present in the copolymer, thus explaining some of the discrepancies observed in the literature, emphasizing the importance of accurate copolymer characterization (Figure 25). Although purified samples seemed to show high ester incorporation, the majority of polystyrene contained very few CKA units.

**Figure 25:**
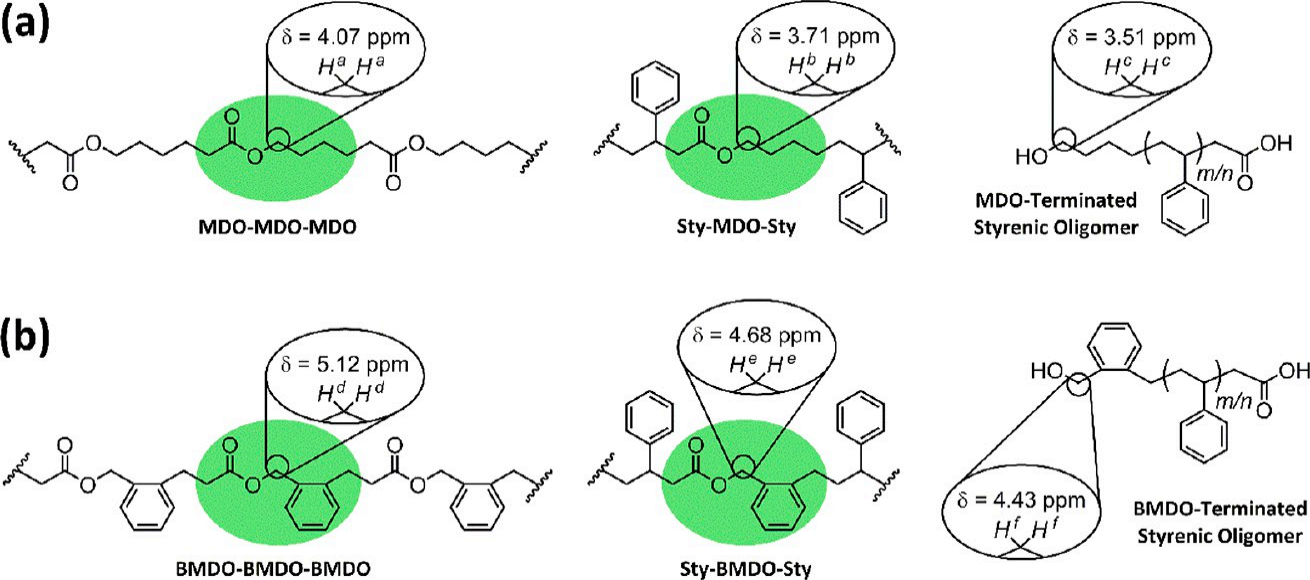
(a, b) Important protons used for the ^1^H NMR (CDCl_3_) analysis of P­(CKA-*co*-S) copolymers and degraded styrenic oligomers in P­(MDO-*co*-S) copolymers and P­(BMDO-*co*-S) copolymers. Reprinted from ref 110 with permission. Copyright 2020 MDPI.

The work of Van Herk and Thoniyot^110^ confirmed that, under Bailey’s conditions, an initial molar ratio of MDO:S = 80:20 resulted in a polymer containing only 12 mol % of MDO. Additionally, an equimolar MDO-S copolymerization resulted in only 2% MDO in the final copolymer, a value similar to an equimolar BMDO-S copolymerization that also only contained 2% BMDO.

These results show significant compositional drift during the copolymerization of styrene and CKA, in agreement with the reactivity ratios obtained by Bailey in his later research (*r*_MDO_ = 0.021 and *r*_S_ = 22.6 at 120 °C), as well as those published by the Guillaneuf group in 2022 ^111^ and those obtained for BMDO by Wickel and Agarwal^112^ (*r*_BMDO_ = 1.08 and *r*_S_ = 8.53 using ATRP at 120 °C). The best copolymerization and degradation conditions^110^ were achieved with an equimolar MDO/S ratio and 0.1% DTBP at 120 °C for MDO, and an equimolar BMDO/styrene ratio with 0.1% DTBP at 120 °C for BMDO. Regarding MTC, Hiraguri and Tokiwa^113^ reported in 1993 a rROP between styrene and 2-methylene-1,3,6-trioxocane (MTC). They claimed an incorporation of 24 mol % MTC after an equimolar copolymerization carried out at 120 °C for 24 h with 3 mol % DTBP as the radical initiator. However, they likely encountered significant compositional drift. Therefore, although the purified sample might contain 24 mol % CKA, this does not necessarily mean that the incorporation was homogeneous throughout the polymer. Additionally, no molecular weight analysis of the styrenic oligomers after ester hydrolysis was provided.

#### Sulfide Cyclic Methacrylates (SCM)

Copolymers with styrene are achievable, although the low reactivity of the SCM monomer compared to styrene limits the degradation of the chains. The new MCS8 to MCS10 structures, whose polymerization can be controlled by RDRP, have also been copolymerized with styrene. However, compositional drift was observed, with reactivity ratios of *r*_MCS_ = 3.02 and *r*_S_ = 0.35.

#### Thionolactone (TL)

It was initially reported that the copolymerization of DOT with styrene was unsuccessful.^36^ However, Guillaneuf’s group re-examined this reaction in detail and demonstrated that, contrary to previous claims, DOT could be incorporated into the main chain of polystyrene (PS) at 80 °C.^111^ However, its low reactivity limited insertion to about half of the initially introduced amount. In the same work, Guillaneuf’s group showed that copolymerization became more efficient at higher temperatures (150 °C), achieving reactivity ratios of *r*_S_ = 0.55 and *r*_DOT_ = 1.68.^111^ High molar mass polystyrene with properties close to commercial general purpose PS (also called crystal PS) were obtained that could be decomposed to oligomers with *M*_n_ close to 2,500–5,000 g·mol^–1^. At the same time the Johnson group reported similar results.^78^ They also highlighted that the incorporation rate of DOT into polystyrene backbone depended on the solvent used and that DOT derivatives bearing F, OMe, or SPr substituents modified the reactivity (Figure 26) and solubility of DOT-based thionolactones.

**Figure 26:**
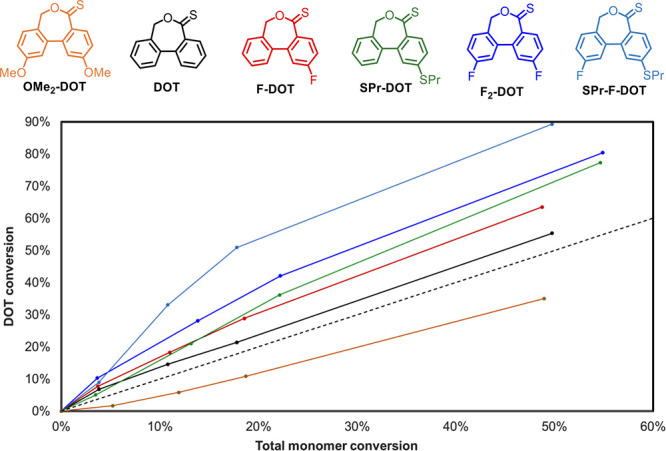
Analysis of substituent effects on the copolymerization of styrene with DOT-based thionolactone. Reproduced from ref 78 with permission. Copyright 2022 American Chemical Society.

Recently, Roth and his collaborators^114^ proposed a new thionolactone structure, 3,3-dimethyl-2,3-dihydro-5*H*-benzo­[e]­[1,4] dioxepine-5-thione (DBT), capable of copolymerizing with styrene. However, this copolymerization is not ideal, as DBT tends to polymerize first, followed by styrene.^114^ Destarac et al.^74^ and Satoh et al.^73^ independently reported the use of 3,6-dimethyl-5-thioxo-1,4-dioxan-2-one (TLD), a biosourced monomer derived from l-lactide. A cross-copolymerization with styrene is possible, but unlike other thionolactones, ring retention was observed in the case of TLD, leading to the simultaneous introduction of thioester and thioacetal linkages in the polymer chain, which is unfavorable for degradation. Free radical copolymerization of TIC, the biosourced thionolactone introduced by the Reineke’s group,^100^ with styrene led to the formation of statistical copolymers where TIC was incorporated in both its open and cyclic forms. A DFT analysis revealed that the opening of TIC’s ring generates an intermediate S,S,O-orthoester structure, stabilizing the propagating radical and creating a stabilized chain end. While this stabilization does not allow for full control as in living polymerization processes, chain extension of p­(TIC) with styrene led to the formation of a block copolymer without the need for a chain transfer agent.^100^

Guillaneuf and his collaborators investigated various thionolactone structures from a DFT theoretical study, and proposed 7-phenylthiapane-2-thione (POT).^69^ The copolymerization of this thionolactone with styrene led to a homogeneous incorporation of degradation sites, with nearly equivalent reactivity ratios for POT and styrene (*r*_S_ = 0.8 and *r*_POT_ = 0.9) at 80 °C (Figure 27). This reaction was also efficiently carried out under RAFT conditions.

**Figure 27:**
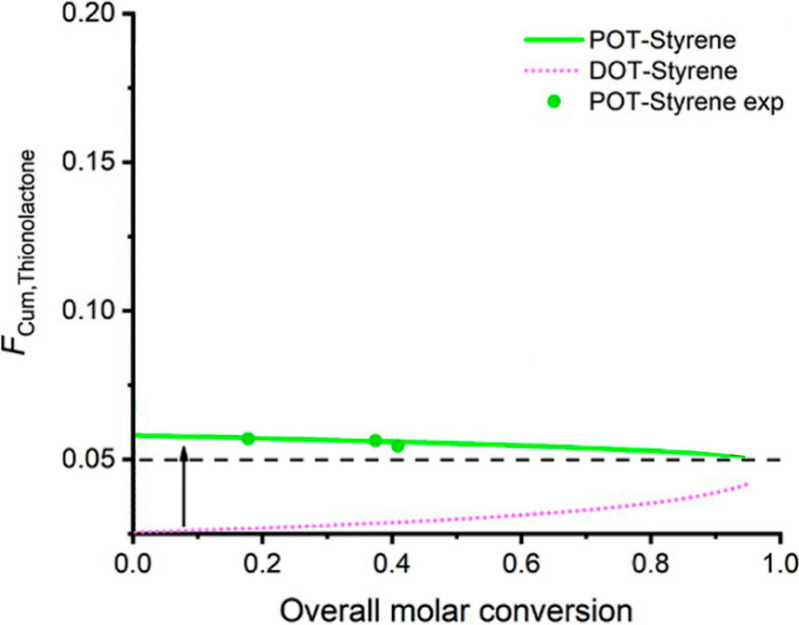
Experimental and theoretical composition of the POT monomer as a function of overall molar conversion during solution copolymerization at 80 °C initiated with 0.5 mol % AIBN from a mixture of POT (5 mol %) and styrene in anisole: Cumulative average molar thioester content in the copolymers. Adapted from ref 69 with permission. Copyright 2023 American Chemical Society.

#### Lipoates (Lp)

Endo and co-workers^48^ were one of the first to explore the copolymerization at 82 °C of lipoamide (LAm) with various vinyl comonomers, including styrene (St), using 2,2′-azobis­(isobutyronitrile) (AIBN) as a thermal initiator. They showed that the incorporation of LAm was lower than the LAm content in the feed (7.8% in the final copolymer with a feed ratio of 15%). Second, they observed that the *M*_n_ of the copolymer was less affected by the incorporation of LAm compared with other vinyl monomer.

They concluded that “the attack of the macro thiyl radical, formed by the reaction of a styryl polymer radical with LAm, on St is not as great a disadvantage as in the other polymerization cases due to the resonance stabilization in the formation of styryl radicals.”^48^

Later Albanese and co-workers investigated controlled radical copolymerization of styrene and lipoic acid and reported that they did not obtain degradable polystyrene containing disulfide bonds.^34^ However, under uncontrolled free radical conditions, styrene does successfully copolymerize with lipoic acid and derivatives such as ethyl lipoate.^115^ For example, a monomer mixture containing 96 mol % styrene and 4 mol % lipoic acid resulted in a p­(αLA-*co*-St) copolymer with a number-average molecular weight (*M*_n_) of 40 kg·mol^–1^ a dispersity (*Đ*) of 1.9, and an incorporated lipoate content of 3.7 mol % that closely matched the feed ratio. Incorporating small amounts of lipoate—i.e., 0.5 to 5 mol %—was sufficient to cause a significant reduction in molecular weight after thermal treatment in DMF while maintaining a glass transition temperature (*T*_g_) between 80 and 85 °C near that of pure polystyrene (∼90 °C).

Note that degradation under these conditions is believed to involve retro thiol–Michael reactions under oxidative conditions that promote the formation of sulfoxide and sulfone intermediates, facilitating the cleavage of not only S–S disulfide bonds but also thioethers, which amplifies the decrease in molecular weight.^115^ Additionally, another team recently demonstrated the possibility of synthesizing an ultrahigh molecular weight styrene copolymer incorporating linear disulfide bonds, starting from *tert*-butyl lipoate (*t*-BLp).^116^ The polymerization was performed in emulsion at 25 °C, initiated by potassium persulfate (KPS). After degradation by dithiothreitol (DTT) at 80 °C or by UV irradiation at 365 nm, these copolymers showed partial reduction in their molecular weight. For instance, a p­(S-*co*-*t*BLp) copolymer with an initial molecular weight of *M*_w_ = 460,000 g·mol^–1^ exhibited reduced molecular weights ranging from *M*_w_ = 90,000 to 70,000 g·mol^–1^ after degradation, although significant fractions of the undegraded polymer remained.

#### Summary

The development of degradable polystyrenes mainly relies on the incorporation of comonomers capable of introducing breaking points into the polymer chain. CKAs were the first to be studied for their ability to introduce hydrolyzable ester units into the polystyrene chain. However, their low reactivity toward styrene results in significant compositional drift, with the actual incorporation often much lower than expected. Even under optimized conditions, the incorporation rates remain modest, limiting their effectiveness in terms of degradability. SCMs present a possibility for incorporation into polystyrene, but their low reactivity compared to styrene limits effectiveness. Regarding thionolactones, significant progress has been made due to their ability to introduce cleavable thioester bonds. While DOT is weakly reactive at moderate temperatures, it becomes more efficient at high temperatures.

More recent structures such as DBT, TLD, or TIC show contrasting behavior, notably due to differences in reactivity or cycle retention. However, POT and some DOT derivatives stand out for their homogeneous incorporation and nearly balanced reactivity ratios with styrene, paving the way for truly degradable polystyrene. Concerning lipoates, they allow the introduction of disulfide bonds into the polystyrene chain. At low concentrations (0.5 to 5 mol %), they offer good thermal degradability in DMF while maintaining the thermomechanical properties of PS.

In conclusion, among all the families studied, thionolactones, particularly POT due to the ease of its synthesis, now appear as the most efficient comonomers for obtaining degradable polystyrene (Table 1). With reactivity ratios close to the ideal with styrene, good incorporation homogeneity, and compatibility with controlled processes such as RAFT, POT combines effectiveness, simplicity of implementation, and high degradation potential. Lipoates also provide interesting alternatives, depending on the intended application.

**Table 1:**
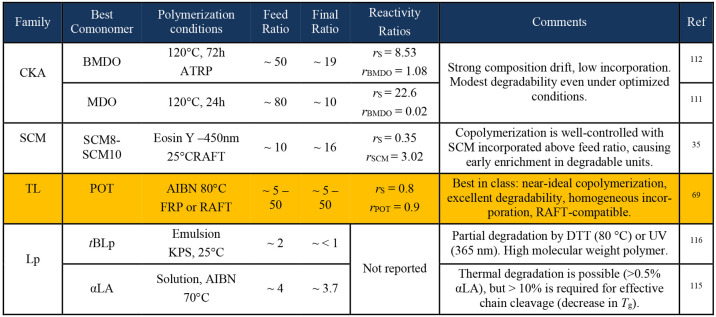
Summary Table of the Best Comonomers for Designing Degradable Polystyrene via rROP

### Polyacrylates/Acrylamides

#### Cyclic Ketene Acetal (CKA)

The majority of the copolymerizations involving CKAs reported in the literature are carried out with acrylic derivatives. In all cases, the quantity of ester function in the polymer backbone is lower than that present in the reaction medium. For example, the copolymerization of MDO (2-methylene-1,3-dioxepane) with methyl acrylate (MA) at 50 °C showed low incorporation of MDO, with only 4 to 18 mol % incorporated from mixtures containing 50 and 85 mol % CKA, respectively.^117^ Moderate incorporation was also observed with MDO copolymerized with propargyl acrylate at 65 °C.^118^

It should be noted, however, that these cases of high incorporation were associated with incomplete ring-opening rates, around 80% for one and 30 to 60% for the other, which may limit the effective degradability of the copolymers. Other copolymerizations, notably with *n*-butyl acrylate (*n*BA) and *N*-isopropylacrylamide (NIPAAm), allowed for higher CKA unit content, up to 20 to 30 mol %, from initial mixtures containing 50 mol % CKA.^119,120^ In agreement with DFT calculations performed by Guillaneuf et al.^82^ that showed a better ring opening with increasing temperature, a seven-membered CKA substituted with methyl groups alpha to the oxygens showed significantly higher reactivity in copolymerization with MA at 110 °C via ATRP, achieving an incorporation rate of 47% from an equimolar initial mixture.^121^ These results are nevertheless consistent with reactivity ratios *r*_c_ < 1 and *r*_v_ > 1. Many reactivity ratios were determined between CKAs and acrylate derivatives that could have more than 1 order of magnitude of difference. Van Herk and co-workers^122^ determined with the same methodology such reactivity ratios between MDO and methyl acrylate, *n*-butyl acrylate, 2-ethylhexyl acrylate and dodecyl acrylate. They observed that alkyl acrylates and MDO copolymerization follows a family-like behavior with 0.2 < *r*_c_ < 0.01 and 4 > *r*_v_ > 1.5.^122^ To address issues related to compositional drift caused by reactivity discrepancies, Van Herk and co-workers^123^ developed a semicontinuous feed strategy. This involves gradually introducing a fraction of the acrylate derivative into a mixture containing MDO and acrylate. This approach helps reduce composition drift, improves CKA conversion, limits monomer losses, and thereby improves the degradability of the formed chains. The resulting copolymers exhibited more homogeneous degradation and produced better-defined oligomers.^123^

#### Sulfide Cyclic Methacrylates (SCM)

The first studies on the original SCM monomers reported that cross-linking occurred during the copolymerization of SCM1 with acrylic derivatives (e.g., methyl acrylate).^31^ The authors therefore developed the SCM2 monomer, containing an additional methyl group to limit cross-linking,^31^ but this monomer was not tested in copolymerization with acrylic derivatives.

The second generation of SCM monomers developed by Niu and co-workers exhibited a different reactivity.^35^ Copolymerization of SCM8 with acrylates revealed a faster incorporation of SCM, leading to a nonuniform distribution of weak bonds along the polymer chain. To address this, Niu et al.^124^ demonstrated that polymerization at room temperature using the PET-RAFT (photo-RDRP) process improved reactivity ratios. Using *fac*-Ir­(ppy)_3_ as the photocatalyst and a trithiocarbonate chain-transfer agent, they successfully produced various acrylate and acrylamide copolymers with reactivity ratios close to 1.^124^ This resulted in low-molecular-weight oligomers (*M*_n_) with reduced dispersity, significantly better than those obtained under the same conditions at 70 °C.^124^

#### Thionolactone (TL)

The first studies on thionolactones revealed that dibenzo­[c,e]-oxepine-5-thione (DOT) was mainly able to copolymerize efficiently with acrylates and acrylamides, thus forming copolymers containing thioester units in the main chain.^36,37^ It is important to note that DOT exhibits a faster incorporation rate than acrylic derivatives, which can lead to compositional drift during polymerization.^37^ DOT was copolymerized with various acrylates, such as methyl acrylate (MA), with reactivity ratios at 80 °C of *r*_MA_ = 0.2 and *r*_DOT_ = 1.6.^125^ It was also copolymerized with other acrylates, such as *n*-butyl acrylate (*n*BA) (Figure 28), *tert*-butyl acrylate (*t*BA), benzyl acrylate (BnA), trifluoroethyl acrylate (TFEA), and PEG acrylate (PEGA), by uncontrolled free radical polymerization (FRP) or by RAFT (Figure 28).^36,37^

**Figure 28:**
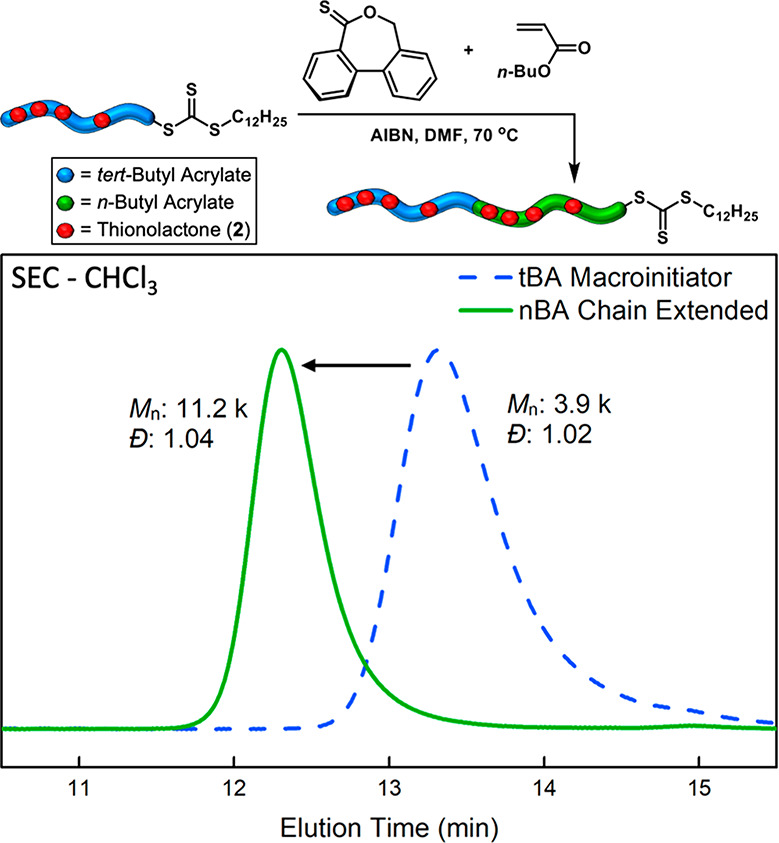
Preparation of a polyacrylate-based P­(*n*BA-*b*-*t*BA) diblock copolymer containing 5 mol % of DOT into the two blocks. Reproduced from ref 37 with permission. Copyright 2019 American Chemical Society.

Successful copolymerizations of DOT with other monomers, such as acrylonitrile (AN) and acrylamides (e.g., *N*-isopropylacrylamide (NIPAm), acrylamide (AAm), *N,N*-dimethylacrylamide (DMAm), *N,N*-diethylacrylamide (DEAm), and *N*-3-(*N*-4-sulfobutyl-*N,N*-dimethylammonium)­propylacrylamide (SBBAm)), have also been reported.^126^ The thionolactone POT was also copolymerized with isobornyl acrylate. A detailed kinetic analysis showed reactivity ratios of *r*_IBA_ = 0.3 and *r*_POT_ = 1.4 for the POT–isobornyl acrylate system (Figure 29).^69^ These copolymerizations were carried out in solution at 80 °C in anisole (50 mol %), with initiation by 0.5 mol % of AIBN. Thanks to these reactivity ratios, a more homogeneous incorporation of thioesters into the polymer chains can be achieved using POT compared to the DOT thionolactone.^69^

**Figure 29:**
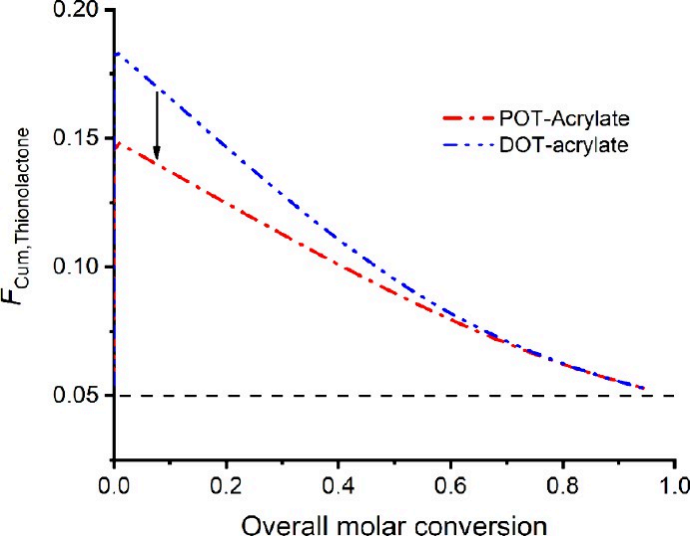
Experimental and theoretical composition of the POT monomer as a function of overall molar conversion during solution copolymerization at 80 °C, initiated with 0.5 mol % AIBN from a mixture of POT (5 mol %) with isobornyl acrylate: Cumulative average molar thioester composition of the copolymers. Adapted from ref 69 with permission. Copyright 2023 American Chemical Society.

The Cu­(I)-catalyzed atom-transfer radical copolymerization of DOT is feasible; however, it is hindered by the dethionation of DOT when exposed to trace amounts of oxygen or water.^127^ This side reaction occurs rapidly and results in the depletion of significant quantities of the thionolactone monomer. Despite the aforementioned issues, degradable copolymers with low dispersities were formed, although with a lower thioester content and without the necessity for strictly anhydrous conditions. Under anhydrous conditions, the formation of lactone could be reduced to at least 5%, though not entirely eliminated.^127^ The photo-rROP of DOT and methyl acrylate was investigated by Matyjaszewski and co-workers^128^ through photoiniferter reversible addition–fragmentation chain transfer (RAFT) polymerization at ambient temperature, without the use of external radical initiators or photocatalysts. Polymers containing thioester linkages in the backbone were synthesized, despite the occurrence of side reactions such as desulfurization-oxygenation and O–S isomerization of DOT, which were promoted by photoexcited thiocarbonyl groups. These polymers exhibited degradation when exposed to amines and bleach.

Similarly to styrene, thionolactide (TLD) undergoes copolymerization with acrylate derivatives, but with a competition between ring-opening and ring-retaining mechanisms.^73,74^

#### Lipoate (Lp)

The copolymerization of α-lipoic acid (αLA) and/or derivatives with various acrylic monomers has been investigated using both conventional^129,130^ and controlled^34^ radical polymerization methods. A notable feature observed is the dynamic reactivity of the disulfide units present in these copolymers, enabling efficient degradation under reducing conditions.^34,129–131^ In his pioneering study, Endo et al.^48^ reported a similar amount of disulfide units as the lipoamide monomer suggesting a similar reactivity of the two monomers and a better copolymerization behavior with the acrylate derivatives than with styrene or methacrylate derivatives.

This was later confirmed by Tsarevsky and co-workers.^33^ Albanese and co-workers^130^ investigated the free radical copolymerization of *n*-butyl acrylate and ethyl lipoate (ELp) at 2 M total monomer concentration using conventional free-radical polymerization at 40 °C with V-65 as a thermal azo-initiator. They found that both monomers copolymerize efficiently with ethyl lipoate incorporating more readily than *n*BA. Second, they observed that diad sequences and thus degradability are significantly influenced by the polymerization reaction conditions employed (Figure 30). Increased absolute monomer concentrations and reducing reaction temperatures led to higher *M*_n_ for the copolymers but also smaller oligomers after degradation. This observation is consistent with a reversible propagation mechanism of the thiyl radical (where the ceiling temperature of the homopolymer is ca. 139 °C). Using an analysis developed by Wittmer that takes into account the reversible monomer propagation of ethyl lipoate yielded an estimate of the reactivity ratios for *n*BA–ELp copolymerization: *r*_ELp_ = 18.5 and *r*_nBA_ = 0.36.^130^ In a following study, Bates, Hawker, and Read de Alaniz extended this type of copolymerization to RAFT^34^ and miniemulsions^129^ that both showed similar monomer reactivities. To further reduce the dithiolane content necessary for good degradability, a more efficient strategy was recently developed that exploits cleaving both S–S and C–S bonds along the backbone (retro-thio-Michael reactions)^115^ at elevated temperatures in a polar solvent (DMF).^115^ For example, the radical copolymerization of *n*BA and ELp (95:5) was carried out at 70 °C in toluene, with AIBN as the thermal initiator. After 2 h, the copolymer was isolated by precipitation, and its molar mass (*M*_n_ = 62 kDa) was consistent with previous studies.^130^ The copolymer composition determined by ^1^H NMR spectroscopy indicated a lipoate incorporation of approximately 6.1 mol %.^115^

**Figure 30:**
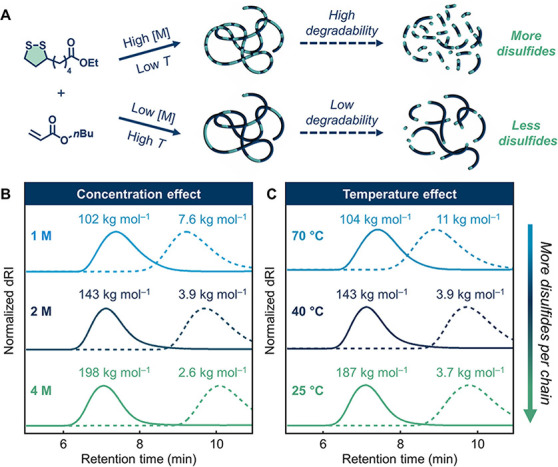
Tunable degradation of poly­(acrylate) copolymers by controlling the concentration and temperature of polymerization. (a) The degradability of lipoic-acid–acrylate copolymers can be synthetically tuned through polymerization conditions that control the average number of disulfide bonds per polymer chain. (b, c) As evidenced by size-exclusion chromatography, (b) higher monomer concentrations ([M]), and (c) lower polymerization temperatures (T) improve degradability. Reproduced from ref 130 with permission. Copyright 2023 American Chemical Society.

The thermolysis of this p­(ELp-*co*-nBA) in a polar solvent such as DMF or NMP at 100 °C in air for 18 h resulted in significant backbone cleavage with the molecular weight decreasing from 62 kg mol^–1^ to 3.2 and 2.2 kg mol^–1^, respectively. The copolymerization of lipoate and acrylate derivatives was then extended to the preparation of antibacterial copolymers by copolymerizing benzyl lipoate with primary amine-containing cationic monomer (*tert*-butyl (2-acrylamidoethyl) carbamate (Boc-AEAm)) and hydrophilic comonomers, including hydroxyethyl acrylamide (HEAm) and poly­(ethylene glycol) methyl ether acrylate (PEGMEA), by RAFT polymerization (see Applications section for more detail).^132^ This system was also extended to polyacrylate networks and 3D printing via either the copolymerization of the lipoate derivative with multiacrylate monomers^133^ or with multilipoate derivatives and monofunctional acrylate monomers.^134,133^

#### Summary

In conclusion (Table 2), cyclic ketene acetals (CKAs), when copolymerized with acrylates, exhibit distinct reactivities, leading to variable incorporation of these units into polymer chains. Experimental conditions, such as temperature and polymerization method, play a crucial role in the efficiency of monomer incorporation. Nevertheless, *f*_CKA,0_ = 20–40 mol % are usually needed to obtain 5–15 mol % ester incorporation into the polymer backbone. Strategies such as semibatch polymerization have been proposed to overcome compositional drift issues, thereby improving both degradability and homogeneity of the resulting copolymers. Although the reactivity of CKAs may limit their complete incorporation, these monomers nevertheless represent a valuable tool for designing degradable polymers when appropriate strategies are employed. Unlike first-generation sulfide cyclic methacrylates (SCM), second-generation SCM (SCM8–SCM10) have shown significant potential for copolymerization with acrylates and acrylamides, particularly due to the ability to control monomer reactivity via reversible deactivation radical polymerization (RDRP) techniques. The introduction of phenyl groups has helped prevent cross-linking and improve copolymer regularity. However, achieving a homogeneous distribution of SCM units along the polymer chain remains a major challenge.

**Table 2:**
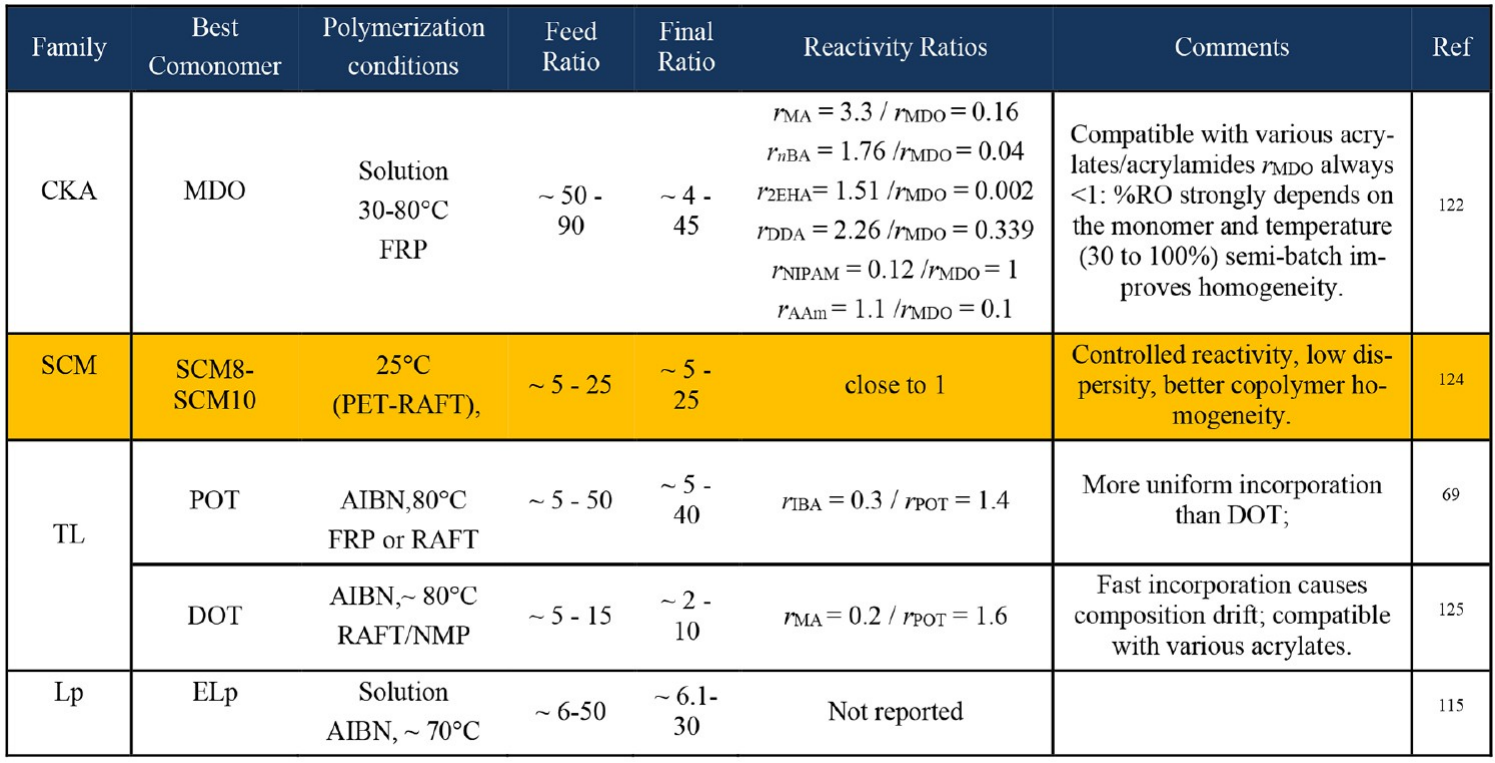
Summary Table of the Best Comonomers for Designing Degradable Polyacrylates/Acrylamides via rROP

Recent advances, such as photo-RDRP at room temperature, have led to more uniform copolymers with improved reactivity ratios, paving the way for degradable materials with more predictable architectures.

Thionolactones, such as DOT and POT, appear as the most promising monomers for copolymerization with acrylates and acrylamides since it is possible to obtain degradable polyacrylates or polyacrylamides using only *f*_thionolactone,0_ = 1–5 mol % as additive. However, there is still a minor composition drift with acrylate and acrylamide derivatives that poses challenges in terms of reactivity and homogeneous incorporation.

α-Lipoic acid (αLA) and its derivatives, such as ethyl lipoate (ELp), are increasingly studied for copolymerization with acrylates. While degradation of dithiolane-based vinylic copolymers previously required high comonomer content, recent studies show that combined cleavage of S–C and S–S bonds enable efficient degradation at as low as 0.4 mol % of lipoic acid or ethyl lipoate. Finally, lipoates stand out for their ease of synthesis; however, the low *T*_g_ of lipoic acid (∼10 °C) is a consideration that may limit certain applications.

### Polymethacrylates

#### Cyclic Ketene Acetal (CKA)

In general, copolymerization of CKAs with methacrylates is more efficient than with other monomers, such as styrene derivatives. However, incorporation often remains limited, typically between 10 and 40% for an initial molar fraction of 50%.^88,135^ The copolymerization of methacrylic esters, particularly methyl methacrylate (MMA), with CKAs (such as MDO, BMDO, or MPDL) has been extensively studied, revealing complex interactions and highly variable reactivity ratios. In general, CKAs are less reactive than methacrylate derivatives, which limits their incorporation into the final polymer unless reaction conditions are carefully optimized.

Nicolas and co-workers^52^ studied the copolymerization of MDPL and MMA at 90 °C in toluene, obtaining reactivity ratios *r*_MDPL_ ≈ 0.01 and *r*_MMA_ ≈ 4.0, indicating very low MDPL reactivity and thus limited incorporation into the copolymer.^52^ Comparative studies on the MDO/MMA pair showed that reactivity ratios vary strongly depending on experimental conditions. In pulsed laser polymerization (PLP) at 40 °C, *r*_MDO_ = 0.057 and *r*_MMA_ = 34,^12,136^ whereas in conventional bulk polymerization at 120 °C, the values changed to *r*_MDO_ = 0.04 and *r*_MMA_ = 3.5.^137^ Van Herk and co-workers revisited some extreme reactivity ratios and showed a family-like behavior with *r*_CKA_ close to 0.05 and *r*_methacrylate_ close to 4. Copolymerization of MDO with methacrylate derivatives, such as oligo­(ethylene glycol) methyl ether methacrylate (OEGMA) or dimethylaminoethyl methacrylate (DMAEMA), proved difficult: only 15–20% of the CKA is incorporated into the final copolymer for an equimolar initial composition (50% CKA).^124,138–143^

Maynard and Sawamoto^144^ showed that the addition of a fluorinated methacrylate promoted ester-unit insertion during the copolymerization of BMDO with PEGMA. Similarly, Ouchi and colleagues^145^ used pentafluorophenyl methacrylate and demonstrated that its copolymerization with BMDO efficiently produced sequences rich in alternation. After polymerization, the pentafluorophenyl group can be easily modified by alcoholysis or aminolysis, allowing access to functional methacrylate- or methacrylamide-type copolymers.^145^

The introduction of controlled radical polymerization techniques (ATRP, RAFT, or NMP) significantly improved CKA incorporation and provided better control over macromolecular architecture,^88^ even if these methods often lead to increased dispersity compared to the same system without CKA, indicating partial loss of control.^135^ For example, in ATRP at 120 °C for 72 h, BMDO incorporation reaches 34% and 53% for initial CKA compositions of 50% and 70%, respectively, with favorable reactivity ratios (*r*_BMDO_ = 0.53; *r*_MMA_ = 1.96).^112^ Similarly, NMP copolymerization of MPDL with MMA produced well-defined copolymers containing more than 20% MPDL.^52^ Moreover, approaches using macroinitiators instead of RDRP have been used to prepare block copolymers, such as PCL-Azo or PEG-Azo in combination with MDO/MMA copolymerization. Depending on experimental conditions (temperature, time, initial concentration), observed incorporation rates range from 10 to 57%.^138,141,146^

The use of sugar-derived CKAs, such as Glu-CKA, has also been investigated in copolymerization with MMA (Figure 31).^57^ Incorporation remains limited due to low conversion. However, adding a third monomer, *N*-phenyl maleimide, significantly improves insertion thanks to its high reactivity toward CKAs.

**Figure 31:**
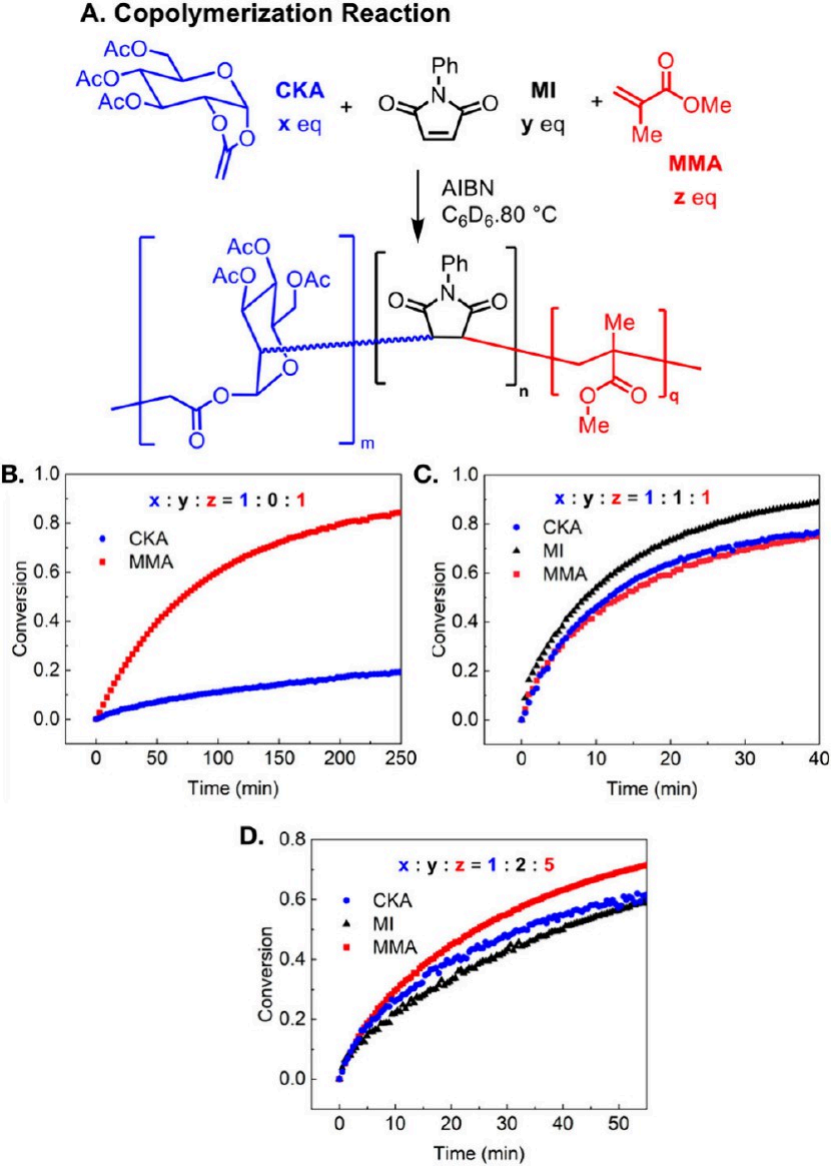
Real-time ^1^H NMR monitoring of copolymerization. (A) Reaction scheme; (B–D) real-time ^1^H NMR tracking of conversion versus reaction time: (B) Glu-CKA/MMA = 1:1; (C) Glu-CKA/MI/MMA = 1:1:1; and (D) Glu-CKA/MI/MMA = 1:2:5. Reproduced from ref 57 with permission. Copyright 2024 American Chemical Society.

Terpolymerizations thus showed similar conversion rates for all three monomers, leading to well-defined terpolymers.^57^ A similar approach was also performed by Sardon and co-workers using a crotonate ester as an additive.^147^

Overall, these studies confirm that the copolymerization of CKAs with methacrylic esters is a viable strategy for introducing degradable ester units into vinyl polymers. BMDO stands out as one of the best candidates in terms of compatibility and incorporation, whereas other CKAs, such as MDO or MPDL, require more careful adjustments. The polymerization technique, choice of comonomers, experimental conditions (temperature, duration, type of initiator), as well as the potential presence of reactive functional groups strongly influence the outcomes. Finally, optimizing polymerization conditions and judicious monomer selection appear essential to maximize CKA incorporation while maintaining satisfactory control over the macromolecular structure.

#### Sulfide Cyclic Methacrylate (SCM)

Initial research revealed that the first-generation monomers allowed for the formation of copolymers with methacrylate derivatives. More specifically, the incorporation of SCM monomers into poly­(methacrylate)-type structures occurs randomly, suggesting similar reactivity ratios.^31^ In a subsequent study, Hawker et al.^41^ expanded their investigations to cyclic monomers bearing ester, thioester, and disulfide groups in the side chain (SCM5–SCM7), which were also capable of copolymerizing with methacrylic monomers such as methyl methacrylate (MMA), 2-hydroxyethyl methacrylate (HEMA), and 2-dimethylaminoethyl methacrylate (DMAEMA).^41,148^ Hydroxypropyl methacrylate (HPMA) was also copolymerized with SCM7 to prepare degradable amphiphilic diblock copolymer nano-objects.^149^ Finally, for the new structures SCM8 to SCM10 developed by Niu et al.^35^ whose polymerization can be controlled via RDRP, copolymerization with methyl methacrylate at room temperature results in composition drift, with the following reactivity ratios: *r*_SCM_ = 0.18; *r*_MMA_ = 5.81.^124,150^

#### Thionolactone (TL)

Thionolactones (TL) had not been effectively copolymerized with methacrylates until recently, mainly due to unfavorable reactivity, resulting in either negligible conversion or significant composition gradients in the resulting polymers.^114^ DOT was shown to be only a spectator during the MMA copolymerization.^36,37^ The copolymerization of DBT with two methacrylates poly­(ethylene glycol) methyl ether methacrylate and *tert*-butyl methacrylate (*t*BuMA) was explored.^114^ Although copolymerization occurred, the difference in reactivity between DBT and the methacrylate derivatives led to a high composition drift, with DBT being incorporated at the chain end, thereby limiting copolymer degradation.^114^

A major breakthrough was achieved by the Johnson group, who developed benzylic-functionalized DOTs (bDOTs), leading to a tertiary radical after ring opening to overcome this limitation.^70^ Using density functional theory (DFT) calculations, the team studied the full energy landscape of the radical addition process to identify key transition states (Figure 32A). These analyses showed that introducing radical-stabilizing substituents on the benzylic carbon of DOT could lower the transition-state energy, thereby facilitating ring opening and enabling successful copolymerization with methyl methacrylate (MMA) (Figure 32B).^70^

**Figure 32:**
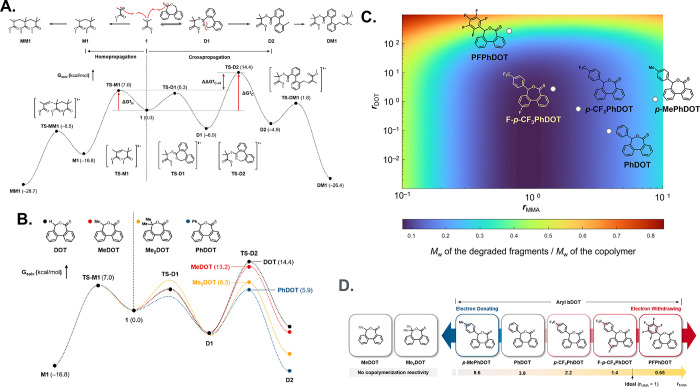
(A) Relative Gibbs free energy profile for an MMA radical reacting either with MMA or with DOT, calculated to model homopropagation and cross-propagation of a chain terminating in MMA. (B) The effect of benzylic substituent(s) on the energy profile was evaluated using DFT calculations. Calculations were performed at the wB97X-D3/def2-SVP level of theory; electronic energies of all optimized structures were re-evaluated using wB97X-D3/def2-TZVP/CPCM (toluene). (C) A Monte Carlo simulation evaluates the efficiency of aromatic bDOTs as cleavable comonomers. The heat map generated by the simulation visualizes the ratio of degraded fragment size to copolymer size as a function of reactivity ratios, for a 2.5% molar loading of CC in copolymers with DP 1000. (D) A series of bDOTs was synthesized for optimization of copolymerization reactivity. Reproduced from ref 70 with permission. Copyright 2024 American Chemical Society.

Experimental results confirmed that this modification effectively controlled copolymerization reactivity with MMA, allowing the targeted incorporation of degradable units along the polymer chain (Figure 32C,D). Using experimentally determined reactivity ratios, Monte Carlo simulations were then employed to determine the optimal bDOT for minimizing *M*_W_ of cleaved fragments. This advance led to the development of the first thionolactone effective for methacrylates (F-p-CF_3_PhDOT), with optimized reactivity ratios: *r*_MMA_ = 1.4 and *r*_F‑p‑CF_3_PhDOT_ = 2.9. This innovation opens new prospects for integrating thionolactones into methacrylic polymers, offering more precise control over degradation and the final material properties.^70^

A new PMMA degradation strategy was developed by the Guillaneuf group,^125^ based on the incorporation of the thionolactone DOT. Rather than derivatizing DOT for optimized reactivity, this approach introduced an auxiliary monomer that could copolymerize with both DOT and MMA.

Thus, to ensure effective integration of the thioester units, an auxiliary monomer such as MA or PhMal is required. The most promising results were obtained with the PhMal-based terpolymerization Figure 33).^125^ Using Monte Carlo simulation, it was possible to both demonstrate the production of triads containing DOT units in the PMMA backbone and identify synthesis conditions leading to MMA-rich polymers capable of controlled degradation by hydrolysis (Figure 33). This degradation results in a significant decrease in molar mass (up to 25-fold), yielding oligomer-like segments (Figure 33).^125^ The experimental conditions were extended to other methacrylate derivatives. Lastly, the triad PhMal-DOT-PhMal was shown to be instable at high temperature (above 170 °C), leading to a new decomposition trigger.

**Figure 33:**
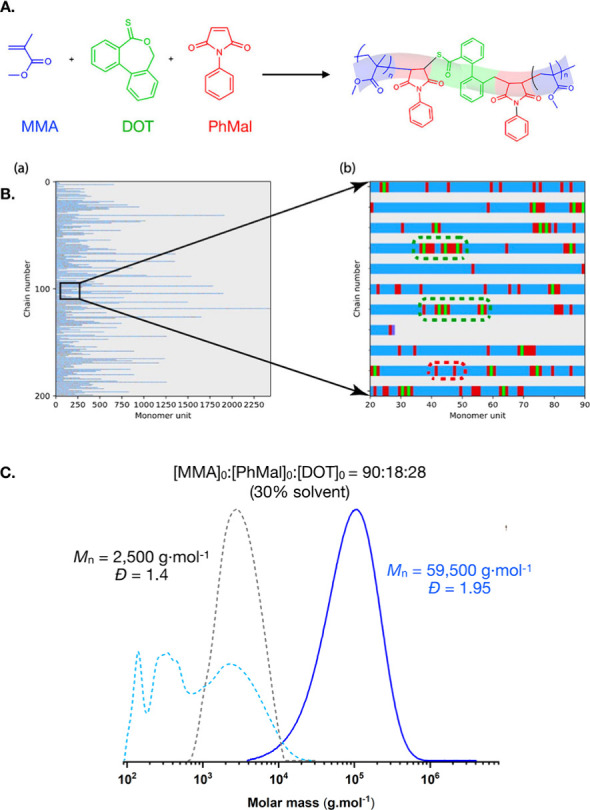
(A) Preparation of degradable PMMA derivatives via terpolymerization of MMA, DOT, and *N*-phenylmaleimide (PhMal; in red). (B) Simulated monomer sequences for modeling-assisted copolymerization with [MMA]_0_:[PhMal]_0_:[DOT]_0_ = 90:18:28 (30% solvent). Monomer sequences follow the color code from panel (C). On the right: selection of chains from the left panel, showing isolated MMA-PhMal units (red box) and MMA-DOT-PhMal triads (green box). Reproduced from ref 125 with permission. Copyright 2025 Springer Nature.

#### Lipoate (Lp)

Endo first studied the copolymerization of lipoamide (LAm) with methyl methacrylate (MMA) using 2,2′-azobis­(isobutyronitrile) (AIBN) as a thermal initiator.^48^ They found that MMA did not copolymerize with LAm, attributing this lack of reactivity to the steric hindrance of MMA–LAm dyads.^48^ Another example is the copolymerization of lipoic acid, which was performed in a controlled manner via the RAFT method with MMA. However, due to the incompatibility between the radical stability of lipoic acid and MMA, this led to homopolymerization of MMA.^34^ Copolymerization with methacrylates remains a challenge, with very recent efforts beginning to investigate light-mediated dithiolane-ene as a possible mechanism to promote at least some reactivity.^151^

#### Summary

In conclusion (Table 3), CKAs offer the possibility of copolymerizing with methacrylates, enabling the design of potentially biodegradable materials. However, these monomers are generally less reactive than conventional methacrylates, requiring adjustments to polymerization conditions. Furthermore, CKA incorporation rates can vary depending on the polymerization method used. In contrast, SCM monomers present certain advantages, notably their ability to copolymerize with methacrylates in an almost random manner due to similar reactivity ratios. This monomer family also provides multiple degradation pathways, which are useful for designing biodegradable polymers. However, in controlled polymerizations, composition drift can occur, limiting their effectiveness in some cases.

**Table 3:**
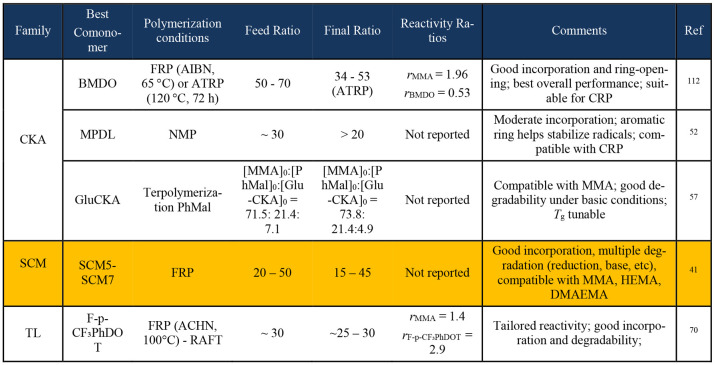
Summary Table of the Best Comonomers for Designing Degradable Poly­(methacrylic ester) via rROP

Although early attempts at thionolactone copolymerization showed suboptimal reactivity ratios, significant progress has been made with the development of benzyl-functionalized thionolactones. These modifications improved thionolactone reactivity, facilitating their incorporation into methacrylic polymers and providing better control over material degradation.

Finally, although lipoates have been explored, the radical stability of lipoic acid proves incompatible with methacrylates. This incompatibility leads to MMA homopolymerization, limiting the utility of lipoates for producing degradable methacrylate-based polymers at this time.

Thus, among the different monomer families, SCM and benzyl-functionalized thionolactones (bDOTs) stand out as the most promising options for rendering methacrylic polymers degradable. Their optimized reactivity and ability to precisely control material degradation make them preferred choices. While CKAs also offer advantages, they often require more complex adjustments and stricter control of polymerization conditions to be fully effective.

### Nonstabilized Monomers

#### Cyclic Ketene Acetal (CKA)

Among the less activated monomers, vinyl acetate (VAc) has been the most extensively studied, particularly via uncontrolled free radical polymerization (FRP)^152,153^ and RAFT polymerization.^154,155^ The first studies on the copolymerization of VAc with cyclic ketene acetals (CKA), such as MDO and MPDL, date back to 1982 with the work of Bailey and colleagues.^26,156^ Subsequently, Hiracuri and co-workers^113^ studied the copolymerization of VAc with larger-ring CKAs (8-membered), achieving high incorporation rates (up to 40 mol % for a 50/50 mol % initial mixture). Agarwal et al.^152^ and Albertsson et al.^153^ confirmed the efficient incorporation of MDO in random copolymers with VAc, as evidenced by multiple NMR techniques. Electrophilic radicals are often important for promoting radical addition to nucleophilic olefins. Unexpectedly, vinyl acetate (VAc), characterized by a nucleophilic propagating radical, produces the most advantageous copolymerization outcomes with CKAs. Frontier molecular orbitals (FMO) were initially examined as they often offer a comprehensive insight into the interactions occurring during such reactions.^157^ The HOMO and LUMO orbital energies of MDO were determined to be of higher energy to those of MA, VAc, and methyl vinyl ether, a highly nucleophilic monomer, hence affirming the pronounced nucleophilicity of MDO. As anticipated, the 1-methoxycarbonyl-ethyl radical (EEst) exhibits greater electrophilicity than the 2-acetyl-2-propyl radical (EAc). However, the principal interaction with MDO in both instances occurs between the HOMO of the MDO double bond and the β-SOMO, indicating that both radicals function as weak electrophilic radicals toward MDO, irrespective of the electrophilic nature of the adding radical.^157^ MDO may thus be regarded as a hyper nucleophilic monomer in comparison to conventional vinyl monomers. This may elucidate the less favorable copolymerization of MDO with MA in contrast to its copolymerization with VAc.

All experimental results show that VAc copolymerization with various CKAs leads to good incorporation of ester units. For example, MDO copolymerized with VAc under thermal conditions (DTBP, 120 °C, 12 h) reaches an ester content of 49% in the copolymer for a 1:1 initial mixture.^26^ MPDL, copolymerized with VAc under similar conditions, reaches 40% ester units.^156^ Conversely, at lower temperatures (AIBN, 50 °C), these percentage decrease (34% for MDO), illustrating the influence of polymerization conditions on incorporation efficiency.^26^

Furthermore, several functional VAc derivatives have been explored. Vinyl chloroacetate (VClAc) copolymerized with MDO produced a degradable poly­(vinyl acetate) (F_MDO_ = 0.05) capable of inhibiting ice recrystallization.^158^ Vinyl bromobutanoate (VBr) copolymerized with MDO via RAFT yielded postpolymerization modifiable copolymers (azidation, CuAAC cycloaddition).^159^ Additionally, poly­(ethylene glycol)-based VAc oligomers, such as ethylene glycol methyl ether vinyl acetate (MeOVAc) and tri­(ethylene glycol) methyl ether vinyl acetate (MeO_3_VAc), were copolymerized with MDO via RAFT, giving degradable, thermosensitive, and noncytotoxic copolymers suitable for protein bioconjugation applications.^159^

Photoinduced controlled radical polymerization (CMRP) has also been applied to MDO/VAc copolymerization at room temperature, allowing the synthesis of well-defined copolymers despite unfavorable reactivity ratios (*r*_VAc_ = 1.89; *r*_MDO_ = 0.14).^160^

However, the limited structural diversity of VAc derivatives restricts copolymer functionality, prompting the exploration of other comonomer families. Among these, phosphonated monomers, such as vinyl dimethylphosphonate, showed good reactivity with CKAs, with incorporation rates ranging from 36 to 60 mol % for initial mixtures between 50 and 75 mol %.^161^ In contrast, vinylphosphonic acid, due to steric hindrance and competition with cationic polymerization, led to lower incorporation (16 to 32 mol % for initial mixtures between 50 and 72 mol %).^162^

Other CKA-based copolymers have been obtained with monomers such as *N*-vinylpyrrolidone (NVP).^163,164^ Comparisons between bulk polymerization and supercritical CO_2_ polymerization with MTC showed that bulk polymerization favored better CKA incorporation.^165,166^ *N*-Vinylacetamide was also reported to be successfully copolymerized with MDO.^167^

To overcome the limitations imposed by VAc, vinyl ethers (VE) have proven to be very interesting comonomers.^157^ They exhibit more favorable reactivity ratios with CKAs (*r*_VE‑butyl_ = 1.61; *r*_MDO_ = 0.73), enabling the synthesis of P­(MDO-*co*-VE) copolymers with very high CKA content (*F*_CKA_ ∼ 0.95) and near-quantitative ring-opening.^157^ Copolymerizations between MDO and the 2-chloroethyl vinyl ether (CEVE) also enabled the production of easily postfunctionalizable copolymers via nucleophilic substitution, azidation, or CuAAC, yielding materials with tunable properties (e.g., solubility, PEG functionalization, colloidal stability, cytocompatibility).^157^ MDO and MTC were copolymerized with various vinyl ethers bearing for example PEG-chains or sugar units to prepare degradable nanoparticles for applications in drug delivery.^168–170^

#### Thionolactone (TL)

Destarac et al.^72,171^ and Guillaneuf et al.^71^ have shown, for example and simultaneously, that ε-thionocaprolactone (TCL) and decathionolactone (TDL) can be copolymerized with vinyl acetate and its derivatives. However, both authors observed a higher incorporation rate of thionolactones compared to vinyl esters, leading to compositional drift during polymerization.^71,72,171^

In this copolymerization process, the presence of unopen units was reported in bulk copolymerization,^72^ whereas only open units were observed when polymerization was carried out in solution.^71^ RAFT-controlled copolymerizations using a xanthate agent (XA1) were also conducted with TCL and either vinyl acetate (VAc),^71^ or vinyl pivalate (VP) at 70 °C.^72^ Kinetic monitoring showed that TCL was consumed faster than the vinyl esters, producing polymers with good molar mass control (*M*_n_ ≈ 6 kg/mol) and a dispersity decrease with conversion from 2.7 to 1.5. NMR analysis revealed that TCL was mostly incorporated in its open form (degradable thioester bond), although a notable fraction remained in the closed cyclic form (close to 40%).^72^ A nonrandom organization, with a predominance of TCL–TCL dyads, suggests the formation of transient dimeric complexes, explaining the slow polymerization and difficulty of polymerizing TCL alone.

Copolymerization of thionolactide (TLD) with VP yielded low-molar-mass copolymers (*M*_n_ ≈ 1000 g·mol^–1^). TLD consumption was much slower than that of VP, indicating high reactivity of vinyl ester radicals toward TLD. Overall conversion remained limited (20% after 20 h), suggesting that radicals formed after addition to TLD poorly propagate polymerization, leading to premature termination.^74^

A new family of cyclic thionocarbamates (CTC) was developed for copolymerization with *N*-vinyl monomers,^172^ such as *N*-vinylpyrrolidone (NVP), via radical ring-opening polymerization (rROP) at 60 °C using AIBN (Figure 34).^172^ Among the six synthesized CTCs, the MeCTC monomer showed high reactivity (conversion ≥ 98%) with a copolymer molar mass of 13.6 kg/mol, whereas the more sterically hindered tBuCTC exhibited lower incorporation (51%) but higher molar mass (43.2 kg·mol^–1^), indicating more spaced incorporation.^172^

**Figure 34:**
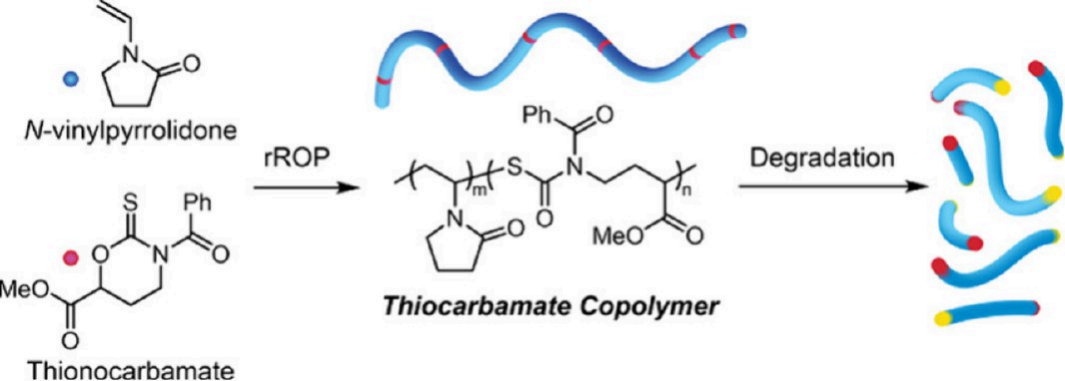
Radical ring-opening copolymerization of cyclic thionocarbamates with *N*-vinylpyrrolidone. Reproduced from ref 172 with permission. Copyright 2024 American Chemical Society.

PhCTC was identified as the optimal candidate, combining good conversion (88%), air stability, and homogeneous incorporation, leading to a uniform statistical architecture. ^13^C NMR analysis confirmed complete ring opening without cyclic retention, which is essential for introducing degradable bonds. The nature of aromatic substituents influenced reactivity: an electron-donating group (p-MeO) slowed polymerization, while an electron-withdrawing group (p-CF_3_) accelerated it. PhCTC was also copolymerized with other *N*-vinyl monomers such as *N*-vinylcarbazole and *N*-vinylcaprolactam. Controlled polymerization attempts using RAFT agents resulted in low molar masses and limited conversions, likely due to interactions between RAFT pyridyl groups and the hionocarbamates.^172^

The copolymerization of DOT with other less activated vinyl monomers has also been reported, notably with PVS (phenyl vinylsulfide), DEVP (diethyl vinylphosphonate), and PVSO (phenyl vinylsulfone), carried out by RAFT or FRP.^173^ The latter shows an incorporation of DOT that is always higher than the vinyl monomer but that is strongly dependent on the nature of the comonomer.

With PVS-DOT, incorporation remains moderate (5 to ∼34% for initial feed ratios of 2.5 to 20%), while it is much higher with DEVP (∼89%) and PVSO (58–72%) for an initial feed of 50%. RAFT allows for better control over the composition and architecture of the copolymers, particularly for PVS-DOT systems, where gradient copolymers with tunable thioester content can be obtained. Increasing the DOT fraction enhances degradability and influences the molecular weight. Finally, postpolymerization oxidation of a PVS-DOT copolymer yields an analogous PVSO-DOT copolymer that retains its degradability.^173^ The specific case of ethylene copolymerization is discussed in detail in a following section.

#### Summary

Studies on the copolymerization of less activated monomers, particularly vinyl acetate (VAc) and its derivatives, have demonstrated efficient incorporation of cyclic monomers (CKAs) such as MDO and MPDL, enabling degradable copolymers with significant ester unit content. Exploration of functional VAc derivatives (VClAc, VBr, MeOnVAc) and the use of controlled polymerizations (RAFT, CMRP) enabled the production of tunable copolymers suitable for biomedical applications. However, the limited diversity of VAc prompted the exploration of new comonomers such as vinyl ethers (VE) and phosphonated monomers, creating the potential for higher incorporation and postpolymerization functionalization.

Simultaneously, the use of thionolactones (TCL, TLD) and cyclic thionocarbamates (CTC) enabled the introduction of degradable bonds into copolymers, with control over architecture and molar mass. Semicontinuous strategies and adjustments of polymerization conditions improved homogeneity and conversion, leading to degradable materials (Table 4).

**Table 4:**
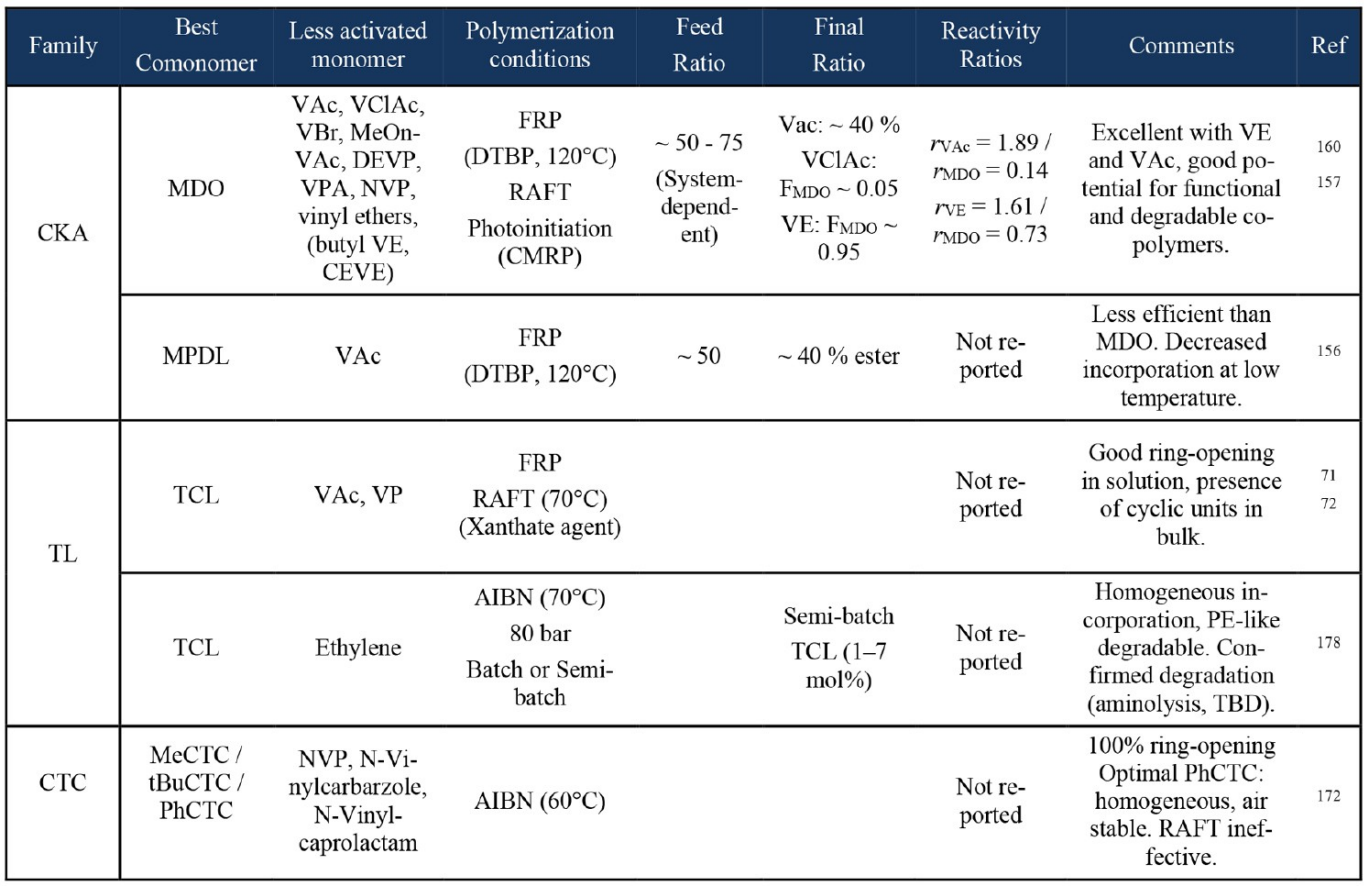
Summary Table of the Best Comonomers for Designing Degradable Copolymers with Less-Activated Monomers via rROP

The particular case of ethylene, the least activated monomer of the less activated monomers, and its copolymerization with CKA or thionolactones will be discussed later in the applications part.

### Maleic Anhydride and Maleimides

Maleic anhydride (MAnH) and maleimides (MI) are extensively used as electron-accepting monomers in alternating copolymerization. The two conjugated carbonyl groups render the alkene in MAnH and maleimides electron-deficient and sterically hindered. Consequently, their homopolymerization and homopropagation are limited, whereas the propensity for cross-propagation is heightened during copolymerization with electron-rich monomers such as styrene and vinyl acetate. Since it could be interesting to prepare alternating degradable materials, the copolymerization behavior of maleic anhydride (MAnH), maleimides, and derivatives has been evaluated in the presence of CKA and thionolactones.

#### Cyclic Ketene Acetal (CKA)

Agarwal et al.^174^ initially documented the radical copolymerization of MDO and *N*-phenyl maleimide (NPhMI). At 60 °C, an identical incorporation of the two monomers was determined regardless of the feed ratio, indicative of alternating copolymerization. Nevertheless, the MDO only led to ketal units. An increase of the temperature improved the insertion of MDO into a combination of ketal and ester units. Sumerlin et al. expanded the CKA polymer library via the copolymerization of MPDL^175^ and BMDO^176^ and maleimides featuring various *N*-substituent groups validating their alternating sequence through comprehensive mass spectrometry analysis.^176^ In that case, they obtained exclusively the expected alternating structure of the ring-opened ester and maleimide units. They attributed this feature to a moderate electron disparity between maleimide and MPDL or BMDO compared to a higher electron disparity in the case of MDO, following the approach developed by Hall and co-workers.^177^ The copolymerization was also compatible with RAFT polymerization and the formation of diblock copolymers to prepare degradable nanoparticles. The same authors tried to extend this work to maleic anhydride (MAnH), but a spontaneous exothermic decomposition of the CKA was observed.^175^ Hiraguri and colleagues^179^ reported the copolymerization of MTC and MAnH between 60 and 120 °C.

Regrettably, this structure was not fully characterized, but it seems that the amount of ester units increased from 60 to 120 °C, since a degradation investigation indicated incomplete degradation of the material, which serves as indirect evidence of the existence of unreacted CKA units. The discrepancy between these results for MAnH copolymerization led Coughlin et al.^180^ to investigate this system in more detail (both BMDO and MTC copolymerized with maleimide, dimethyl maleate, MAnH and itaconic anhydride) both experimentally and using DFT calculations. The observed sensitivity for CKA degradation in the case of anhydride was ascribed to the energy characteristics of the reaction and activation, as well as the presence of impurities. Compared to maleimides and dimethyl maleate, the comparatively lower reaction enthalpies and Gibbs free energies associated with reactions involving maleic or itaconic anhydrides promote the creation of a charge-separated complex with CKA, which may react rapidly in subsequent reactions with water. Following the purification of anhydride monomers by sublimation, CKA decomposition was mitigated, allowing for a successful radical copolymerization between CKA and maleic or itaconic anhydrides.^180^ The anhydride-containing CKA copolymers were postpolymerization functionalized by the selective reaction between the anhydride and 1-propanol, yielding carboxylic acid-containing polyesters.

#### Thionolactone

Roth and co-workers^97^ extended these ideas by copolymerizing electron-deficient olefins such as *N*-alkyl maleimides with thionolactones. In their study they first reported that maleic anhydride, fumaronitrile, and diethyl fumarate do not copolymerize with DOT. Unlike these three comonomers, functionalized *N*-alkyl maleimides do undergo an alternating copolymerization with DOT under both conventional and RAFT mediated polymerizations. The measured reactivity ratio values for the DOT–(*N*-methyl maleimide) MeMI system, *r*_DOT_ = 0.162 and *r*_MeMI_ = 0.654, suggest a propensity for both comonomers to create alternating sequences, albeit with non-negligible MeMI homopropagation events (Figure 35).

**Figure 35:**
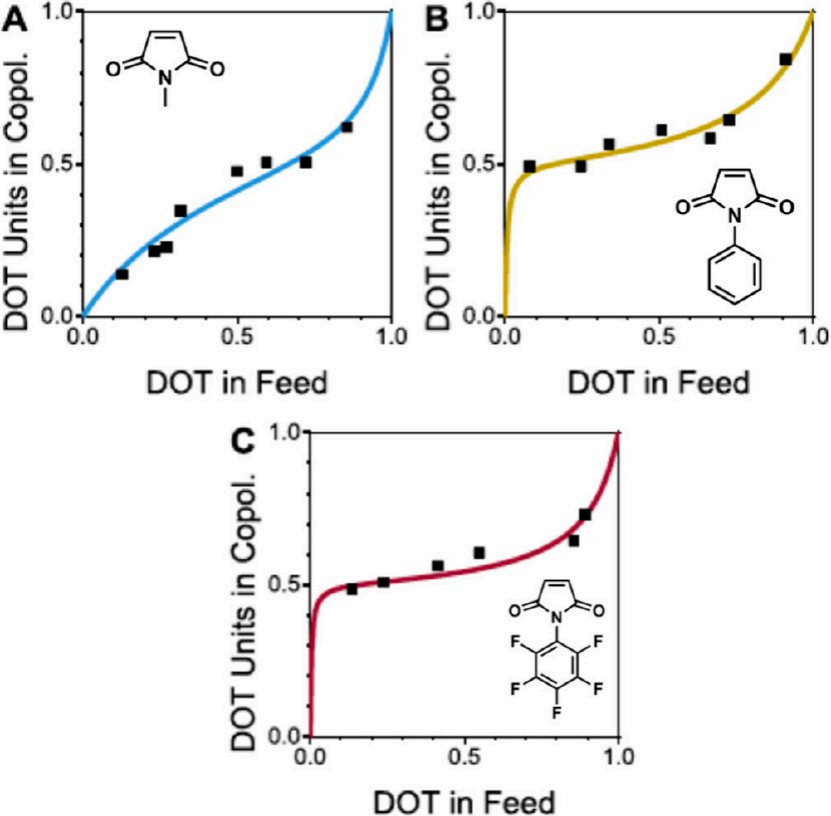
Molar DOT content in maleimide copolymer vs molar DOT fraction in the monomer feed with nonlinear least-squares fitted curves for (A) *N*-methylmaleimide, (B) *N*-phenylmaleimide, and (C) *N*-2,3,4,5,6-pentafluorophenylmaleimide. Adapted from ref 97 with permission. Copyright 2020 American Chemical Society.

Aqueous emulsion polymerization is arguably the most prevalent. The conditions were distinct for the copolymerization of *N*-phenyl maleimide (PhMI) and *N*-2,3,4,5,6-pentafluorophenylmaleimide P­(FPMI). For the DOT–PhMI system, *r*_DOT_ = 0.348 and *r*_PhMI_ = 0.0136, while *r*_DOT_ = 0.198 and *r*_PFPMI_ = 0.0078 were obtained for the combination of DOT with PFPMI (Figure 35). The reduced values were assumed to be due to the enhanced steric and electronic effects of the *N*-aryl group. The thioester functionality was then degraded via aminolysis, and various degradation products were obtained containing between one to two DOT and maleimide units with or without aminolysis of the maleimide function.

## Applications

### Degradable Latexes

Latexes are colloidal systems composed of polymer particles dispersed in a continuous phase. They are typically produced via radical polymerization carried out in heterogeneous media, most commonly through emulsion, miniemulsion, dispersion, or suspension processes.

method for latex synthesis.^181^ The latter find use in several industrial applications, including paints, coatings, medicine delivery systems, cosmetics, and adhesives.

#### Cyclic Ketene Acetals

Preparing (bio)­degradable latexes from CKAs using aqueous polymerization in dispersed media is challenging due to the significant sensitivity of CKAs to aqueous environments, resulting in fast hydrolysis. This point will be discussed in more detail in the hydrosoluble polymer section. This hydrolytic sensitivity is probably the reason why the first works dealing with the use of CKA in aqueous dispersed media were conducted in miniemulsion. This process is designed to protect the monomers from the aqueous phase by trapping them within nanometric droplets, where polymerization should ideally occur exclusively.^182,183^ Miniemulsion copolymerization of BMDO with either MMA or styrene was described in 2012.^184^ Polymer particles were obtained, and the formation of copolymers based on BMDO and MMA or styrene was claimed, while the degradation was only evaluated for the styrene-based particles. In addition, the known sensitivity of BMDO to protic species^185^ did not appear to be an impediment to employ a broad range of surfactants and miniemulsion polymerization conditions. Nevertheless, similar experiments proved difficult to reproduce by other researchers.^50^

Emulsion polymerization offers a versatile and industrially relevant approach to polymerization in aqueous dispersed media. Initiation takes place in water, requiring the hydrophobic comonomers to be slightly soluble in water and to diffuse from large micrometric monomer droplets to the nucleated nanometric particles. The challenge for CKA copolymerization with aqueous emulsion lies in finding suitable conditions to avoid CKA hydrolysis while controlling ester function insertion to ensure homogeneous degradation. Copolymerization of BMDO with MMA presents favorable reactivity ratios (*r*_BMDO_ = 0.53 and *r*_MMA_ = 1.96),^186^ and *ab initio* aqueous emulsion copolymerization of BMDO with MMA was performed for the first time in 2023 by D’Agosto, Lansalot, and collaborators (Figure 36).^187^ Under otherwise conventional conditions, stable latexes made of P­(MMA-*co*-BMDO) copolymers were obtained in a simple and fast process in water, outperforming previously published studies on solution or bulk copolymerization. The reaction was nevertheless only successful in basic media due to faster BMDO hydrolysis under acidic conditions.

**Figure 36:**
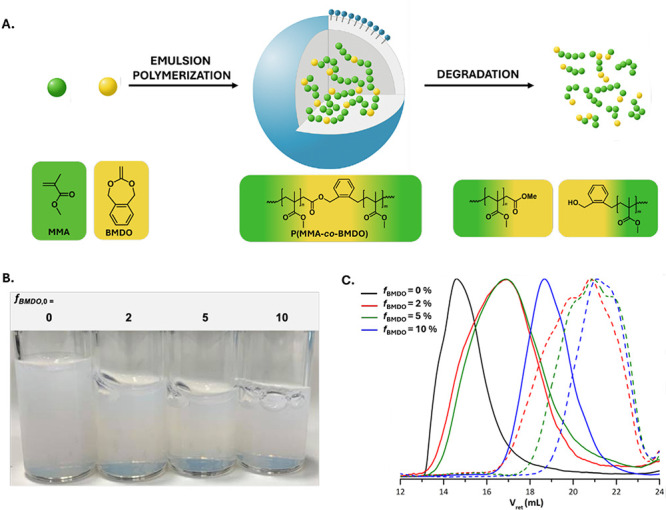
(A) Synthesis of SDS-stabilized P­(MMA-*co*-BMDO) latexes by aqueous emulsion polymerization. (B) Photos of the latexes obtained with various BMDO contents. (C) SEC traces of the dry extracts of P­(MMA-*co*-BMDO) latexes (plain lines) and their degradation products (dashed lines) as a function of incorporated BMDO content. Adapted from ref 187 with permission. Copyright 2023 Royal Society of Chemistry.

Accelerated degradation (using KOH in THF/MeOH) resulted in a loss of at least 80% molar mass for every BMDO-containing copolymer produced.

Recently, the copolymerization of VAc with MDO was studied in emulsion by Carter and his team.^188,189^ Indeed, as mentioned above, MDO was successfully copolymerized with VAc in homogeneous medium (determined reactivity ratios: *r*_MDO_ = 0.47 and *r*_VAc_ = 1.53)^152^ leading to degradable polymers. P­(VAc-*co*-MDO) copolymers were obtained in semibatch emulsion conditions at 40 °C using redox initiators. The key to the success of this synthesis lies in the very specific formulation used to rapidly nucleate particles and thus facilitate the quick escape of MDO from the aqueous phase. Here again, reproducibility was questioned incriminating the difficulty in preventing MDO hydrolysis even when tuning the pH.^190^ It was indeed shown that very fine adjustments of pH, feeding rates, and temperature control during the process, together with the addition of hydrophilic charged comonomers, were required to achieve the targeted degradable P­(VAc-*co*-MDO) chains, with ca. 90% of MDO incorporation.^191^ These latexes were coated onto commercial paper, and the films obtained showed excellent oil and grease resistance as compared to nondegradable compositions, while being partially degradable under basic conditions.^188^

Wenzel, Aguirre, and Leiza studied the seeded semibatch emulsion copolymerization of acrylates with MDO.^192^ They show that the incorporation of MDO under its open form was elusive since hydrolysis of MDO was faster than copolymerization. However, here again, under optimized conditions, i.e., a high feed rate (60 min) and low polymerization temperature (20 °C), 86% of MDO units were inserted, however via ring retention, which does not provide degradable polymer chains. The nature of the acrylate (*n*-butyl acrylate vs *n*-octyl acrylate) was also investigated. With a view of designing original pressure sensitive adhesives (PSA), Movafagh et al. tried to terpolymerize MDO, BA, and VAc in emulsion.^193^ The same combined issues encountered when VAc or BA were copolymerized in emulsion with MDO were reported. Nevertheless, performing the emulsion terpolymerization at 50 °C under slightly basic conditions and with optimized solids content allowed an even distribution of MDO units along the chains, thus maintaining, as claimed in the literature, their hydrolysis at an acceptable level, even under batch conditions. However, the authors did not study the degradation of the obtained latex or the formed PSA.

Thoniyot and his collaborators managed to introduce up to 9 mol % of MDO in the chains during the batch emulsion copolymerization of MDO with methacrylates and/or acrylates conducted at high pH (>10).^194^ The authors had to resort to the use of a neutral surfactant as MDO was not incorporated when charged ones were used. Again, a precise tuning of the emulsion polymerization conditions was required, including the additional use of a hydrophilic noncharged comonomer (2-hydroxyethyl acrylate) to improve the stabilization of the forming particles. The reasoning behind the success of these copolymerizations involving comonomers with disparate reactivities is that hydrolysis is minimized by keeping the charge density on the particles as low as possible. According to the authors, this would favor monomer transport by collisions reducing the chance of hydrolysis.

All the previous works dealt with polymerization in aqueous dispersed media. Latex particles incorporating CKA units have also been obtained in organic solvent using dispersion polymerization,^195,196^ a process where the ingredients are soluble in the dispersing phase while the forming polymer is not. Copolymerization of CKA in dispersion exclusively involves the combination of rROP with polymerization-induced self-assembly (PISA), a technique that relies on the chain extension of a solvophilic polymer obtained via RDRP with a solvophobic block, thereby leading to the self-assembly of the resulting block copolymers into nanoparticles. PISA can potentially work for any controlled (radical) polymerization techniques but has been mostly developed in RAFT-mediated systems.^197^ Some of the latexes obtained by rROPISA were transferred in water when possible. Nicolas’s group^196^ demonstrated that a poly­(lauryl methacrylate) macromolecular chain transfer agent (macroRAFT) could be used in rROPISA in heptane to prepare degradable nanoparticles via copolymerization of benzyl methacrylate with MDO,^195^ MPDL,^196^ (Figure 37) or BMDO.^196^ Nanoparticles with a degradable shell were also obtained by mediating rROPISA of benzyl methacrylate with a poly­(lauryl methacrylate-*co*-BMDO) macroRAFT.^195^

**Figure 37:**
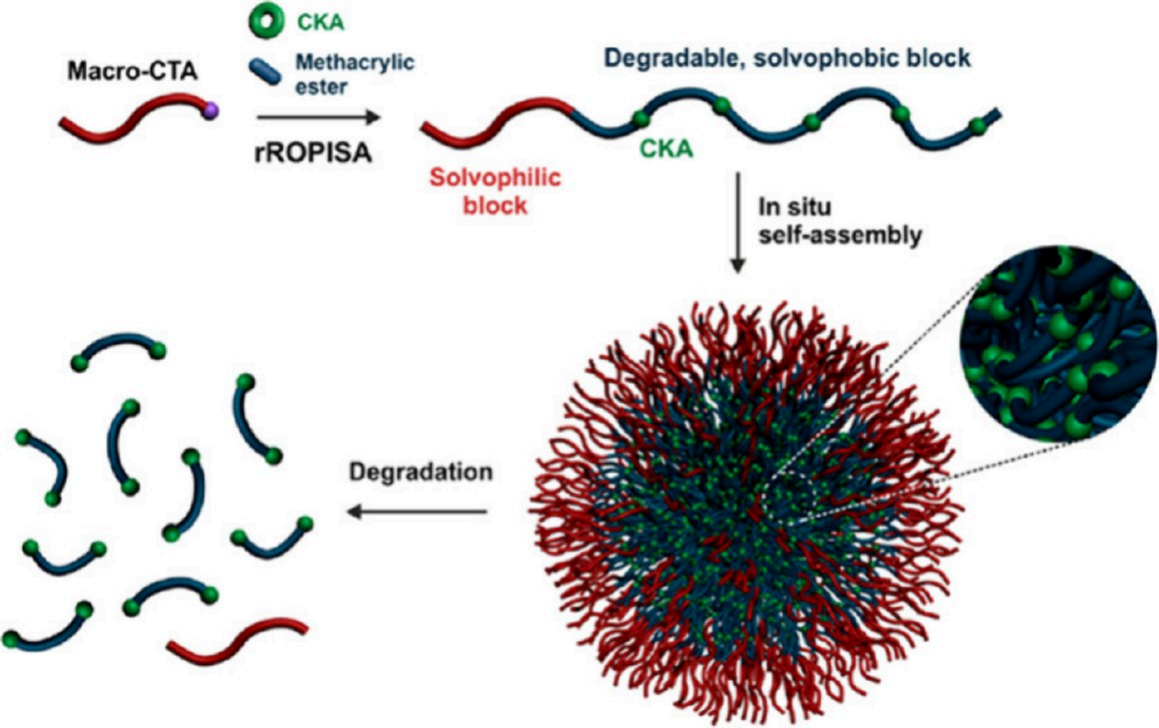
Synthesis of block copolymer nanoparticles with degradable cores via self-assembly induced by radical ring-opening copolymerization (rROPISA) mediated by RAFT from cyclic ketene acetals (CKAs). Reproduced from ref 196 with permission. Copyright 2019 American Chemical Society.

Using DMF, the same group showed that PPEGMA macroRAFT could mediate rROPISA of lauryl methacrylate in the presence of MDO, BMDO, and MPDL.^198^ Adjusting the molar mass of the hydrophilic PPEGMA chains helped to produce particles that could be directly redispersed in water after dialysis against water of the obtained stable DMF dispersion, or after an intermediated dialysis against acetone before dialysis against water. These nanoparticles were fully degraded under basic conditions and kept their degradability after transfer in water while showing no cytotoxicity.

Eventually, it is worth mentioning that, although not resorting to polymerization in dispersed media, some strategies based on nanoprecipitation in a selective solvent of preformed (block) copolymers containing CKA units can be used to achieve degradable particles.^199,200,93,168,170^ Circumventing the potential hydrolysis of CKA during polymerization in aqueous media, these strategies are particularly well suited for high value-added applications, but nevertheless less straightforward and probably more difficult to implement for large-scale syntheses.

#### Thionolactones (TL)

Thionolactones, particularly DOT, address certain limitations of CKAs, notably their hydrolytic sensitivity. D’Agosto, Lansalot, and co-workers^201^ prepared degradable latexes by conventional emulsion copolymerization of DOT with styrene and/or *n*-butyl acrylate in water, obtaining stable particles in less than 2 h with a broad range of *T*_g_ values and that could be degraded by aminolysis or strong base without prior hydrolysis (Figure 38).

**Figure 38:**
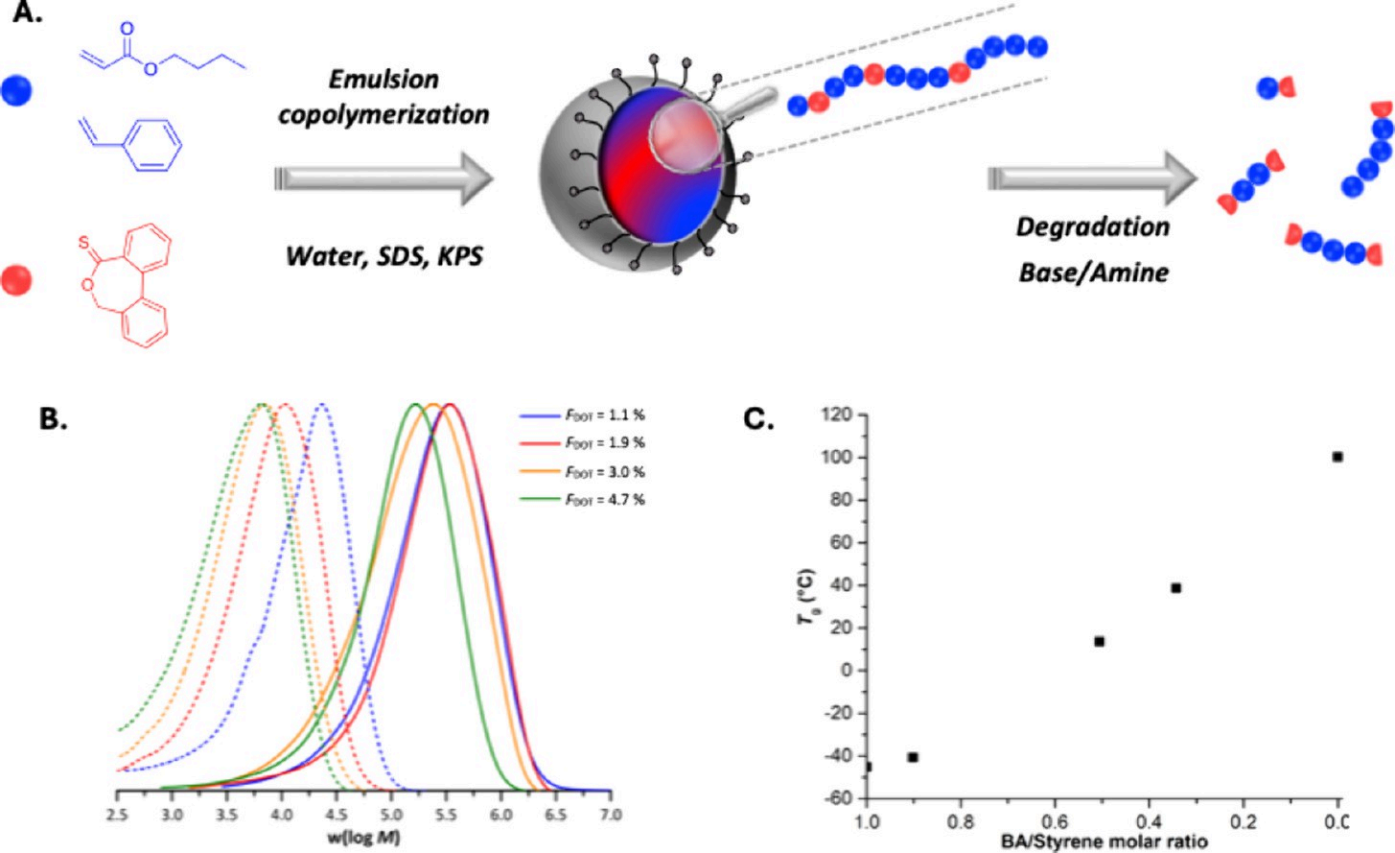
(A) Synthesis of SDS-stabilized latexes of P­(BA-*co*-DOT), P­(S-*co*-DOT), and P­(BA-*co*-*S-co*-DOT) by aqueous emulsion polymerization. (B) Molar mass distribution of the dry extracts of P­(S-*co*-DOT) latexes (plain lines) and their degradation products with TBD (dashed lines) as a function of incorporated DOT content (up to 4.7 mol %). (C) Evolution of the *T*_g_ depending on the average molar fraction BA/styrene in the monomer mixture for emulsion polymerization with 2 mol % of DOT. Adapted from ref 201 with permission. Copyright 2022 Wiley-VCH.

Noteworthy, P­(BA-*co*-DOT) particles could not only be degraded under their dried form, but also as waterborne particles. DOT remained effectively stable during the polymerization, but its solubility in the comonomer(s) was however limited (up to 3 mol % for styrene, and 2 mol % for BA). Using RAFT-mediated PISA, the same team then produced directly in water PEG- or poly­(*N*-acryloylmorpholine) (PNAM) stabilized nanoparticles with a core of either PS or PBA degradable under basic conditions (TBD).^202^

Nicolas and Armes et al.^203^ further took advantage of the hydrolytic stability of DOT in aqueous dispersion rROPISA to synthesize degradable vesicles. The polymerization was mediated by a hydrophilic poly­(*N*,*N*-dimethylacrylamide) chain transfer agent and a comonomer starved feed strategy was used to compensate for reactivity imbalances between DOT and acrylic monomers such as 2-methoxyethyl acrylate (MEA), enabling uniform and DOT incorporation up to 4 mol % in the hydrophobic block (Figure 39).^203^ The resulting vesicles gradually disassemble through thioester hydrolysis. Cargo release experiments using the hydrophobic dye Nile Red confirmed that degradation triggered by glutathione or l-cysteine enables efficient content release, suggesting strong potential for targeted drug delivery applications (Figure 39).

**Figure 39:**
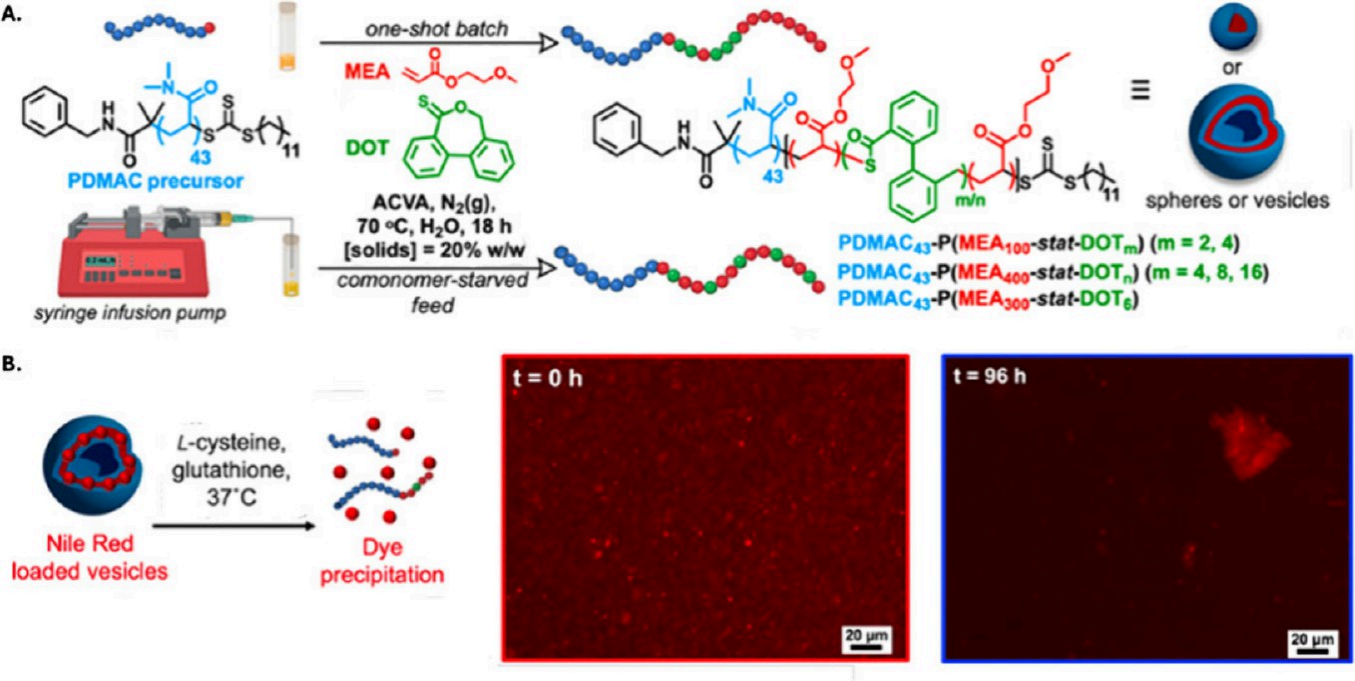
(A) Synthesis of PDMAC_43_-P­(MEA_100_-*co*-DOT_*m*_) (*m* = 2 or 4) spheres and PDMAC_43_-P­(MEA_300_-*co*-DOT_6_) and PDMAC_43_-P­(MEA_400_-*co*-DOT*_n_*) (*n* = 4, 8, or 16) vesicles via aqueous rROPISA with 20% w/w solids. The MEA/DOT mixture was added either all at once or gradually using a syringe pump (0.2 mL·h^–1^ over 2 h). (B) Scheme showing the Nile Red probe (red spheres) loaded in the membrane of PDMAC_43_-P­(MEA_400_-*co*-DOT_8_) vesicles. Degradation of these vesicles in the presence of 10 mM l-cysteine and 10 mM glutathione leads to precipitation of insoluble probes. (C) Fluorescence micrographs (λ_ex_ = 550 nm, λ_em_ = 605 nm) were recorded for 1% w/w dispersions at two time points (0 and 96 h) during hydrolytic degradation. Reproduced from ref 203 with permission. Copyright 2025 American Chemical Society.

In parallel, Nicolas et al.^204^ developed an approach based on NMP-mediated emulsion polymerization (Figure 40). Using a PAA-SG1 macroinitiator, copolymerization of DOT (1–3 mol %) with *n*-butyl acrylate or styrene was performed. Precise control over degradation could be triggered by aminolysis (e.g., *N*-isopropylamine) or basic hydrolysis (TBD).

**Figure 40:**
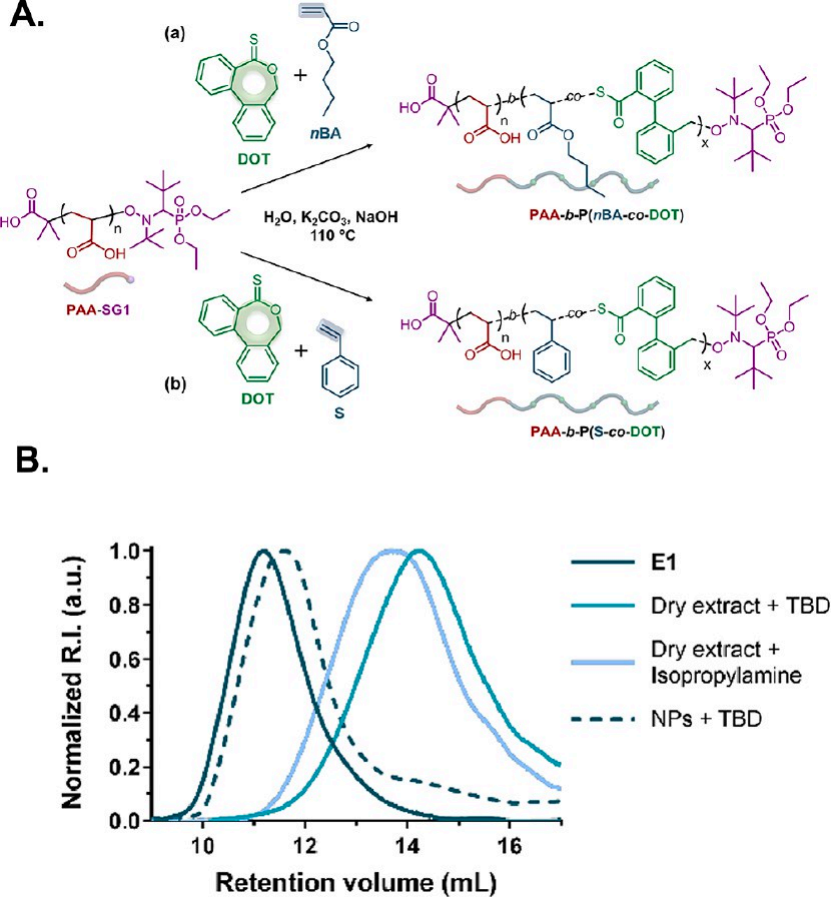
(A) Synthesis of PAA-*b*-P­(*n*BA-*co*-DOT) and PAA-*b*-P­(S-*co*-DOT) copolymers by rROPISA in water. (B) SEC traces of the dry extracts and NPs composed of a PAA-*b*-P­(*n*BA-*co*-DOT) copolymers with 1.3 mol % DOT before and after degradation in the presence of TBD or isopropylamine. Reproduced from ref 204 with permission. Copyright 2022 American Chemical Society.

With the aim of extending the range of aqueous heterogeneous processes leading to degradable particles, Nicolas et al.^205^ prepared DOT-free PBA or PS seeds by NMP using a conventional surfactant. These seeds were used for chain-extension with a mixture of DOT (1–3 mol %) with BA or styrene. The degradation of the obtained block copolymers could be triggered by TBD. DOT was also recently evaluated in a miniemulsion approach for the synthesis of either P­(S-*co*-DOT) or P­(BA-*co*-DOT) particles.^206^ Working in miniemulsion not only allowed faster polymerizations and higher molar masses than in solution, but also enabled incorporation of higher DOT content (up to 20 mol %). Both kinds of particles could be degraded using amines.

##### α-Lipoic Acid and Lipoates

More recently, lipoates, particularly α-lipoic acid (αLA) and its ethyl ester (ethyl lipoate, ELp), have re-emerged as promising comonomers for incorporating labile disulfide bridges into vinyl polymers via rROP. Morris et al.^129^ demonstrated the feasibility of producing degradable latexes of P­(BA-*co*-αLA) via miniemulsion polymerization (Figure 41). Up to 10 mol % of αLA units were incorporated into the chains (higher contents were not achievable due to the solubility limit of αLA in BA).

**Figure 41:**
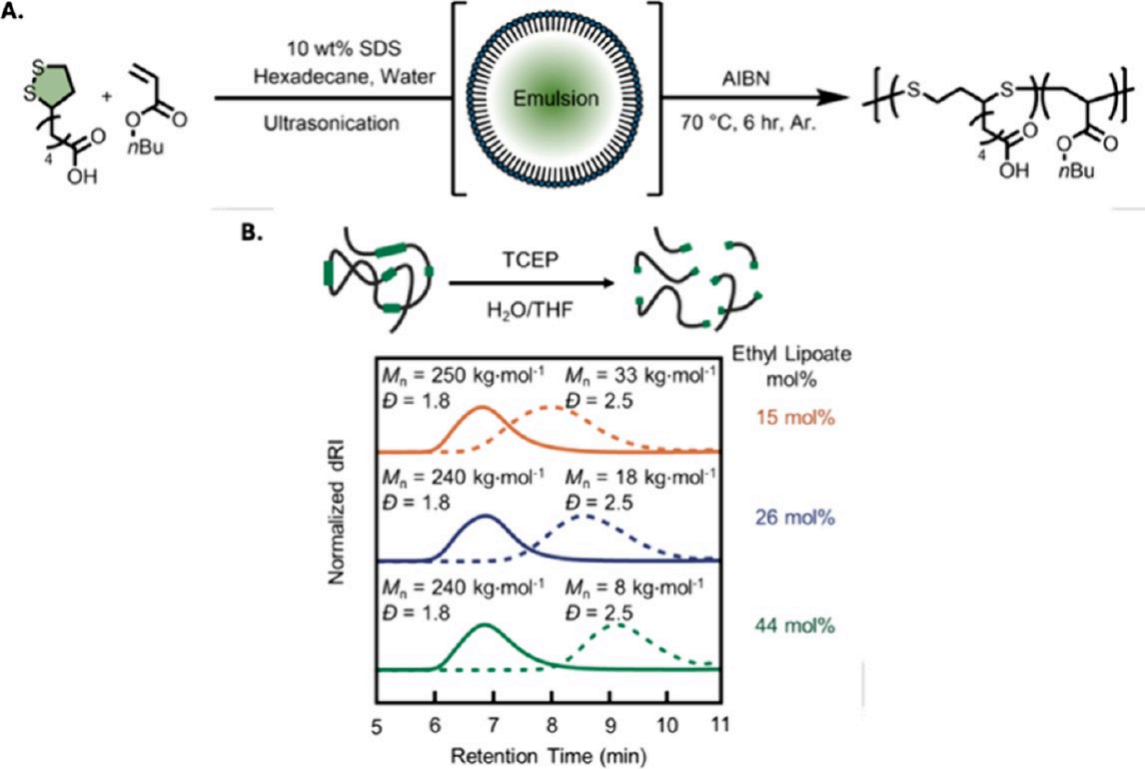
(A) Miniemulsion polymerization of α-lipoic acid with *n*-butyl acrylate. (B) Degradation for different amounts of ethyl lipoate with TCEP in an H_2_O/THF mixture. Reproduced from ref 129 with permission. Copyright 2024 American Chemical Society.

The resulting particles degraded efficiently in the presence of triscarboxyethyl phosphine (TCEP) (Figure 41). Large-scale synthesis (kg) using αLA with different hydrophobic monomers was also demonstrated, as well as the possibility of increasing the content of cleavable bonds by substituting αLA by ELp, fully miscible with BA.

Very recently, Zetterlund et al.^207^ have managed to copolymerize αLA with styrene and different (meth)­acrylates by direct emulsion polymerization. Stable latexes were produced, provided that azo initiators were used instead of more conventional persulfates. The copolymers were degraded by heating in DMF at 100 °C, a procedure that cleaves both S–S and C–S main chain bonds. In this work, unusual reactivities were observed with methacrylate derivatives and styrene.

Successful emulsion copolymerization of styrene with *tert*-butyl lipoate (*t*BLp) was also recently reported.^116^ The high molar mass polymers obtained could be partially degraded upon addition of DTT or under UV irradiation, both of which specifically affect the S–S bonds present when two units of *t*BLp are adjacent.

##### Sulfide Cyclic Methacrylate (SCM)

Nanoparticles of various morphologies were prepared by Armes, Paulusse, and co-workers^149^ using a disulfide containing-sulfide cyclic methacrylate (SCM7, Figure 9) in RAFT-mediated dispersion PISA. This monomer was copolymerized in water with hydroxypropyl methacrylate in the presence of a hydrophilic poly­(glycerol monomethacrylate) macroRAFT. Good control of the polymerization and thus of particle morphology required a low amount of SCM7 to be used (0.5 mol %). Addition of TCEP induced the expected reduction of *M*_n_, which led to an irreversible worm-to-sphere transition.

### Degradable Surface Coatings

(Bio)­degradable functional or functionalizable surface coatings are of significant interest in the industrial sector. Various techniques for surface modification utilizing rROP copolymerization of cyclic monomers with various vinyl monomers have been documented in the literature to address these requirements. Klok et al.^208^ developed surface coatings in the form of polymer brushes grafted onto silica surfaces, obtained by copolymerizing poly­(ethylene glycol) methacrylate (PEGMA) with the cyclic monomer BMDO (Figure 42A).

**Figure 42:**
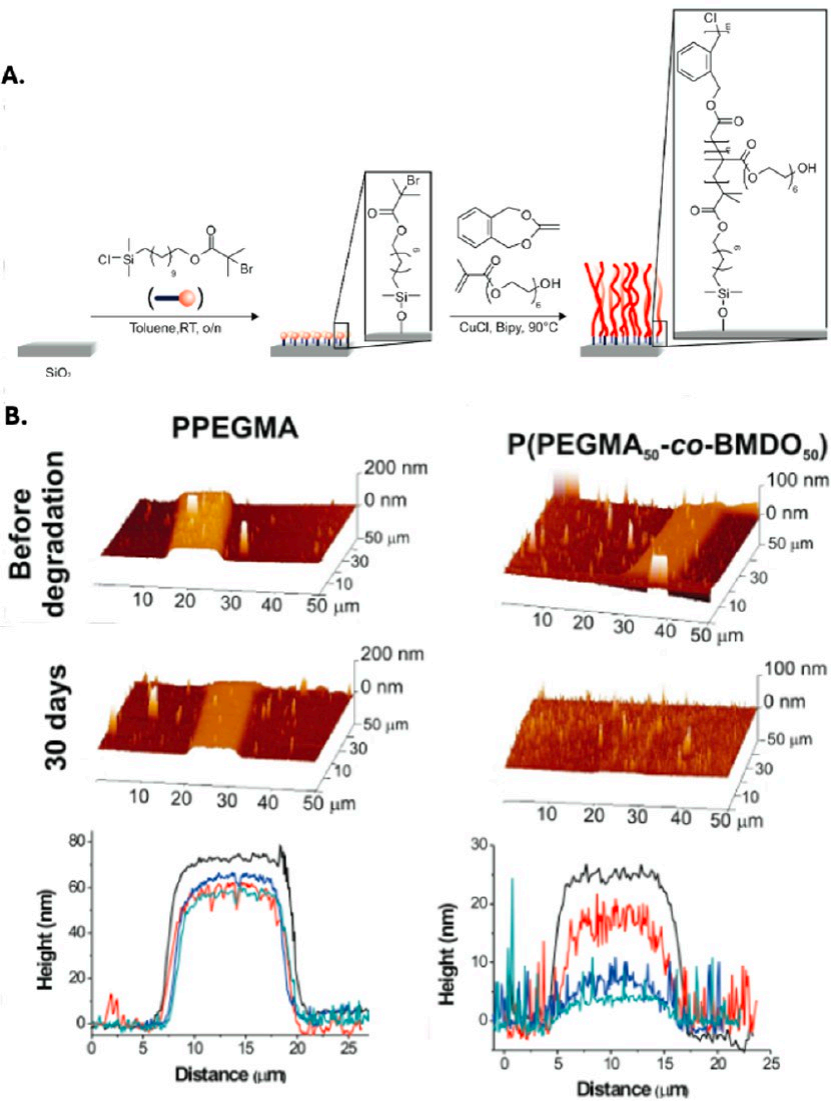
(A) Preparation of surface coatings in the form of polymer brushes grafted onto silica surfaces, obtained by copolymerizing poly­(ethylene glycol) methacrylate (PEGMA) with the cyclic monomer BMDO. (B) 3D AFM images and 2D cross-sectional profiles of P­(PEGMA) brushes with and without BMDO, taken at different time intervals during exposure to a pH 3 solution at 25 °C. Reproduced from ref 208 with permission. Copyright 2009 American Chemical Society.

These films exhibit remarkable stability under basic conditions (pH 9) but degrade rapidly in acidic environments (pH 3), with degradation kinetics tunable according to the PEGMA/BMDO ratio (Figure 42B). Monitoring of degradation by AFM and ellipsometry over 30 days highlighted the potential of such systems in pH-sensitive applications.

In another approach, Lahann and colleagues^209^ introduced rROP into a chemical vapor deposition (CVD) process, which is typically nondegradable. By copolymerizing BMDO with [2,2]­paracyclophanes, they obtained ultrathin films capable of eroding under basic conditions via surface hydrolysis, while maintaining excellent cytocompatibility, as confirmed by XTT assays. This development represents a major step toward biodegradable coatings for implantable devices.

In the field of antifouling, Zhang et al.^210^ have made numerous contributions. In 2015, they designed marine coatings by copolymerizing MDO with methyl methacrylate (MMA), incorporating an organic biocide (DCOIT) to enable controlled release that was adjustable according to the ester content. Tests under marine conditions demonstrated prolonged efficacy for over three months. Continuing this strategy, in 2016, the team incorporated hydrophilic silyl ester groups derived from TBSM into the copolymers to increase water uptake and promote film erosion. This modification significantly enhanced the degradation rate in marine environments.^211^

In 2019, Zhang et al.^212^ developed a new generation of MMA/MDO copolymers by covalently grafting antifouling agents such as *N*-methacryloyloxymethyl benzisothiazolinone (BIT). These systems, tested in seawater, exhibited remarkable resistance to biofouling, with a limited mass loss of 7.3% after 90 days. More recently, the team designed self-regenerating surfaces based on MMA, MDO, and a zwitterionic precursor monomer (HIZ), capable of switching from a hydrophobic to a hydrophilic state through hydrolysis (Figure 43).^213^ This transformation releases zwitterionic groups that confer antiprotein, antibacterial, and antidiatom properties to the surfaces, paving the way for smart coatings capable of restoring their function upon contact with water.

**Figure 43:**
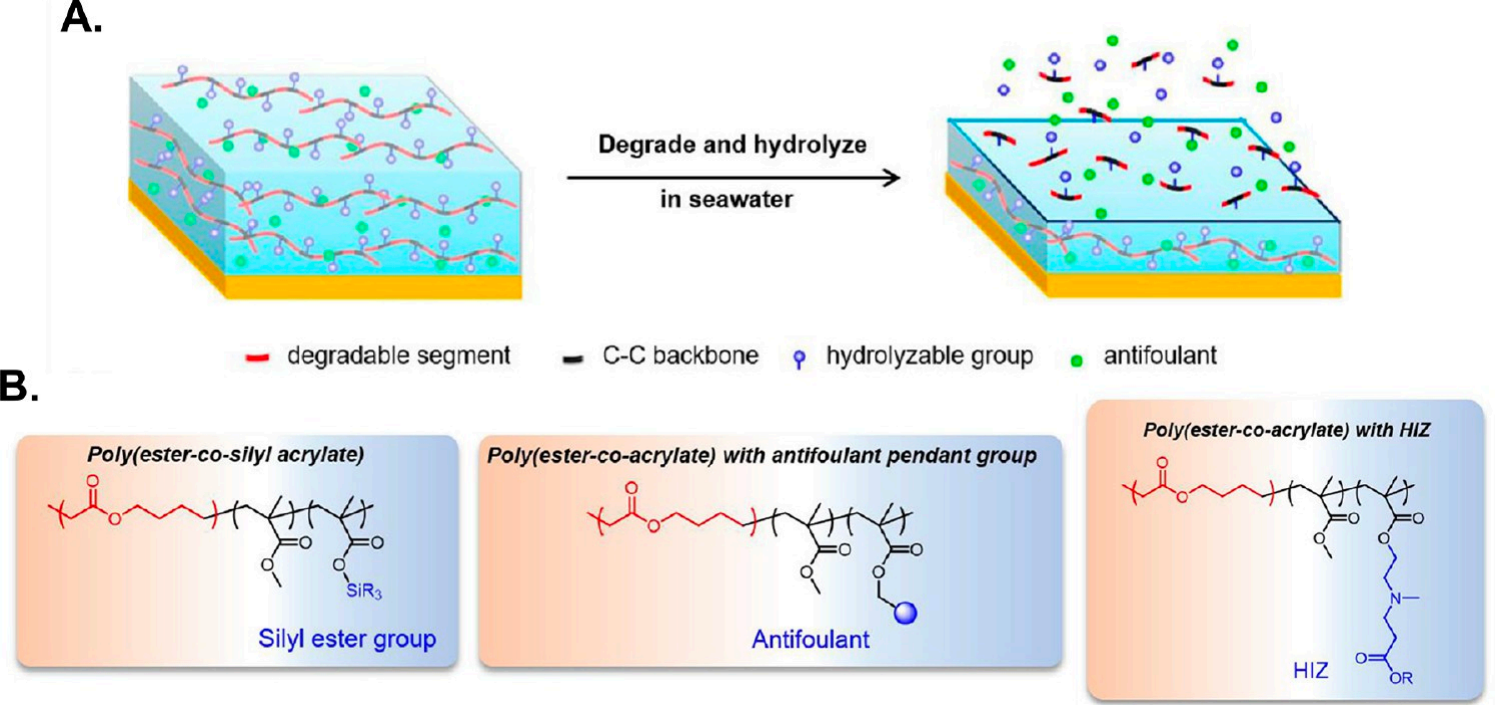
(A) Antifouling mechanism of degradable and hydrolyzable polymers. (B) Structures of degradable and hydrolyzable polymers. Reproduced from ref 213 with permission. Copyright 2022 American Chemical Society.

Lastly the same group prepared a a cleavable hyperbranched poly­(ester-*co*-vinyl) with diethylene glycol units and unreacted pendant vinyl groups via the copolymerization of MDO, VAc, and diethylene glycol divinyl ether.^214^ This material could spread over a surface and UV-cross-linked to create a degradable antifouling coating. The polymer with diethylene glycol units exhibits remarkable antifouling abilities and can effectively inhibit the adhesion of protein, marine bacteria (*Pseudomonas sp*.), and diatoms (*Navicula incerta*).^214^

### Degradable Thermoset

Polymer gels/networks are key components in several consumer items, including automotive tires, coatings, building materials, contact lenses, superabsorbents, and 3D objects obtained by additive manufacturing. In the presence of divinyl cross-linkers, FRP results in fast, uncontrolled chain growth, producing a heterogeneous network topology and the production of dense nano- or microclusters, making these materials nondegradable.

Including cleavable cross-linkers is a classic and effective method to introduce degradability to gels and networks derived from vinyl polymers. Kopec et al.^215^ conducted a thorough investigation of the degradation of various polymer networks synthesized using free-radical polymerization and disulfide-containing cross-linkers. Notably, polymethacrylate and polystyrene networks completely degraded and dissolved, but only at relatively low cross-linker loadings (<2 mol % relative to monomer). Conversely, polyacrylate and polyacrylamide networks exhibited no degradation at any cross-linking densities. This was ascribed to the presence of microclusters that form due to the rapid polymerization and extensive intramolecular cyclization. These heterogeneous structures do not swell, which prevents a small fraction of the disulfide bonds from being cleaved. Unlike with cleavable cross-linkers, the same group showed that using cleavable comonomers (in this case the DOT thionolactone) produced polyacrylate networks containing 1 mol % of cross-linkers that could be fully solubilized via aminolysis when 4 mol % of DOT was used.^216^ In a further study, the authors compared the use of RDRP versus uncontrolled conventional free-radical polymerization and thus the homogeneity of the network on the regelation of degraded polyacrylate networks.^217^ Under similar degradation conditions using cysteamine/DBU, polyacrylate networks made using conventional polymerization (4 mol % DOT and 1 mol % cross-linker) cannot regel via the creation of disulfide bonds, whereas similar degradation products obtained using the addition of a RAFT agent did regel with successful cycling of reductive degradation/oxidative gel formation. The more homogeneous network structure obtained by RAFT-made gels is intriguing for controlling efficient reversibility.^217^ Dawson et al.^218^ extended these previous studies to lipoate derivatives and demonstrated that copolymerization of αLA or ELp with *n*-butyl acrylate via uncontrolled FRP or RAFT produced cross-linked networks that are degradable in the presence of thiols or upon heating (100 °C, DMF). Upon degradation, these networks release soluble thiol-rich fragments, which can be recross-linked by oxidation under basic conditions (DBU, triethylamine). Network regeneration was more efficient for materials prepared by RAFT and incorporating ELp, due to better structural homogeneity and lower dispersity of the fragments.^218^ In another study, Choi et al.^219^ developed dynamic “bottle-brush” type elastomers based on PDMS and lipoate groups. Through photoinduced polymerization of PDMS chains functionalized with mono- and bis-lipoates, they obtained lightly cross-linked networks (gel fraction 83–98%) with very low shear moduli (20–200 kPa). These materials exhibit reversible liquefaction at 180 °C (via disulfide bond cleavage) and can be repaired under UV light. Like the materials described above, these bottlebrush elastomers are also degradable under basic or reducing conditions, paving the way for supersoft and recyclable/reconfigurable materials that can function as adhesives.^220^

Another exciting use of degradable thermosets is to enhance DNA storage inside deconstructible glassy polymer networks, drawing inspiration from the millennia-long preservation of fossilized biological specimens in calcified rocks or glassy amber. Johnson and co-workers^221^ demonstrated the direct transfer of DNA from aqueous solution to organic solvent (e.g., styrene) using a carefully designed terpolymer-based polyplex, accommodating lengths from tens of nucleotides to over 50,000 base pairs, thereby eliminating the necessity for prolonged drying under reduced pressure. This facilitated the encapsulation of complexed DNA within hours using conventional radical polymerization of styrene, divinylbenzene as cross-linker (1 mol %) and 2-(isopropylthio)­dibenzo­[c,e]-oxepine-5­(7*H*)-thione (2SiPrDOT) (3 mol %), which is considerably more rapid than silica and calcium phosphate encapsulation methods that need days (thermoset-reinforced xeropreservation T-REX method, Figure 44a).

**Figure 44:**
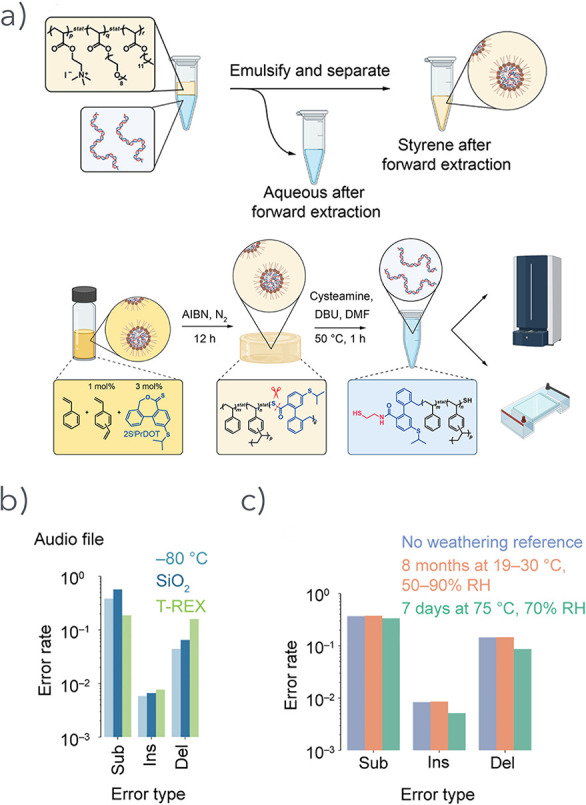
(a) Scheme for producing T-REX thermosets from polyplexes, reversible encapsulation, and subsequent characterization. (b) A comparative analysis of error rates in 210-bp dsDNA segments encoding digital data between samples stored in a frozen state without encapsulation and DNA recovered from both T-REX and silica-based encapsulated samples. (c) Comparison of error rates of T-REX-encapsulated samples containing 210-bp dsDNA encoding an image file subjected to real-time and accelerated weathering conditions. Reproduced from ref 221 with permission. Copyright 2024 American Chemical Society.

Mild deconstruction of the styrene-based thermoset using cysteine/DBU treatment enabled the recovery of the DNA without compromising the integrity of the DNA. Weathering tests demonstrated that T-REX retains DNA under accelerated aging settings more effectively than calcium phosphate and silica encapsulation. Sequencing results confirm T-REX’s capacity to encapsulate DNA without bias or mutations, illustrating its effectiveness for high-fidelity DNA data storage and whole genome sequencing applications (Figure 44b).

Finally, 3D printing has the potential to transform the industrial sector by enabling fast and customized manufacturing of objects with great precision, straight from computer-aided design. VAT photopolymerization is now the most used 3D printing methods and is based on the cross-linking of liquid resins using light irradiation. Currently, commercially available resins consist of (meth)­acrylate monomers and cross-linkers, sometimes containing a second type of monomer which can be polymerized orthogonally in a second thermal step. The resultant object is a cross-linked material with a C–C type skeleton. This design yields excellent thermal and mechanical properties; it also results in the production of nondegradable objects.

Using a very specific setup, i.e., direct laser writing via 2-photon absorption, Carlotti et al.^222^ demonstrated the potential of this process in the fabrication of biodegradable microstructures by copolymerizing MDO with multiacrylates (90:10) (Figure 45a). This method enables the production of high-resolution microstructures that degrade in less than an hour under acidic or basic conditions (Figure 45b), making them suitable for applications such as degradable protective masks or microscaffolds. However, due to the low reactivity of CKAs, only a high *f*_CKA,0_ could be used, leading to a low cross-linked polyester, and thus this formulation was limited to microscale geometries.

**Figure 45:**
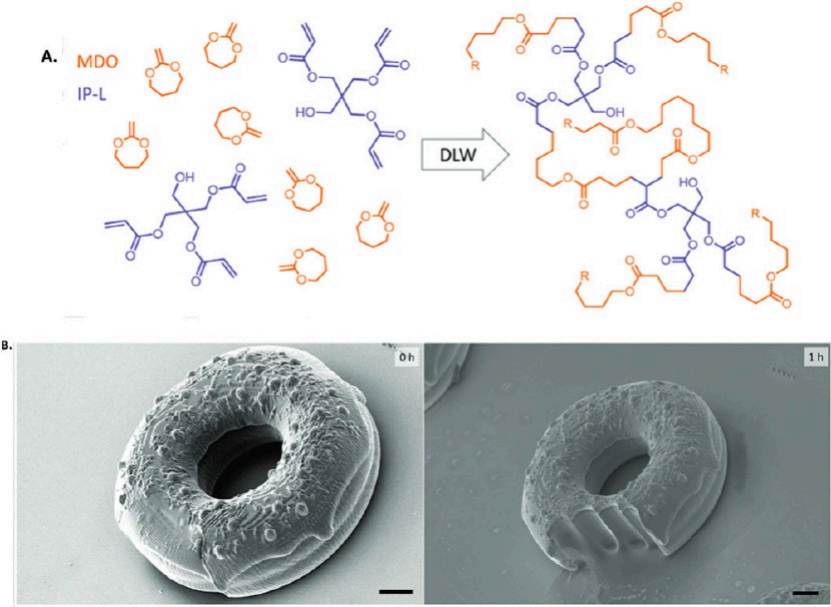
(a) During the DLW process, aliphatic polyester units are incorporated into the cross-linked network; after treatment with a nucleophile (Nu^–^), these units break down, degrading the microstructure. Cleavage of the ester bond by the nucleophile occurs between the carbonyl carbon and the oxygen (not shown for clarity). (b) Partial degradation (SEM images) of microdonut structures fabricated with MDO:PETA 90:10 (scale bar 20 μm). Only the final part degrades, leaving a bite mark. Reproduced from ref 222 with permission. Copyright 2022 Wiley-VCH.

Guillaneuf and colleagues^223^ introduced a generic approach to make 3D-printed objects degradable by incorporating only 2 wt % of DOT into commercial multiacrylate resins (Figure 46A). Radical ring-opening polymerization (rROP) introduces thioester bonds into the network, triggering degradation under basic conditions (5% KOH in THF/MeOH). This approach proved compatible with various techniques, including two-photon stereolithography, UV microfabrication, and standard 3D printers.

**Figure 46:**
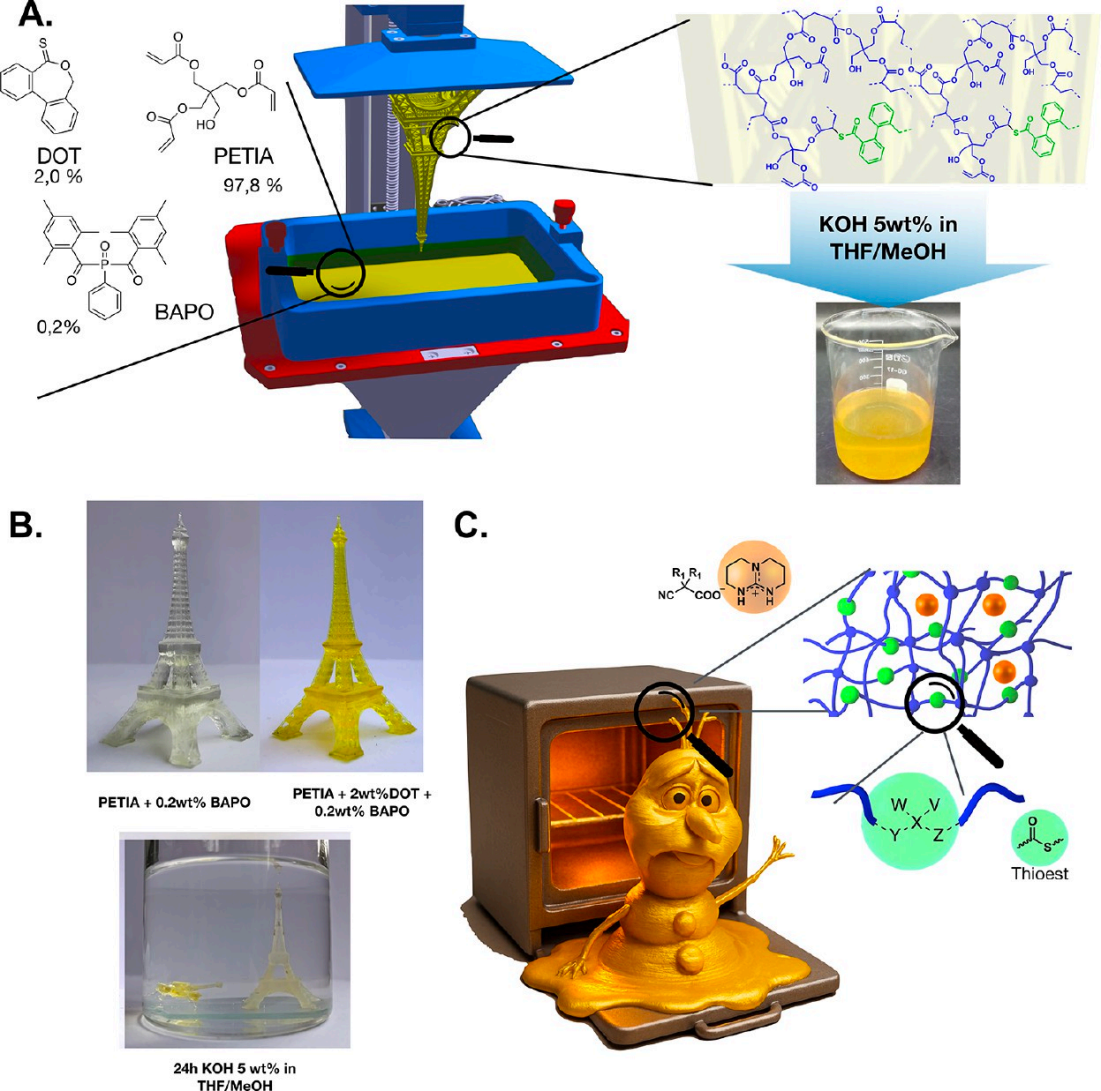
(A) Preparation of degradable 3D objects by VAT photopolymerization and chain cleavage. (B) Example of a 3D product with and without cleavable copolymer additive. Reproduced from ref 223 with permission. Copyright 2022 Wiley-VCH. (C) Concept of self-destruct materials via the combination of thermolatent base and cleavable comonomers.

The rate of degradation is restricted by the diffusion of base into the materials.^223^ Guillaneuf et al.^224^ thus proposed to combine the cleavable comonomer approach with the addition of various 1,5,7-triazabicyclo[4.4.0]­dec-5-ene (TBD) salts to obtain objects with built-in degradability that could be activated thermally or photothermally and lead to spatially controlled degradation (Figure 46B). Applications to all organic thermal fuses were demonstrated as well as the recovery of an embedded 3D object into a thermoset after simple heating, illustrating the interest of this methodology for preparing materials with programmed degradation.

Choi et al.^133^ developed supersoft bottlebrush networks based on the copolymerization of PEG functionalized with lipoates and PEG diacrylates under light (≤405 nm) in the presence of a photoinitiator, combining elasticity, thermal stability, and self-healing behavior through dynamic disulfide bond exchange.

These materials can be repaired either by heating or at room temperature, providing enhanced durability for complex printed objects. Another study by Han et al.^225^ focused on the design of new photoactive resins for 3D printing by incorporating two cross-linkers derived from lipoic acid: DIS-Lp2, featuring a dynamic disulfide bond, and TEG-Lp2, based on a nondynamic ethylene glycol unit. These cross-linkers were formulated with *n*-butyl acrylate (BA) and a photoinitiator and tested with a commercial LCD printer (Figure 47A). Photopolymerization and printing tests showed that formulations rich in DIS-Lp2 enabled the fabrication of complex high-resolution objects with nearly complete monomer conversion.

**Figure 47:**
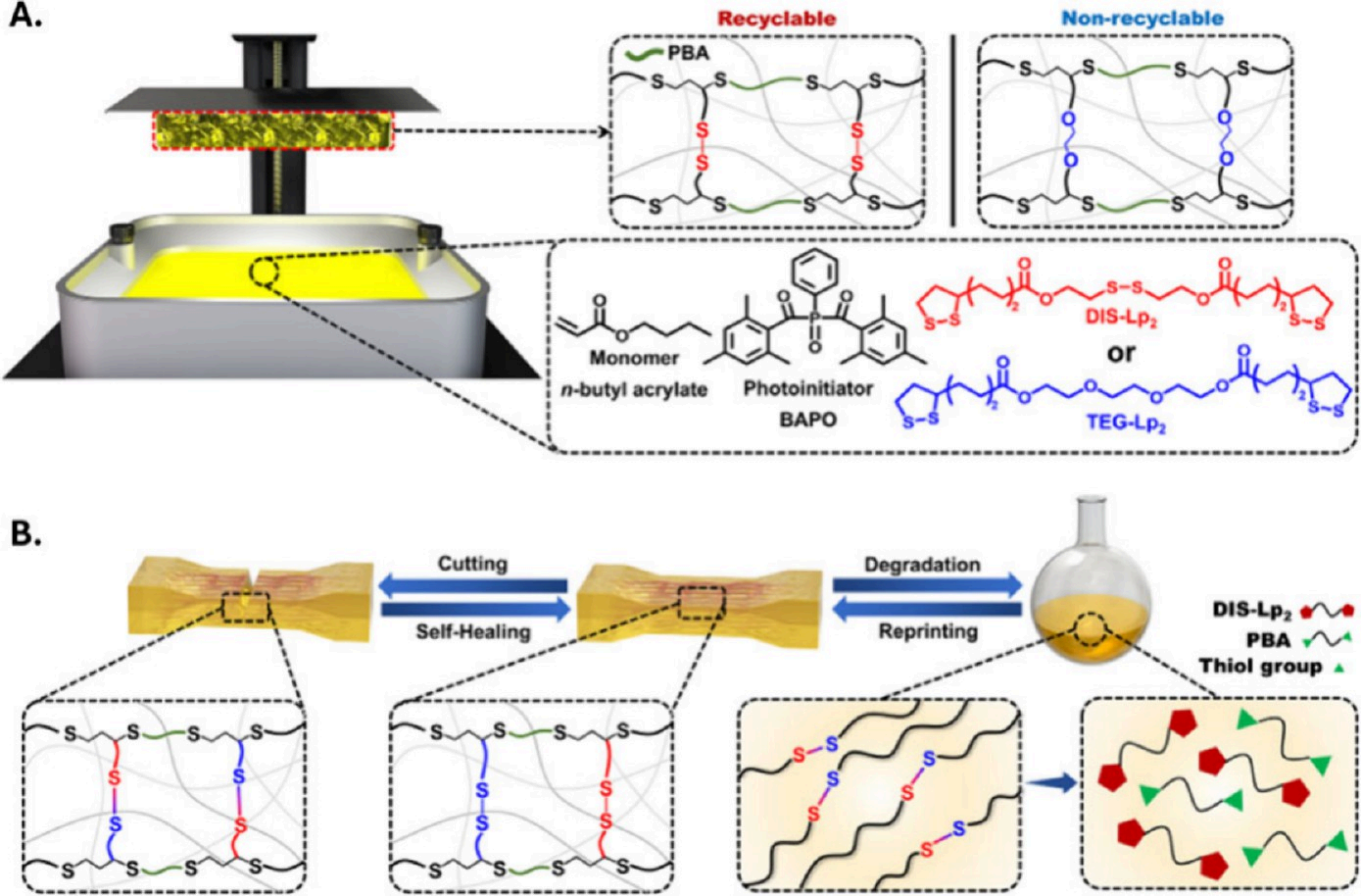
(A) Chemical structures of a 3D printing resin derived from α-lipoic acid building blocks. The resin components include *n*-butyl acrylate, a mixture of cross-linkers DIS-Lp2/TEG-Lp2, and the photoinitiator (BAPO). (B) Diagram illustrating self-healing, degradation, and recycling of the printed material using DIS-Lp2 as the cross-linker. Reproduced from ref 225 with permission. Copyright 2024 American Chemical Society.

The resulting materials exhibit remarkable properties: self-healing (in the presence of DBU at 60 °C), controlled degradability, and recyclability (Figure 47B).^225^ Lastly, Dove and co-workers reported fully lipoate-based resins, derived from α-lipoic acid, as a biobased solution that can be printed without solvents or toxic additives while remaining recyclable (Figure 48a).^81^ Formulated from lipoate monomers such as menthyl or ethyl lipoate combined with multifunctional lipoate cross-linkers, these resins enable high-resolution printing (up to 100 μm) on standard DLP printers (Figure 48b).

**Figure 48:**
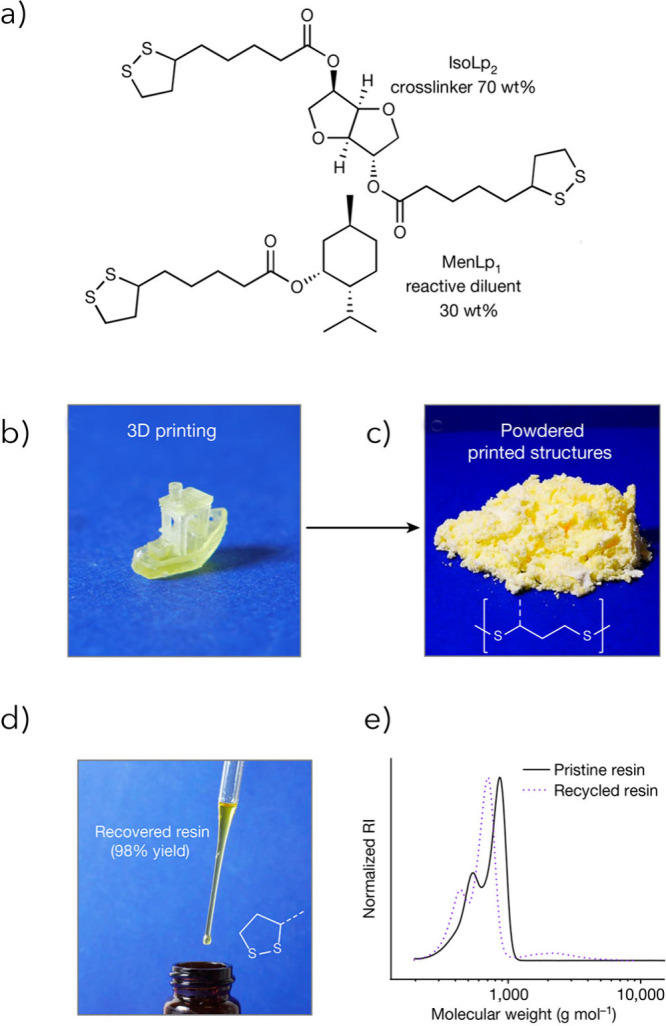
Method enabling polymerization–depolymerization cycles of dynamic disulfide bonds, allowing for the formulation of 3D-printing resins from renewable sources that are suitable for closed-loop chemical recycling. (a) Chemical composition of the formulated resin. (b) An example of a complex 3D-printed part. (c) Photograph of 3D-printed parts in powder form. (d) Photograph of the resin recovered in 98% yield after depolymerizing a 3D-printed part. e) SEC of initial resin compared to recovered resin. Adapted from ref 81 with permission. Copyright 2024 Springer Nature.

Notably, they can be chemically or thermally depolymerized to recover up to 98% of the original monomers, which can be reused without any loss of performance. Some formulations already achieve mechanical properties comparable to those of commercial soft resins (modulus up to 340 MPa, tensile strength up to 50 MPa).^81^

### Degradable Biomaterials

For many applications, the biomedical sector requires the use of degradable materials. Biodegradation without accumulation of polymers in the body or the generation of hazardous degradation products is crucial for resorbable materials or active ingredients/drug delivery systems. Extensive research has been conducted on the use of polyesters, polypeptides, natural polymers, and other similar materials. Although the adaptability and ease-of-use of radical polymerization make it a very promising and convenient method for the synthesis of materials for biomedical applications, the C–C carbon backbone of vinyl polymers prevents their (bio)­degradation.

One of the main applications of degradable polymers in nanomedicine concerns the development of nanoscale drug delivery systems. In this context, various types of nanoparticulate systems were designed, usually obtained by formulation of preformed degradable diblock copolymers obtained by rROP.

For instance, PVA-*b*-PMDO diblock copolymers were obtained by sequential RAFT polymerization of VAc and MDO in bulk, followed by removal of the acetyl groups.^199^

Model molecules were encapsulated and the nanoparticles successfully released their payloads *in vitro*. Alternatively, free-radical copolymerization of MDO and VAc produced poly­(VAc-*co*-MDO) random copolymers that were further formulated into nanoparticles for the encapsulation of model drugs.^200^ A comparative study showed that, despite their structural analogy, mPEG-*b*-PCL and mPEG-*b*-PMDO exhibited different drug release behavior due to significant differences in their microstructure,^226^ as the former demonstrated semicrystalline behavior, while the later displayed a more amorphous nature due to branching.

More sophisticated degradable amphiphilic diblock copolymers were also obtained by RAFT-mediated terpolymerization of MDO, VA, and vinyl bromobutanoate/vinyl levulinate.^227,228^ After azidation of the bromine groups and self-assembly, cross-linked, degradable micelles and polymersomes encapsulating doxorubicin were successfully obtained.^228^

pH-Responsive drug delivery systems obtained by rROP have also been reported, by copolymerization of MTC and iPRr-MAC,^229^ or 2-(diethylamino)­ethyl methacrylate and MDO,^93^ resulting in pH-induced controlled release/disassembly or self-assembly, respectively. Another example of an rROP-derived stimuli-sensitive drug delivery system concerns the synthesis of main chain degradable star hyperbranched copolymers comprising pH-responsive hyperbranched cores and thermoresponsive PEG coronas,^95^ obtained by RAFT copolymerization of 2-(diethylamino)­ethyl methacrylate, di­(ethylene glycol) dimethacrylate and MDO, followed by chain extension with OEGMA and MDO.

Degradable polyester-like glycopolymer nanoparticles were obtained by a combination of rROP of MDO with VE derivatives,^170^ and a Pd-catalyzed thioglycoconjugation,^169^ followed by the formulation of the resulting glycopolymers. Nanoparticles and their degradation products showed good cytocompatibility on healthy cell lines, whereas the interactions between nanoparticles and lectins revealed the coexistence of both specific carbohydrate–lectin binding and nonspecific hydrophobic interactions. The nanoparticles were successfully internalized by lung adenocarcinoma (A549) cells, underscoring the strong potential of these glycopolymers for biomedical applications. Interestingly, MTC-based glyconanoparticles obtained following the same protocol showed a similar cytocompatibility toward two healthy cell lines but a much stronger lectin affinity than the MDO-based counterparts.^168^

To combine degradability and adjustable drug release, Nicolas et al.^230^ explored the development of degradable polymeric prodrugs by copolymerizing MPDL and either MMA or OEGMA via NMP, initiated from a functionalized gemcitabine alkoxyamine initiator (Figure 49a). While MMA-based copolymer prodrugs produced polymer prodrug nanoparticles, OEGMA-ones yielded water-soluble polymer prodrug chains. Drug-release profiles in human serum and in vitro anticancer assays on two cancer cell lines revealed key structure–activity relationships, identifying optimal structural parameters. Three factors independently governed activity: (i) OEGMA-based prodrugs were more cytotoxic than MMA-based ones; (ii) lower MPDL content increased anticancer activity; and (iii) a diglycolate polymer-drug linker afforded greater activity compared to a simple amide bond.

**Figure 49:**
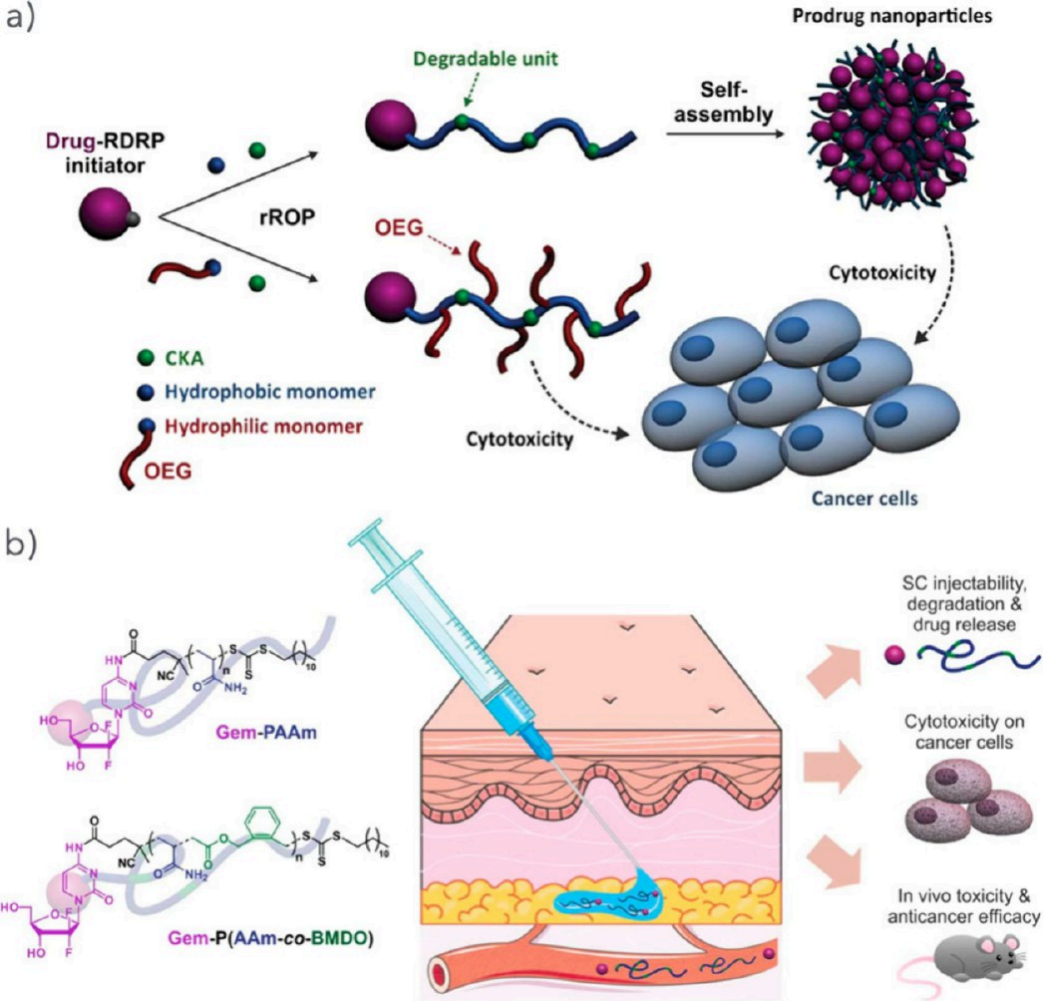
a) Synthesis strategy for the design of gemcitabine-based degradable polymeric prodrugs via nitroxide-mediated polymerization initiated by a Gem-alkoxyamine initiator. Reproduced from ref 230 with permission. Copyright 2018 Royal Society of Chemistry. b) Design and preclinical development of (degradable) polyacrylamide (PAAm)-based prodrugs for the SC administration of the anticancer drug gemcitabine (Gem). Reproduced from ref 231 with permission. Copyright 2025 Royal Society of Chemistry.

Degradable vinyl polymer prodrug nanoparticles can also be obtained *in situ* by rROPISA, to address both the limitations of traditional formulation of preformed polymers (e.g., low nanoparticle concentrations) and those of the physical encapsulation of drugs (e.g., burst release and poor drug loadings).^232^ In their work, Nicolas et al. performed chain extension of a POEGMA macro-RAFT agent by a mixture of lauryl methacrylate (LMA), drug-bearing methacrylic esters (based on Gem or paclitaxel), and CKA monomers (BMDO or MPDL). Stable core-degradable polymer prodrug nanoparticles (56–225 nm) containing 7–26 mol % CKA and up to 33 wt % drug loading were obtained.

The nanoparticles showed significant cytotoxicity against A549 lung cancer cells, and their fluorescence labeling enabled confocal tracking of cell uptake, highlighting their theranostic potential. The same prodrug strategy was also developed by Nicolas et al.^231^ to prepare degradable water-soluble polymer prodrugs for subcutaneous delivery of irritant anticancer drugs (Figure 49b). The polymer prodrugs turned degradable by incorporating ester groups into the main chain via the reversible ring-opening polymerization of BMDO with AAm during the “drug-initiated” synthesis. Degradable polymer prodrugs were readily injectable under clinically pertinent subcutaneous injection circumstances, even at elevated dosages. Significantly, the subcutaneous injection of Gem-PAAm degradable variants at elevated dosages in mice did not cause local toxicity or inflammation, in contrast to free Gem, that induced significant inflammation and necrotic regions. This indicated that these prodrugs can be administered subcutaneously without the BMDO units and their byproducts becoming hazardous.

Single-chain cross-linking offers an effective method for producing sub-30 nM nanoparticles, mainly for nanomedicine applications. Unlike a traditional degradation procedure that is based on the degradation of the cross-linker, such nanoparticles with main-chain degradability should yield oligomeric degradation products, possibly enhancing environmental or in vivo biodegradability. Degradable single-chain nanoparticles (DSCNP) have been synthesized by Jackson and Thoniyot et al.^94^ using the copolymerization of MDO with methacrylic acid *N*-hydroxysuccinimide ester (NHSMA), followed by intramolecular cross-linking through amide bond formation. The degradation under accelerated alkali conditions led as expected to a strong decrease of the *M*_n_. Paulusse et al.^148^ developed similar degradable nanoparticles through intramolecular cyclization of polymer chains (Single-Chain Technology) by copolymerizing a disulfide-based cyclic monomer (MDTD). These particles, sensitive to a reducing environment mimicking the cytosol, rapidly degrade in the presence of hydrazine hydrate (*M*_n_ decreasing from 9.9 to 2.8 kDa within 30 min). They demonstrated good transfection efficiency in 3T3 and HeLa cells without inducing toxicity.

In a different context, Maynard et al.^233^ designed poly­(BMDO-*co*-BMA-trehalose) glycopolymers capable of stabilizing thermosensitive proteins such as G-CSF at 40 °C (stabilization rate 66%). However, the degradation products were found to be cytotoxic to murine fibroblasts and myeloblasts, limiting their clinical applicability. For cryopreservation, Gibson et al.^158^ first reported the copolymerization of chloro-vinyl acetate with BMDO, which allowed access to ice-binding poly­(vinyl alcohol) with ester groups in the main chain for degradation. The Nicolas’ and Gibon’s groups^234^ developed polyampholyte copolymers poly­(DMAEMA-*co*-MAA-*co*-BMDO), also synthesized via RAFT polymerization (Figure 50A).

**Figure 50:**
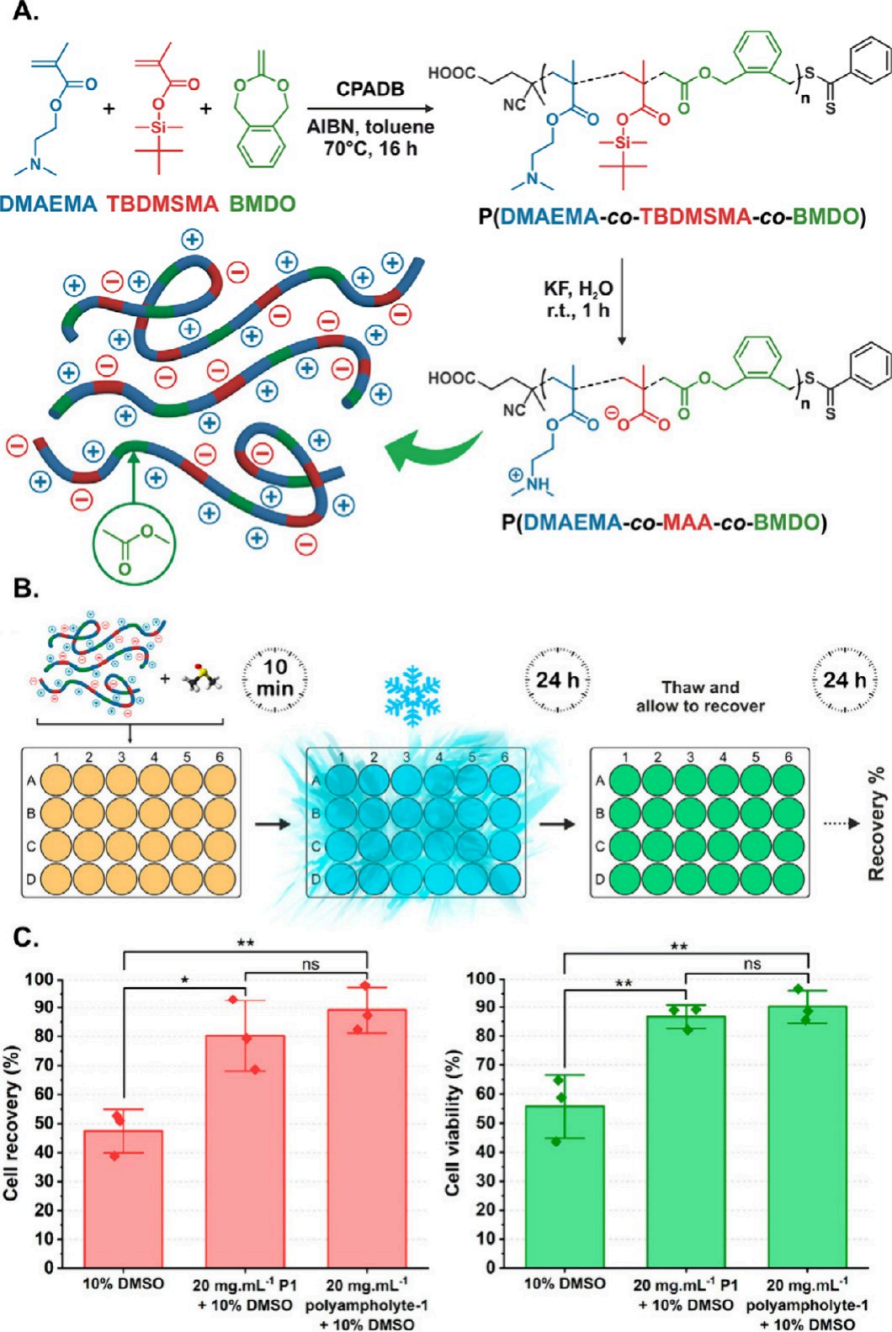
(A) Synthesis of poly­(*N,N*-dimethylaminoethyl methacrylate-*co*-methacrylic acid-*co*-5,6-benzo-2-methylene-1,3-dioxepane) poly­(DMAEMA-*co*-MAA-*co*-BMDO) terpolymers by RAFT terpolymerization of DMAEMA, TBDMSMA, and BMDO with CPADB as a RAFT agent, followed by deprotection of TBDMSMA units.^234^ (B) Schematic of the monolayer cryopreservation and post-thaw process. (C) Cell recovery 24 h post-thaw, relative to prefreezing, determined using Trypan blue exclusion test (left) and cell viability 24 h post thaw determined using Trypan blue exclusion test (right). One-way ANOVA with Tukey’s posthoc test. * = *p* < 0.05, ** = *p* < 0.001 considered as statistically significant different using a 95% confidence level, ns = not significant. Reproduced from ref 234 with permission. Copyright 2022 American Chemical Society.

These new degradable polyampholytes were noncytotoxic under cryopreservation conditions and significantly improved post-thaw yield and viability in a challenging cell monolayer model, showing that diluting charged monomers with ester units did not compromise cryoprotective activity (Figure 50B,C).

To find relevant alternatives to antimicrobial peptides to combat multidrug-resistant pathogens, degradable, disulfide-containing antimicrobial polymers have been developed by rROP.^132^ The strategy was based on the copolymerization of benzyl lipoate with various monomers (OEGMA, hydroxyethyl acrylamide, and *tert*-butyl (2-acrylamidoethyl) carbamate), to produce a library of copolymers with demonstrated antimicrobial activity against drug-resistant *Pseudomonas aeruginosa*, improved hemocompatibility, and redox-responsive degradability.

In the field of tissue engineering, the design of advanced degradable scaffold is of great interest. In this context, Le Droumaguet et al.^235^ have conceived degradable biporous PCL-like networks based on the rROP of MDO with divinyl adipate via a double porogen templating approach. The two distinct levels of porosity enabled the materials obtained to have high compressibility and shape memory behavior during consecutive compression cycles, and pH-controlled degradation. This synthetic strategy was further applied to the design of functional biporous scaffolds via terpolymerization with 2-chloroethyl vinyl ether or azidoethyl vinyl ether for subsequent nucleophilic substitution or CuAAC reaction, respectively.^236^

### Degradable Water-Soluble Polymers

Degradable, water-soluble polymers are widely used in the biomedical, agricultural, and waste-treatment fields. Making them degradable is a major challenge in order to address the safety and environmental issues associated with their use. Along these lines, Thoniyot et al.^237^ developed biodegradable acrylic acid–based copolymers by copolymerizing MDO with *tert*-butyl acrylate, which temporarily protects the acidic functionalities. Subsequent acid hydrolysis regenerates the carboxylic acid groups and introduces cleavable ester bonds into the polymer chains (Figure 51). These copolymers exhibited chemical degradation but also biodegradability under environmental conditions (∼30% biodegradability after 1 month), demonstrating their potential as biodegradable thickeners or superabsorbents. Similarly, copolymerization of *t*BA with MDO was carried out comparing different polymerization methods (bulk versus solution, batch versus semibatch)^238^ across a broad feed composition range at different temperatures. Optimal polymerization (i.e., solution polymerization at 100 °C with *tert*-butyl peroxide as the initiator) and deprotection (i.e., 5 equiv of trifluoroacetic acid in DCM) conditions produced high ring-opening efficiency (>70%), pH and temperature dependent solubility, and significant degradation under accelerated conditions, leading to low *M*_n_ oligomers, suitable for microbial assimilation.

**Figure 51:**
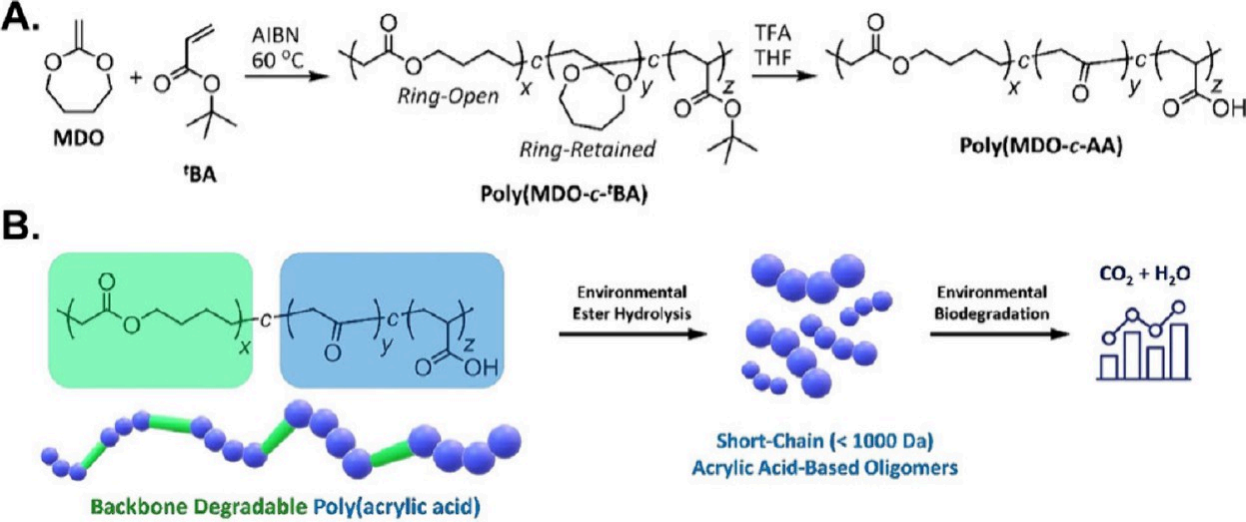
(A) rROP of MDO and *t*BA yielding poly­(MDO-*co*-tBA), followed by acid-mediated *tert*-butyl deprotection to obtain degradable poly­(MDO-*co*-AA). (B) Overview of biodegradability, through initial hydrolysis of the main-chain esters into short oligomers, followed by complete biodegradation. Reproduced from ref 237 with permission. Copyright 2022 Elsevier.

By exploiting the strong hydrophilicity of acrylamide (AAm) and establishing a structural analogy with S/AAm copolymers that exhibit upper critical solution temperature (UCST) transitions, Nicolas et al.^239^ copolymerized by RAFT AAm with aromatic ring-containing CKAs such as BMDO and MPDL. They successfully obtained UCST copolymers containing up to 13 mol % BMDO, capable of degrading in less than 24–72 h under physiological conditions (PBS, 37 °C, pH 7.4) Figure 52A, surpassing the degradation rates of all previous rROP-synthesized materials, as well as conventional polyesters such as PLA or PLGA (Figure 52B). Their in vitro enzymatic degradation in the presence of lipase and their high cytocompatibility on various healthy cell lines confirmed their suitability for biomedical applications. Copolymerization of AAm with MDO was also carried out by FRP, producing poorly defined structures with PMDO branches that also conferred UCST properties, but prevented complete solubility of the copolymers in water.^240^ In the context of chemotherapy, water-soluble Gem-poly­(AAm-*co*-BMDO) copolymer prodrugs synthesized by RAFT-mediated rROP were injected subcutaneously into mice and showed no local or systemic toxicity, as well as *in vivo* efficacy similar to that of Gemzar (the commercial formulation of Gem) injected intravenously.^231^

**Figure 52:**
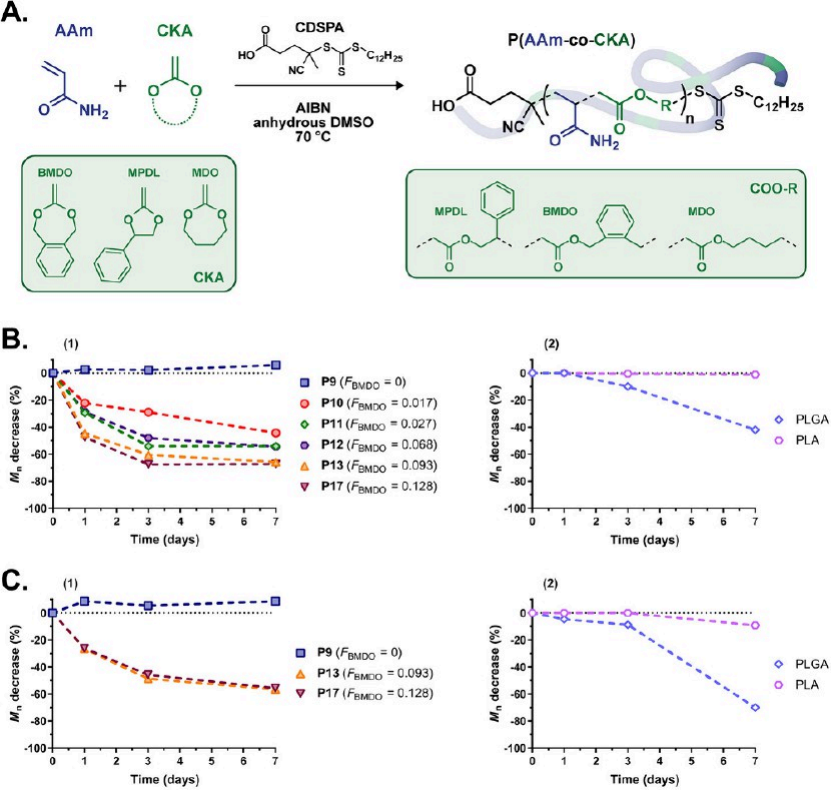
(A) Synthesis of P­(AAm-*co*-BMDO). (B) Evolution of the *M*_n_ with time during hydrolytic degradation in physiological conditions (PBS, pH 7.4, *T* = 37 °C) of (1) PAAm (P9), P­(AAm-*co*-BMDO) copolymers with different BMDO contents (P10–P13 and P17) and (2) PLA and PLGA. (C) Evolution of the *M*_n_ with time during enzymatic degradation with lipases (*Candida antartic*a, PBS, pH 7.4, *T* = 37 °C) of (1) PAAm (P9), P­(AAm-*co*-BMDO) copolymers P13 and P17 and (2) PLA and PLGA. Reproduced from ref 239 with permission. Copyright 2022 Springer Nature.

Maeda et al.^241^ reported the copolymerization of MDO and 2-hydroxyethyl vinyl ether (HEVE) or di­(ethylene glycol) vinyl ether (DEGV) to produce degradable copolymers with adjustable thermoresponsiveness. P­(HEVE-*co*-MDO) and P­(DEGV-*co*-MDO) copolymers exhibited LCST behavior in aqueous solutions for MDO contents in the 23–28 mol % and 35–37 mol % range, respectively. Roth et al.^126^ introduced thionolactones such as DOT into various polyacrylamides (neutral, zwitterionic, etc.) to create copolymers containing degradable thioester linkages. These materials retain tunable thermosensitive properties (LCST or UCST behavior) while being fragmentable under physiological conditions via hydrolysis, aminolysis, or transthioesterification. The solubility of the degradation fragments highlights their potential for bioeliminable or stimuli-responsive systems.

More recently, Nicolas et al.^242^ synthesized water-soluble biodegradable copolymers based on AAm and *N*-isopropylacrylamide (NIPAAm) by copolymerizing these monomers with DOT via controlled radical polymerization (RAFT) or free radical polymerization (FRP).

The resulting poly­(AAm-*co*-DOT) copolymers exhibited good molecular weight control, low dispersity, and rapid degradation in the presence of bases (NaOH), amines (isopropylamine), physiological amino acids (l-cysteine), or even commercial reagents (bleach), with up to 90% molecular weight loss in just 2 h. UCST transitions were also observed in saline media, depending on molecular weight and DOT content, illustrating their potential as biodegradable alternatives to conventional hydrophilic polymers for biomedical, agricultural, and environmental applications.^242^

Recently, access to advanced degradable and water-soluble architectures such as bottle brushes (BB) has been reported through RAFT copolymerization of αLA with acrylate-based inimers.^243^ These copolymers exhibited degradable polydisulfide backbones and side initiating sites for subsequent BB synthesis by ATRP of tri­(ethylene glycol) methyl ether acrylate, or monomers bearing cationic, anionic, and zwitterionic side chains. The BB polymers were successfully degraded under thiol-reducing conditions using dithiothreitol (DTT) and 2-TCEP.

The direct polymerization of CKAs in water is notoriously difficult due to their high sensitivity to hydrolysis and protic solvents. Nonetheless, the hydrolysis mechanism of various CKA monomers (from 5- to 8-membered rings) has been investigated during polymerization in water under organic solvent-free and surfactant-free conditions. CKAs rapidly hydrolyzes in water, particularly under acidic circumstances, yielding monoacetylated diol compounds. For example, it required in water at pH = 10 and at 70 °C only 30 min to reach 100% degradation for MDO.^50^ The hydrolysis rate is a multifaceted function of ring size, hydrophobicity, and the pH of the aqueous phase. MDO in pure water hydrolyzed more rapidly than the 8-membered CKA counterpart. Thoniyot et al.^50^ thus analyzed the hydrolysis kinetics of MDO and an 8-membered CKA in a homogeneous DMF–water combination at pH 10 and 30 °C. Both monomers underwent hydrolysis almost seven times more slowly in DMF–water compared to pure water, so confirming the significant influence of water solubility. The accelerated hydrolysis of the more hydrophobic 8-membered CKA compared to MDO in the homogeneous environment suggests that reduced solubility, rather than ring size, predominantly constrains hydrolysis in heterogeneous circumstances.^50^ The same authors investigated the hydrolysis mechanism via deuterium and ^17^O water labeling and DFT calculations.^50^ The proposed mechanism is depicted in Figure 53. The reaction mechanisms by routes a and b, under both acidic and basic conditions, yielded the same 4-(hydroxy)-butyl acetate product, incorporating ^17^O at the carbonyl ester group through the ring-opening of the H_2_^17^O substituted intermediate INT2 (Figure 53).

**Figure 53:**
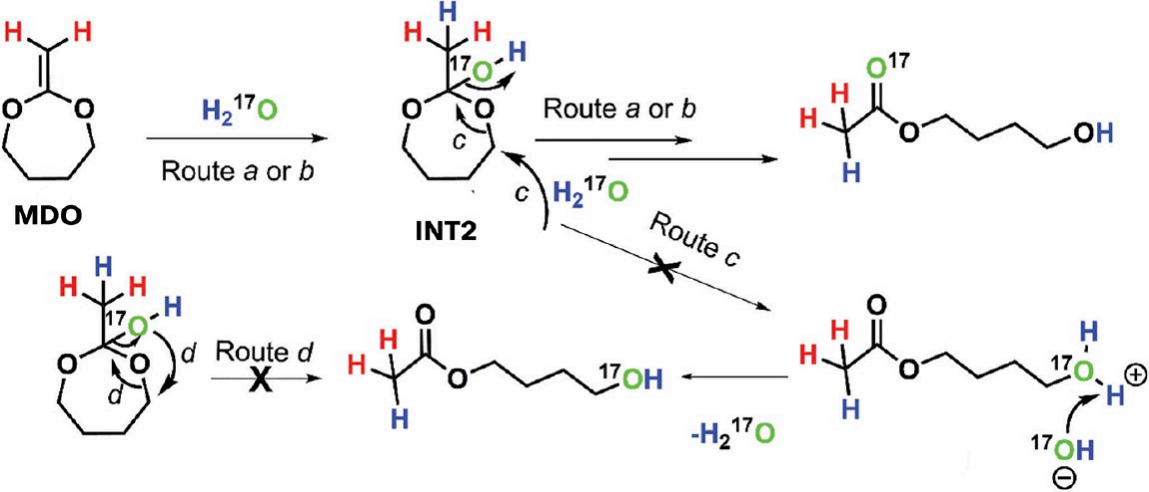
Proposed reaction pathway for a ^17^O-labeled experiment for the hydrolysis of MDO with H_2_^17^O at pH 2. Reproduced from ref 50 with permission. Copyright 2023 Wiley-VCH.

Moreover, the calculated energy barrier for base-catalyzed hydrolysis was determined to be greater (39.4 kJ mol^–1^) than that for acid-catalyzed hydrolysis (13.4 kJ mol^–1^), confirming the experimental data. It was finally shown that, although using basic reaction conditions resulted in some control over the hydrolysis rate of CKA, achieving controlled polymerization in water while suppressing hydrolysis remained challenging, resulting in only 3 mol % MDO and 8-membered CKAs in polyacrylate and polyacrylamide backbones.^50^ Agarwal and Melchin et al.^190^ did a similar study and determined kinetic rate constants for such reactions at a different temperature and pH. They proposed an autocatalytic mechanism due to the 4-hydroxy-1-butylacetate.

### Degradable Adhesive

Albanese et al.^130^ designed degradable pressure-sensitive adhesives (PSAs) by partially replacing acrylic acid with α-lipoic acid and *n*-butyl acrylate with ethyl lipoate (see the recycling part and Figure 30 for details). As mentioned above, the degradability of these materials relies on lipoic acid (or lipoate) diads along the backbone, the number of which can be controlled through the temperature and absolute concentration of the polymerization reaction. As is typical with acrylic-acid-based PSAs, mild cross-linking with Al­(acac)_3_ was found to improve the cohesive of the resulting films. Overall, the performance of these degradable PSAs was comparable to conventional, nondegradable formulations, demonstrating opportunities in using bioderived building blocks to create advanced materials.

At essentially the same time, Roth and co-workers^244^ described the use of thionolactone DOT to create degradable pressure-sensitive adhesives. A series of copolymers was then synthesized using radical copolymerization of *n*-butyl acrylate, 4-acryloyloxy benzophenone (ABP) as a photo-cross-linker, and DOT. The ideal tack and peel strengths were identified for molar concentrations of 0.05 mol % ABP and 0.25 mol % DOT. As described in detail above, DOT-based copolymers are degradable under different and complementary conditions as those containing disulfide bonds, for example, via aminolysis and thiolysis (Figure 54).

**Figure 54:**
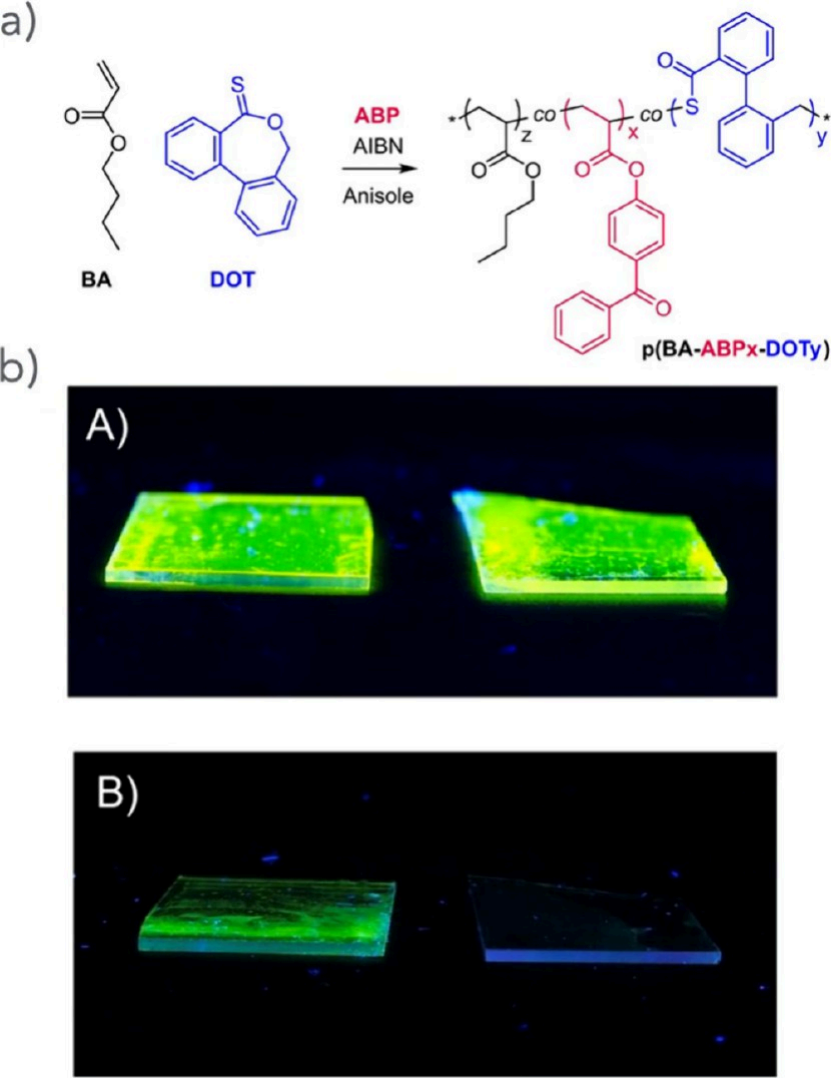
(a) Synthesis of UV cross-linkable degradable thioester-functional PSA. (c) Photos of dye-labeled photo-cross-linked copolymer films on glass substrates of the nondegradable control BA-ABP0.05-NBDA0.25 (left) and degradable BA-ABP0.05-DOT0.25-NBDA0.25 (right) A) before immersion and B) after immersion in 2 M *n*-propylamine in THF for 120 s, confirming visually the presence of insoluble residue for the control sample only. Reproduced from ref 244 with permission. Copyright 2023 Wiley-VCH.

Recent work has further extended this type of molecular design space to include cyclic allyl sulfides as comonomers to create polymers that degrade via intramolecular transesterification under basic conditions.^245^ Together, these papers on degradable PSAs create opportunities to design new adhesives that may overcome contemporary societal challenges, such as the buildup of persistent adhesive residue in the environment and clogging of recycling equipment with so-called “stickies.”

Building on this work, Messersmith and co-workers^246,247^ have reported a new family of polymeric adhesives derived from α-lipoic acid with all-disulfide backbones yet improved stability compared to typical dithiolane homopolymers by introducing an activated-ester-based monomer that prevents depolymerization during storage and use. These materials are promising in a variety of applications that necessitate mechanical properties ranging from soft and biocompatible to strong and structural in nature.

Medical adhesives have also been designed from cyclic ketene acetals. Agarwal et al.^248^ developed a biodegradable biomimetic adhesive based on polyester and catechol chemistry, inspired by mussel adhesive proteins (DOPA). Their MDO–GMA–OEGMA copolymer was functionalized with catechol groups and cross-linked using H_2_O_2_ or Fe­(acac)_3_.

The adhesive demonstrated good performance on fresh porcine skin, thermal stability, and enzymatic degradation over 30 days (i.e., 50% mass loss). Similarly, Xiao et al.^249^ designed adhesive hydrogels combining catechol-modified gelatin and a BMDO–maleimide copolymer bearing quaternary ammonium groups. This semi-interpenetrating network exhibited excellent adhesive, self-healing, antibacterial, and cytocompatible properties. Xiong and co-workers^250^ created underwater adhesives that are similar in spirit from MDO and a protected catechol-derivative: *N*-(3,4-dihydroxyphenethyl) methacrylamide (DMA). Luan and co-workers^251^ also developed tissue adhesives by *in situ* copolymerizing MDO with hydroxyethyl (meth)­acrylate using a red/ox initiating system (Figure 55). The adhesive is unaffected by environmental influences such as water. Ultrastrong adhesion (e.g., wet bone >16 MPa, and porcine skin >150 kPa) is attained using a backbone-degradable covalent interpenetrating network that solidifies throughout a broad time frame of seconds to hours. Ex-vivo and In-vivo experiments confirmed the efficiency of these adhesives. They furthermore showed that these adhesives enhance the incorporation of biomedical materials/devices onto the surfaces of diverse biological tissues. All of these examples have exciting potential in various medical applications, including surgical glues, hemostatics, and smart wound dressings. Collectively, these studies on adhesives highlight a promising convergence of polymer chemistry, dynamic materials, and biomimicry to meet the increasing demands for sustainability, recyclability, and high performance in both industrial and biomedical contexts.

**Figure 55:**
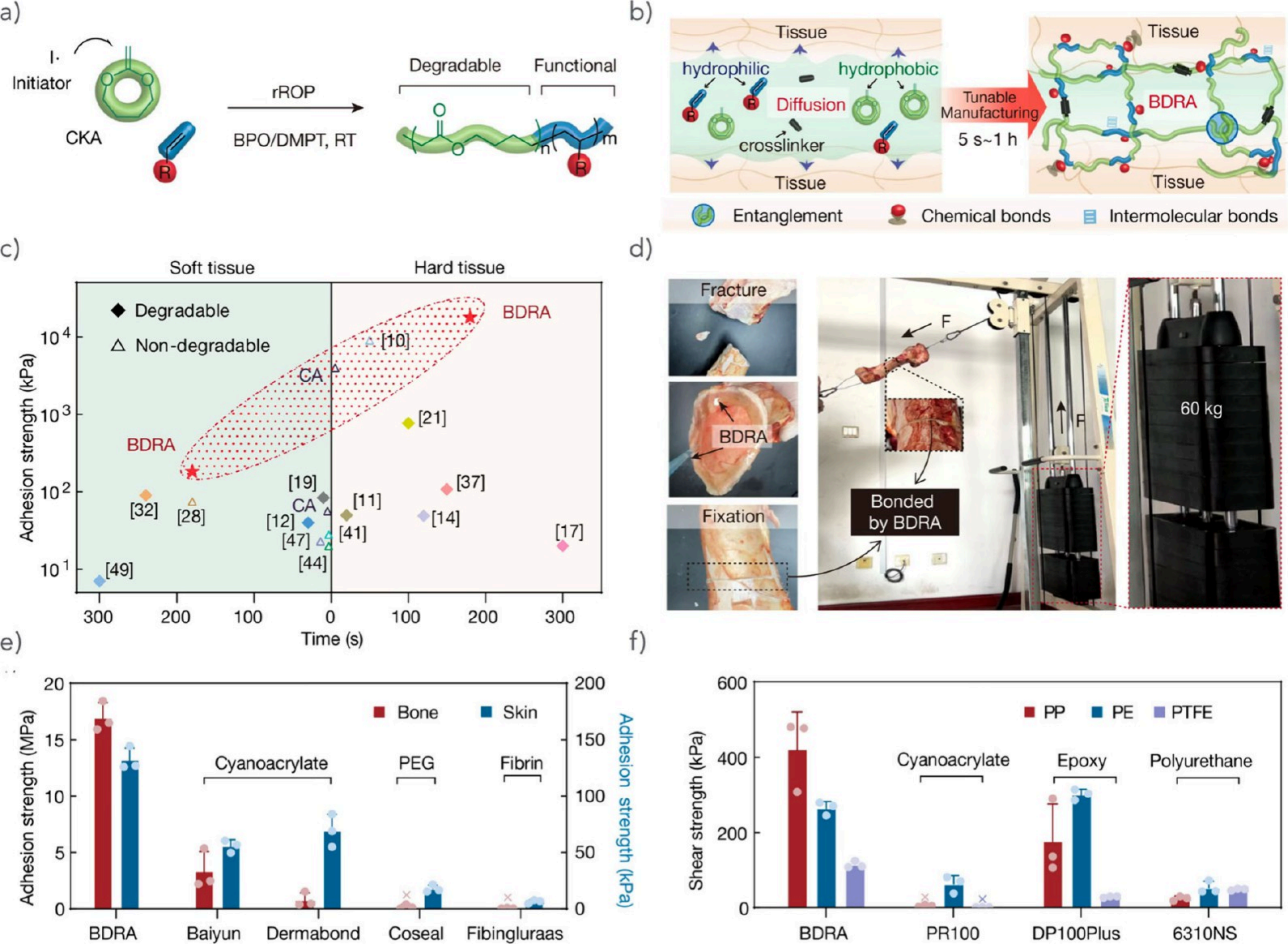
(a) In situ rROP of CKA and comonomers to form a degradable and functional macromolecular chain by redox initiation benzoyl peroxide/*N,N*-dimethyl-p-toluidine (BPO/DMPT). (b) Tunable preparation of the adhesive called backbone-degradable robust adhesives (BDRAs) that achieve strong adhesion by forming a covalent interpenetrating network by in situ rROP and the synergy of intermolecular and chemical bonds. (c) Adhesion strength and setting time of BDRAs and the existing tissue adhesives for hard and soft tissues. (d) Bearing capacity of bonded fractured bovine bone using BDRAs. (e) Adhesion strength of BDRAs and commercial medical adhesives on different biological tissues, represented by flexural strength for bone and shear strength for pigskin. Data are presented as the means ± SDs, *n* = 3 independent samples per group. (f) Shear adhesion strength for low-surface-energy polymers adhered by a BDRA and commercial engineering adhesives. PP polypropylene, PE polyethylene, PTFE polytetrafluoroethylene. Data are presented as the means ± SDs, *n* = 3 independent samples. Reproduced from ref 251 with permission. Copyright 2023 Springer Nature.

### Degradable Polyethylene

Polyethylene (PE), with over 130 million tons produced in 2021, remains the most industrially produced polymer, whether through radical or coordination–insertion polymerization. Its popularity stems from its intrinsic chemical inertness and thermal stability, resulting from its ability to crystallize and lack of functionality. In the context of plastic end-of-life and the growing emphasis on recyclability and biodegradability, it becomes crucial to explore methods to enhance these desirable properties while preserving PE’s thermal stability. rROP offers a promising approach to achieve this goal. Ethylene being the most representative of less activated monomers, MDO naturally presents as a highly pertinent candidate for radical copolymerization. Indeed, in their seminal works on rROP of MDO, Bailey et al. very early described the possibility to copolymerize 5 to 22% of MDO with ethylene at 120 °C and the incorporation of 2 to 10% of ester units in the final copolymer.^252^ Biodegradability studies showed that rapid degradation occurred for chains containing ca. 10 mol % of MDO.^252,253^

You et al.^254^ recently studied the copolymerization of ethylene and MDO in dimethyl carbonate (DMC) at moderate temperatures. Degradable LDPE with ester linkages and a molar mass of about 12,000 g·mol^–1^ were synthesized using cobalt-mediated radical polymerization (CoMRP), under conditions adapted from the ones depicted to ensure a good control of the polymerization. By adjusting the ethylene pressure and the amount of MDO, diverse P­(E-*co*-MDO) copolymers were synthesized. The degradation of the copolymer comprising 68% of ethylene and a molar mass of 11,200 g·mol^–1^ was examined. After 24 h at 70 °C in a triethylamine-chloroform solution (1/4 v/v), a notable reduction in the molar mass of the chains (*M*_n_ of 670 g·mol^–1^ postdegradation) was observed using high temperature size exclusion chromatography (HT-SEC), confirming the degradability of the original LDPE chains. The same authors subsequently studied the RAFT copolymerization of CKA and ethylene mediated by a dithiocarbamate (RAFT).^255^ While the copolymerizations were conducted with small reaction volumes, degradable PE chains were claimed particularly in the presence of lipases.^256^

D’Agosto, Destarac, and collaborators recently studied the first radical ring opening copolymerization of ethylene with TCL (Figure 56). The copolymerizations were initiated by AIBN and carried out in a 160 mL autoclave containing 50 mL of DMC at 70 °C under 80 bar of ethylene.^178^ In batch mode, TCL conversion was monitored by ^1^H NMR. Complete TCL consumption was observed after 180 min, after which ethylene consumption only really started, leading to a strong compositional drift and the formation of mostly nondegradable polyethylene chains. To overcome this, copolymerization was performed in a semibatch mode by continuously feeding a TCL solution in DMC at various rates (0.04–0.2 mL·min^–1^).^178^ This approach enabled high conversion (>90%) and homogeneous TCL incorporation (1–7 mol %) in the chains with *M*_n_ between 3,100 and 6,600 g·mol^–1^ by HT-SEC analyses that confirmed monomodal distributions. Thermal analyses further showed that the introduction of TCL in the chains did not alter their thermal stability compared to PE chains obtained under the same polymerization conditions (Figure 56B). Chemical degradations conducted via aminolysis at 90 °C with allylamine validated chain cleavage of the thioester functions, which was not observed for pure polyethylene, as expected.

**Figure 56:**
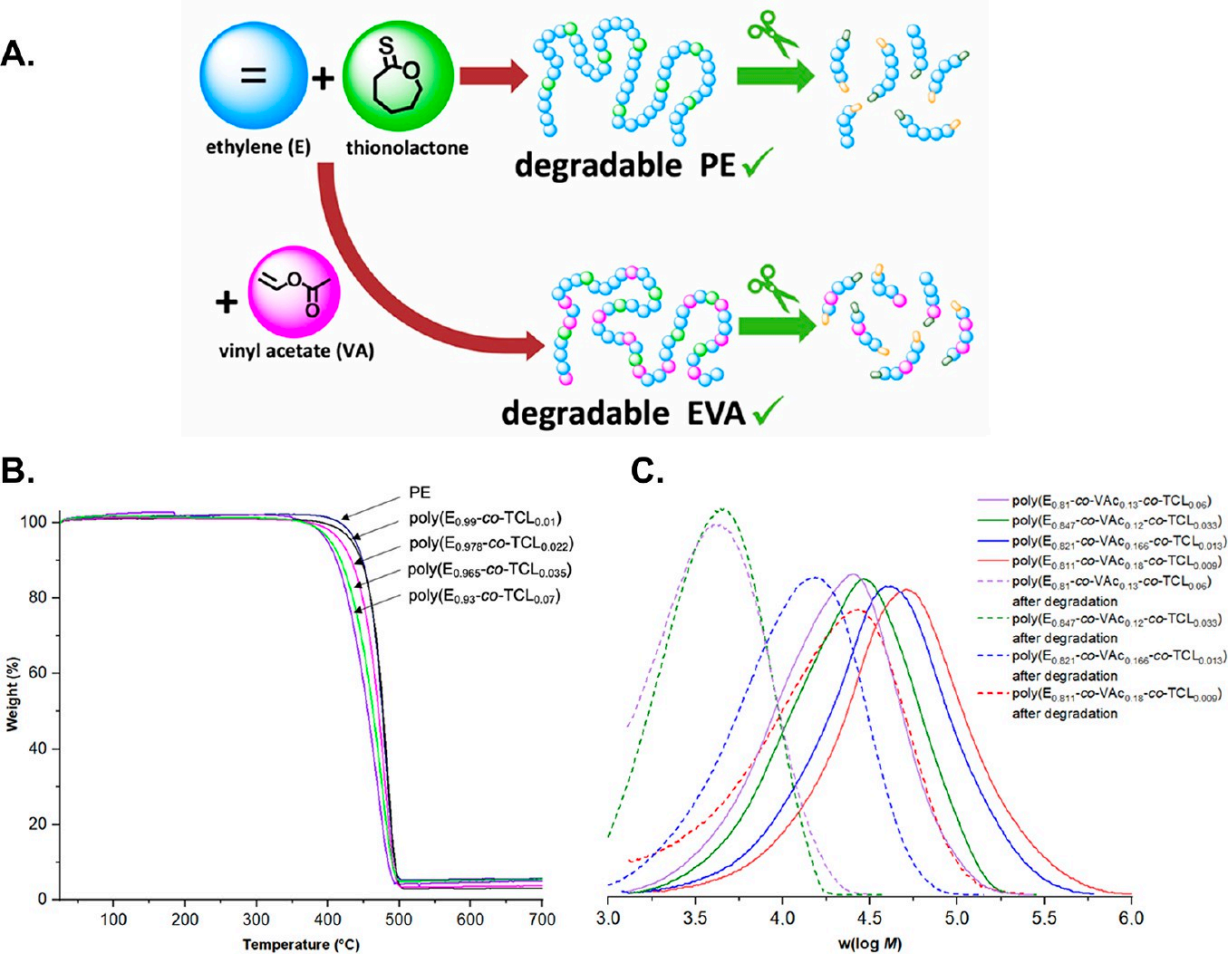
(A) rROP of ethylene, vinyl acetate and thionolactone for the production of chemically degradable PE and EVA. (B) Thermogravimetric analyses of P­(E-*co*-TCL). (C) SEC analyses of pristine and degraded (dashed lines) P­(E-*co*-VAc-*co*-TCL). Adapted from ref 178 with permission. Copyright 2024 American Chemical Society.

These results were extended to semibatch terpolymerizations of ethylene, VAc, and TCL for which a controlled TCL incorporation ranging between 0.9 and 6 mol % was shown and molar masses reaching 29 kg·mol^–1^. Degradation in the presence of TBD in THF showed a significant reduction in molar mass (Figure 56C), confirming the effectiveness of this strategy for producing chemically degradable PE- or EVA-like materials.^178^

### Miscellaneous

Besides classic degradation conditions, some authors investigated the use of a specific trigger in order to obtain main chain degradation. In this topic, Kohsaka^257^ and co-workers for example prepared a terpolymer in which the CKA BMDO was inserted to confer degradability but in which a specific silylated methacrylate-based third monomer was also added to confer latent triggering. This latter compound is designed to polymerized in a alternating sequence with BMDO to led after specific deprotection of the alcohol group to an intramolecular cyclization, inducing the cleavage of the backbone (Figure 57). Copolymer of MMA, BMDO, and methyl 2-(trimethylsiloxymethyl)­acrylate (SiO-MMA) with MMA content between 25 and 78 mol % led to copolymers that could be degraded via tetrabutylammonium fluoride (TBAF) treatment, showing an important decrease of the *M*_n_.

**Figure 57:**
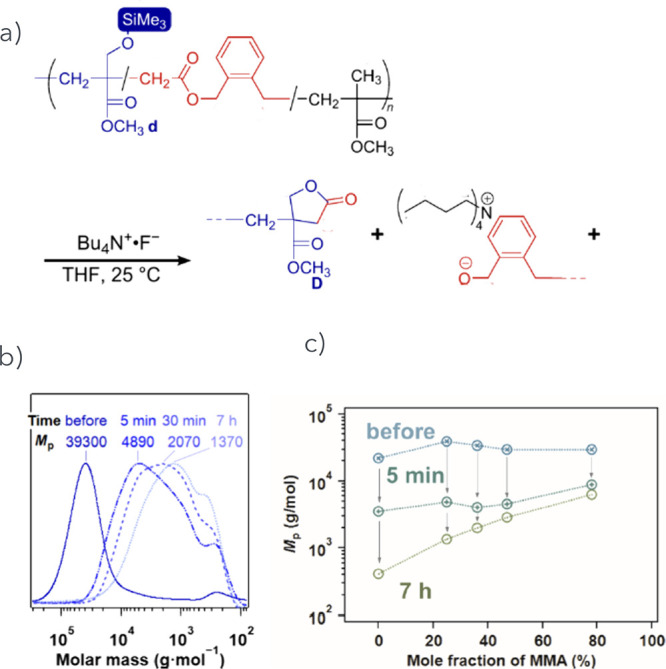
a) Degradation of a PMMA-based copolymer via a specific TBAF triggering. b) SEC of pristine and degraded copolymer ([MMA]:[BMDO]:[SiOMMA] = 25:18:57, *M*_n_ = 31,100 g·mol^–1^, *D* = 1.54). c) Evolution of *M*_p_ with an increasing of amount of MMA in the copolymer. Reproduced from ref 257 with permission. Copyright 2024 American Chemical Society.

Extrapolation to styrene was successful, whereas in the case of acrylate derivatives no degradation was observed. In addition to specific triggers such as (TBAF), dual stimuli responsive materials have also been envisioned. Xiao^258^ and co-workers for example prepared some copolymers combining BMDO as a degradable backbone with the photolabile monomer *o*-nitrobenzyl methacrylate (NBM). They obtained dual-degradable copolymers able to undergo the decomposition of the backbone under an accelerated alkaline condition and the collapse of pendants by UV light irradiation.

In a different manner, Frisch et al.^259^ inserted a photolabile moiety into the polymer backbone by copolymerizing methyl acrylate and/or *N,N*-dimethyl acrylamide vis an SCM monomer bearing a photosensitive group. They prepared for example the SCM11 monomer (Figure 10) by an intramolecular [2 + 2] photocycloaddition of a precursor having two coumarin moieties. The subsequent RAFT-mediated rROP with methyl acrylate produced copolymers that retained the photoreactivity of the cyclic parent monomer. UVB irradiation effectively triggered the photocycloreversion of coumarin dimers, resulting in polymer disintegration within minutes under UVB light or days under sunshine exposure.^65^ In a second study,^259^ the same authors used a different [2 + 2] photocycloaddition reaction between styryl pyrene to prepare block copolymers complementary photoreactive cyclic comonomers. By using the varying absorbances of photoreactive cyclic monomers, selective degradation of blocks may be achieved by irradiation with either UVB or UVA light (Figure 58a). The hydrophobicity of the photodegradable monomers facilitated the translation of primary polymer sequences into higher-order assemblies, resulting in the creation of micelles in water. Upon exposure to light, the nondegradable blocks disassociated, resulting in a substantial decrease in the micelle hydrodynamic diameter.

**Figure 58:**
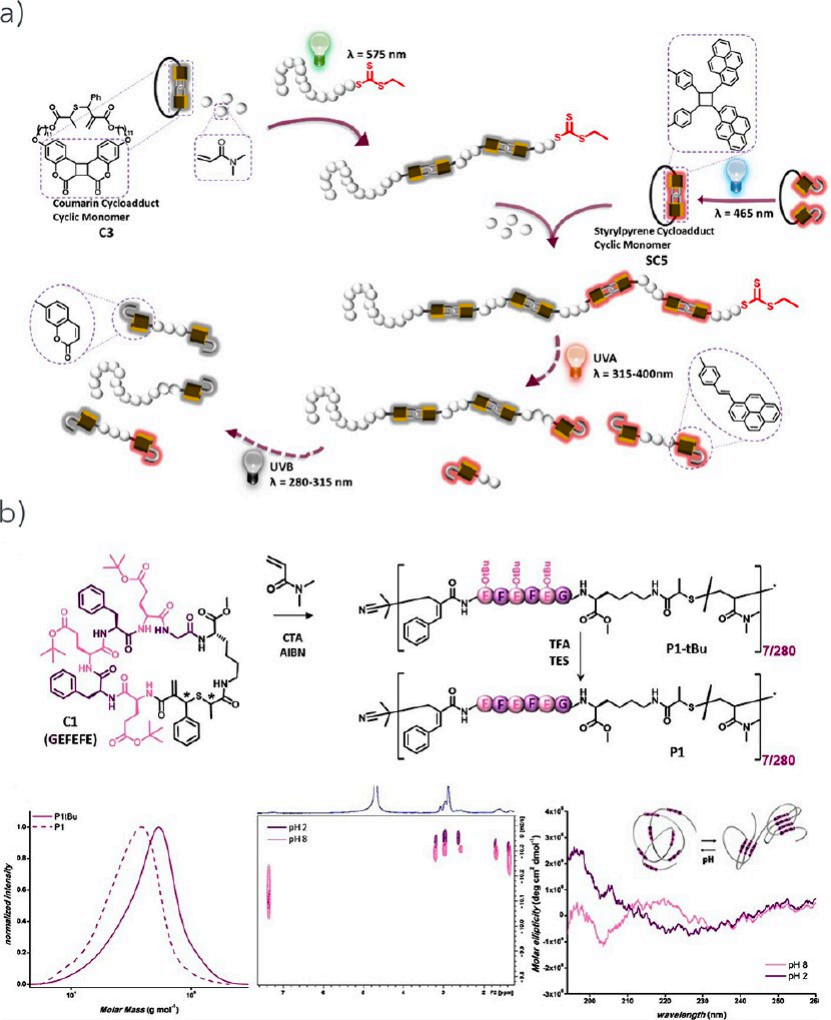
(a) Triblock copolymer synthesis and its stepwise photodegradation: a diblock copolymer consisting of a PDMA nondegradable block and photodegradable copolymer of the coumarin cycloadduct and DMA block is prepared by green light-initiated RAFT polymerization. Chain extension of this polymer with RAFT copolymerization of DMA and the cyclic monomer resulted from intramolecular [2 + 2] cycloaddition of styrylpyrene under blue light yields a triblock polymer with the copolymer of DMA and styrylpyrene cycloadduct as the third block. Under UVA, the styrylpyrene cycloadduct experiences [2 + 2] cycloreversion, leading to the fragmentation of the third block. Subsequent UVB irradiation initiates the degradation of the second block as the coumarin dimer in the polymer backbone undergoes [2 + 2] cycloreversion. Reproduced from ref 259 with permission. Copyright 2023 Wiley-VCH. (b) Synthesis of a polypeptide mimic and its SEC traces before (P1-tBu) and after (P1) deprotection. DOSY NMR of P1 at a 1 mg/mL concentration and CD spectra of P1 (0.2 mg/mL) at basic (pink) and acidic (purple) pH. The CD traces have not been smoothed. Reproduced from ref 260 with permission. Copyright 2024 Wiley-VCH.

Functional peptide sequences can only be included as side chains or chain termini in all chain growth polymerization schemes, thereby neglecting the main chain’s contributions. However, in nature, a peptide’s fundamental sequence, or main chain, mostly controls its function. There are currently few synthetic methods available to incorporate specific peptide sequences into the main chain of polymers. Thus, Frisch et al.,^64^ using a similar approach as the one described above, created a solid phase synthesis pathway to the precursors of linear peptide allylic sulfides. The allylic sulfide is produced on resin at the N-terminus of the solid support-bound peptide in a three-step process after the synthesis of the required peptide sequence using conventional Fmoc-based SPPS. When the N-terminus is deprotected, the side-chain-protected linear allylic sulfide cleaves off the solid support and undergoes cyclization via head-to-tail amidation (SCM12, Figure 10). The copolymerization of such monomers with *N,N*-dimethylacrylamide was performed confirming the preparation of peptide mimics containing up to 15 peptide sequences per polymer chain.^64^

β-Sheet-encoded peptides have been documented to facilitate supramolecular pH-controlled self-assembly by alternating hydrophobic amino acids with weakly acidic or basic amino acids, as their self-assembly capability can be modified by protonation or deprotonation in response to pH variations. Frisch and colleagues^260^ evaluated the transposition of this feature onto synthetic polymers through a strategy that involved incorporating sequence-defined β-sheet encoded hexapeptides, composed of alternating weakly acidic or basic and hydrophobic amino acids, into the vinyl polymer backbone via rROP of peptide-containing macrocycles, subsequently utilizing pH variations to facilitate intramacromolecular peptide self-assembly and chain folding in a completely reversible fashion. The preparation of copolymers containing SCM monomers with either peptide sequence featuring alternating hydrophobic and acidic amino acids of phenylalanine (F) and glutamic acid (E, full sequence: GEFEFE) or after substitution of glutamic acid to lysine K (full sequence GKFKFK) led to β-sheet structures that could be erased using a pH change (Figure 58b).

## (BIO)Degradation

### Homopolymerization

Homopolymers derived from CKA monomers such as MDO, BMDO, and MPDL were synthesized and characterized as early as the 1980s. Their molecular structures confirm the regular incorporation of ester linkages into the polymer backbone, thereby imparting hydrolytic functionality to the material. The mode of degradation is thus similar to the one of common polyester such as polycaprolactone and polylactides.^261,262^ In the case of MDO, homopolymerization leads to an amorphous polyester, known as branched PCL (PCLB), whose branched architecture prevents crystallization. This structural feature enhances water diffusion and improves ester accessibility for hydrolysis in the case of solid films. Agarwal and Speyerer^86^ demonstrated that a blend of PCL and PCLB underwent faster degradation under industrial composting conditions, thus confirming the material’s biodegradability. Within 11 days, materials containing at least 40% PCLB fully degraded, whereas the blend with only 20% PCLB degraded after 15 days. More recently, Malmström et al.^263^ synthesized PCLB exhibiting branching degrees between 8% and 18%, with molar masses ranging from 22,000 to 41,000 g·mol^–1^ (*Đ* = 1.5–1.15).

An amorphous homopolymer of poly­(2-methylene-4-methyl-1,3-dioxepane) (PMe-MDO) was also prepared, showing a molar mass of 10,000 g·mol^–1^ (*Đ* = 1.7). The chemical degradation in solution of these polyesters was investigated by basic hydrolysis in THF using KOH (0.5 wt % in MeOH) for 1 to 5 h. Only little variations were found between PMDO or PCLB and PCL, where the distinction is in the degree of branching. The rate of hydrolysis was marginally reduced with an increased degree of branching, in comparison to PCL, which may be ascribed to steric hindrance surrounding the ester linkages in the main chain due to the branching (Figure 59a). The hydrolysis of PMe-MDO is considerably slower than that of PMDO and PCL, a phenomenon due to the steric hindrance caused by the methyl group in PMe-MDO, contrary to the degradation that was observed for solid materials. The same group^263^ also investigated biodegradability of the same for polyesters using OECD 301D protocol (Figure 59b). Biodegradability tests conducted using the Closed Bottle Test (CBT) with river water inoculum showed a mineralization of 66% for PMDO (whatever the degree of branching) after 56 days, compared to only 9% for PMe-MDO, indicating that the introduction of a methyl side group significantly slows down the degradation. The biodegradability of PCL was found to be a bit slower during the first week before reaching similar biodegradability than PMDO after 56 days (close to 60%).

**Figure 59:**
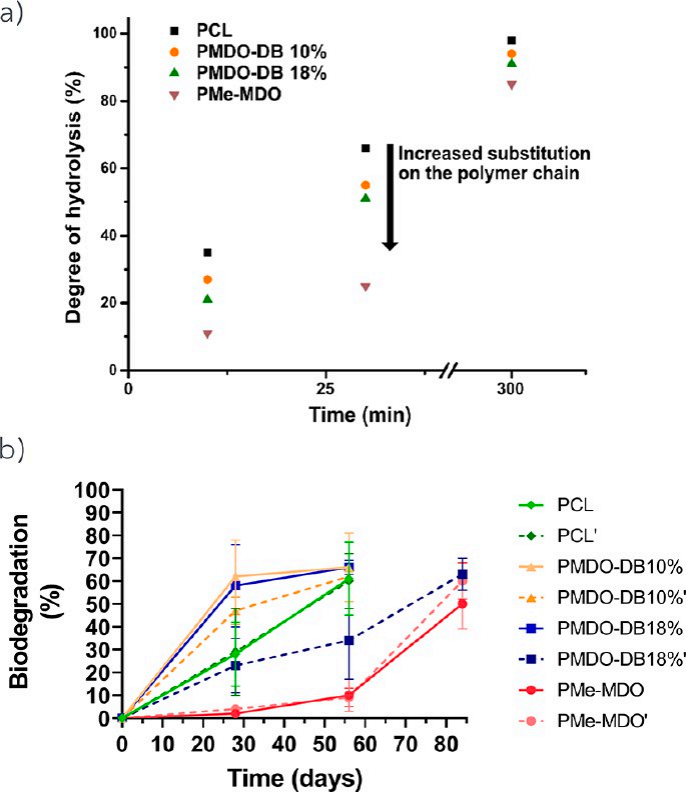
a) Degree of hydrolysis of PCL, PMDOs, and PMe-MDO as a function of time. b) Biodegradation test results of PCL, PMDO-DB 10%, PMDO-DB 18% and PMe-MDO in river water. Mean and standard deviations (SDs) are calculated based on the biodegradation achieved in three replicate bottles for two biological replicate per inoculum. Reproduced from ref 263 with permission. Copyright 2023 Royal Society of Chemistry.

Hiraguri and Tokiwa^264^ synthesized the homopolymer of 2-methylene-1,3,6-trioxocane (MTC) via radical solution polymerization, yielding an amorphous polyester-ether with a molar mass of *M*_n_ = 5,200 g·mol^–1^ (*Đ* = 5.56). Its enzymatic degradability was then assessed in phosphate buffer using lipase from *Rhizopus arrhizus*. After incubation at 30 °C for 16 h, 67% of the polymer became soluble, indicating efficient enzymatic degradation. This high solubilization is attributed to ester bond cleavage along the polymer chain, generating low-molecular-weight hydrophilic fragments. No degradation was observed in the absence of enzyme, confirming the enzymatic origin of the process.

In the context of chemical degradation, homopolymers derived from monosaccharide-based CKAs, as described in 2024 by Niu and co-workers,^57^ exhibit selective acid-triggered degradation or depolymerization that enables near-quantitative recovery of the derived lactone, that could be a building block of interest.

Concerning the homopolymer of SCM monomers, their degradation has been little investigated. Huang and Niu^35^ showed rapid and efficient degradation of polyester derived from SCM8 (Figure 10) after just 10 min of treatment at room temperature with sodium methanolate (MeONa) in a THF/MeOH mixture. Analysis of the degradation products by ^1^H NMR identified two main compounds, a dimethyl ester derivative and 1,4-butanediol, confirming the selective cleavage of ester bonds along the polymer chain.

The homopolymerization of thionolactones has been less reported. Roth et al.^97^ observed that DOT homopolymerizes with difficulty (<10% conversion after 7 days at 60 °C). Nevertheless, a more recent publication from the same group,^265^ dedicated to the cationic polymerization of DOT, proposed a detailed degradation mechanism for poly­(DOT). This mechanism involves several concurrent pathways: an α-depolymerization initiated from the chain end, an ω-depolymerization promoted by the presence of nucleophilic species such as ethanethiolate (EtS^–^), as well as a self-immolation process leading to the formation of the thiolactone DTO monomer, an isomer of the starting thionolactone (Figure 60). A similar thiolactone was also observed by Reineke and co-workers^100^ when poly­(TIC) was degraded (Figure 19). Other pathways, including intramolecular cyclization and thermal degradation, may also occur depending on the experimental conditions, illustrating the complexity of the depolymerization processes of poly­(thionolactones).

**Figure 60:**
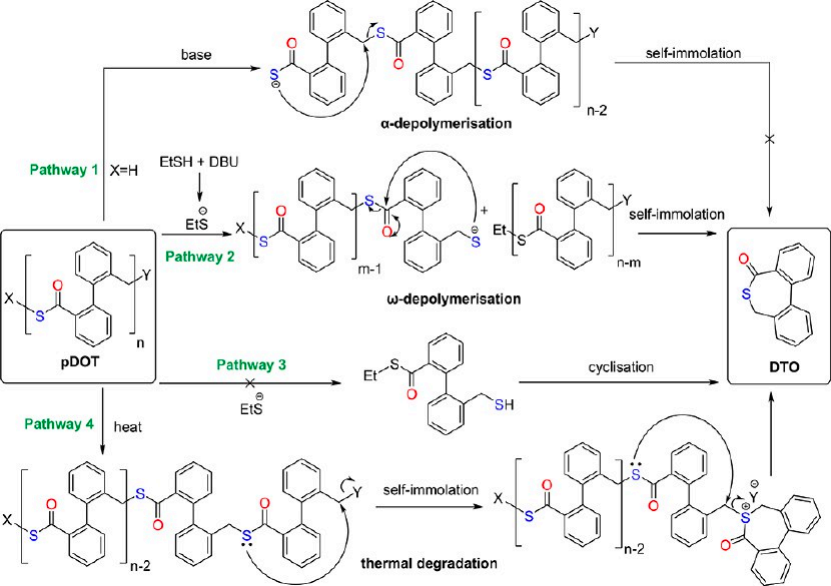
Proposed degradation pathways of a pDOT chain, where X = H and Me, and Y = OH and OTf for BF3·Et2O-initiated and MeOTf-initiated polymers, respectively. Reproduced from ref 265 with permission. Copyright 2024 Elsevier.

Recently Roth et al.^99^ proposed to use diethyl vinylphosphonate as a comonomer that enabled one to prepare radically a polymer containing mainly DOT units. These polymers proved to be highly degradable: aminolytic treatment yielded small bifunctional organic molecules bearing both thiol (−SH) and amide (−CONHR) groups, while thermal heating triggered complete depolymerization, regenerating the DTO monomer through a mechanism analogous to that previously described for the cationic depolymerization of P­(DOT). The degradation products were analyzed by size exclusion chromatography (SEC), confirming the almost complete disappearance of the initial polymer chains.

Homopolymers derived from ethyl lipoate exhibit particularly efficient degradation, both thermally and chemically. Raeisi and Tsarevsky^101^ demonstrated that simple exposure to 150 °C for 4 h induces significant thermal degradation, with the number-average molar mass decreasing from 64,000 g·mol^–1^ to 8,000 g·mol^–1^ in the absence of any added reagent. To confirm the involvement of a radical mechanism, the authors used AIBN as a radical initiator at 60 °C. Under these conditions, poly­(ethyl lipoate) with *M*_n_ = 45,000 g·mol^–1^ (*Đ* = 2.74) decreased to 1,800 g·mol^–1^ upon addition of 10 mol % AIBN, indicating high sensitivity to thermal radicals. Moreover, rapid and complete chemical degradation was achieved in solution at room temperature using tributylphosphine (Bu_3_P): with 0.5 equiv of Bu_3_P, the molar mass dropped from *M*_n_ = 49,000 g·mol^–1^ (*Đ* = 4.45) to 1,400 g·mol^–1^ (*Đ* = 1.47), and further to 800 g·mol^–1^ (*Đ* = 1.02) with 2 equiv, in just a few minutes. These results highlight the pronounced reactivity of lipoate-based homopolymers and their potential for controlled degradation via thermal, chemical, or radical triggers, depending on the desired application.

### Copolymerization

#### Polystyrene

Styrenic copolymers incorporating CKA units exhibit limited degradation potential due to a pronounced mismatch in reactivity ratios. Styrene is strongly favored during polymerization, leading to low CKA incorporation even at high initial feed ratios. Hiraguri et al.^264^ demonstrated that the incorporation of MTC into a styrene copolymer allows the introduction of hydrolyzable ester linkages. The copolymer P­(MTC-*co*-S), subjected to enzymatic degradation using *Rhizopus arrhizus* lipase (30 °C, pH 7), displayed a solubilization rate limited to 7% after 16 h, indicating partial degradation. This moderate efficiency contrasts with the performance observed for the MTC homopolymer, which reaches 67% solubilization under the same conditions, suggesting that the presence of styrene hinders the accessibility or reactivity of degradable segments.

The incorporation of MDO or BMDO into styrenic copolymers was investigated by the group of van Herk and Thoniyot.^110^ The insertion rate of these monomers varied with the initial monomer feed ratio, reaching up to 23 mol % for MDO and 18 mol % for BMDO. Upon mild alkaline hydrolysis (THF/MeOH/KOH), significant degradation of the copolymers was observed, as reflected by a notable decrease in molar mass. For instance, a P­(MDO-*co*-S) copolymer containing 23 mol % MDO exhibited a reduction in *M*_n_ from 9,800 g·mol^–1^ (*Đ* = 1.77) to 600 g·mol^–1^ (*Đ* = 2.17) after treatment. Similarly, a P­(BMDO-*co*-S) copolymer with 18 mol % BMDO showed a drop from *M*_n_ = 17,400 g·mol^–1^ (*Đ* = 3.83) to *M*_n_ = 1,900 g·mol^–1^ (*Đ* = 6.05). Notably, high-molar-mass copolymers can still be rendered degradable with low CKA content. A copolymer with only 2 mol % MDO underwent degradation from 80,300 g·mol^–1^ (*Đ* = 2.16) to 4,300 g·mol^–1^ (*Đ* = 2.93), while another containing 2 mol % BMDO degraded from 50,100 g·mol^–1^ (*Đ* = 2.53) to 4,600 g·mol^–1^ (*Đ* = 2.93). This degradation relies on the cleavage of ester linkages introduced by ring opening, yielding hydroxyl-terminated fragments either alcohols or phenols depending on the nature of the CKA and a carbonyl acid. Moreover, Thioniyot et al.^266^ also confirmed that the incorporation of ester into PS main chain, that led to degradable PS nanoparticles, do not induce more cytotoxicity or pro-inflammatory and anti-inflammatory biomarkers after subcutaneous injection than the reference PS analogue.

Guillaneuf et al.^111^ and Johnson et al.^78^ incorporated the DOT thionolactone into polystyrene chains via rROP, leading to copolymers containing up to 5% thioester units. The first degradation experiments by basic hydrolysis (5% KOH, 18 h, rt) showed a strong decrease in molar mass, from *M*_n_ = 75,000 g·mol^–1^ (*Đ* = 2.0) to 2,400 g·mol^–1^ (*Đ* = 2.6).^111^ A significantly faster degradation was observed in the presence of 1,5,7-triazabicyclo[4.4.0]­dec-5-ene (TBD), a particularly active organic base: in THF solution, the copolymer was completely degraded within 1 h.^111^ Furthermore, the degradation kinetics were monitored by in situ rheology using a Couette cell containing a 20 wt % copolymer solution. After addition of TBD (1.25 wt %), a rapid decrease in specific viscosity was observed, reaching a 90% reduction in less than 20 min, confirming the almost complete degradation of the copolymer under these conditions. Aminolysis using neat *n*-propylamine required more than 24 h to lead to complete deconstruction.^78^ Increasing the temperature to 55 °C (sealed tube) speeded up the complete decomposition in less than 5 h. Oxygen exclusion was essential in these degradation reactions to prevent ill-defined end-groups resulting from disulfide formation and subsequent unidentified reactions,^78^ Degradation was also performed using cysteamine in combination with DBU in THF at RT was also particularly efficient.^78^ Rather similar results were obtained by Guillaneuf et al.^111^ The use of *N*-isopropylamine in dichloromethane, as previously reported, did not result in the degradation of the copolymers within 18 h. Various amines with differing steric hindrance, such as ethanolamine in DCM and ammonia in THF, were tested at room temperature, but these attempts were unsuccessful. Likewise, they attempted the heterogeneous degradation of polystyrene in basic water, but this effort was also unsuccessful.

Finally, aminolysis was attempted under harsher conditions by increasing both the concentration and temperature. Complete degradation was achieved in pure *n*-butyl amine at 50 °C after 18 h, in good agreement with Johnson and co-workers.^78^ The presence of organic acids, including trifluoroacetic acid (TFA) or para-toluene sulfonic acid (PTSA), at a concentration of 1.25 wt % in THF did not result in significant degradation.^111^ Lastly, inspired by the work of Roth et al.^267^ and Destarac et al.,^74^ Guillaneuf and co-workers tried different oxidant reagents to degrade polystyrene chains containing the POT thionolactone.^69^ Oxone using the reported conditions did not lead to degradation and thus the authors focused on *meta*-chloroperbenzoic acid (*m*CPBA). PS copolymers underwent efficient degradation at room temperature over 17 h in a THF solution containing 2.5 mol % of *m*CPBA, while the polymers lacking thionolactone remained unaffected.^69^ Figure 61 summarizes various degradation conditions reported for PS containing thioester units.

**Figure 61:**
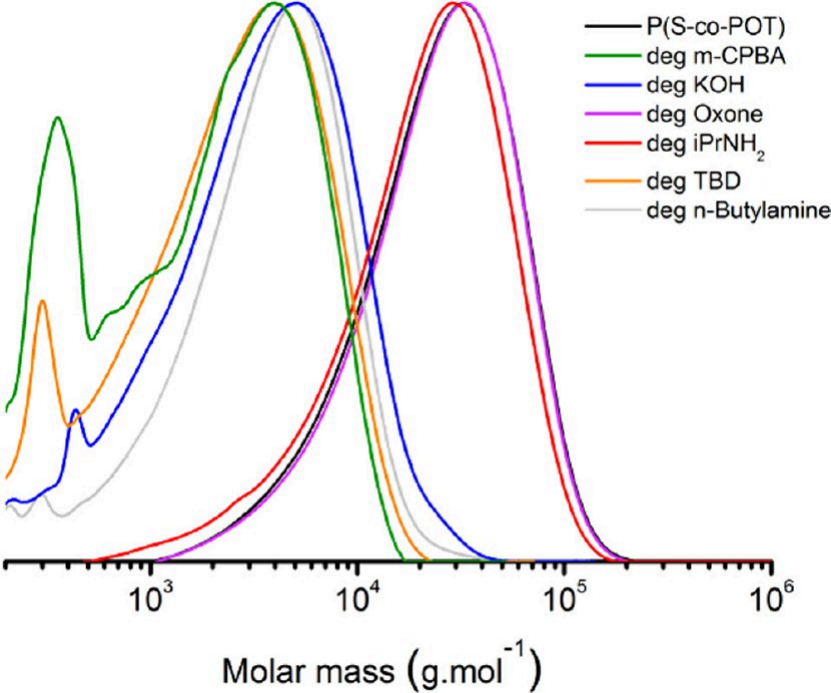
Evolution of the molar mass distribution of a P­(S-*co*-POT) prepared at 80 °C and 5% POT before and after various degradation conditions. Reproduced from ref 69 with permission. Copyright 2023 American Chemical Society.

As already discussed, the copolymerization of styrene and lipoate derivatives is less efficient than with acrylates, and the incorporation of diads to impart reductive degradation is difficult. Bates and Hawker et al.^115^ proposed a simplified approach aimed at improving the recyclability of styrenic copolymers by introducing very low proportions of units derived from α-lipoic acid (LA) or ethyl lipoate (ELp). The copolymers P­(S-*co*-LA) and P­(S-*co*-ELp), containing 0.4–3.7 mol % of lipoate units and up to 4.3 mol % of ethyl lipoate units, respectively, were obtained by radical solution polymerization.

These materials, with initial molar masses ranging from 12 to 40 kg·mol^–1^, underwent efficient degradation upon heating at 100 °C in DMF under air, with a decrease in *M*_n_ down to 3 kg·mol^–1^, resulting from the cleavage of S–S and C–S bonds within the polymer backbone.^115^ Two studies investigated the use of emulsion polymerization to prepare such copolymer. Komarneni and Huang et al.^116^ prepared a styrene-based copolymer containing disulfide linkages that was synthesized via emulsion polymerization at room temperature, using *tert*-butyl lipoate (t-BLp) as a comonomer.

The degradation behavior of the polymer was investigated through two distinct methods. The first involved a reductive treatment using dithiothreitol (DTT) at 80 °C in dioxane, leading to cleavage of the disulfide bonds and formation of polystyrene chains terminated with thiol groups. The second approach relied on UV irradiation at 365 nm under ambient conditions, which also yielded thiol-terminated oligomers. SEC analysis indicated efficient degradation, although significant fractions of undegraded polymer remained. The molecular weight decreased from 460,000 g·mol^–1^ (*Đ* = 2.97) to 70,000 and 90,000 g·mol^–1^ (*Đ* = 2.14 and 2.64), depending on the degradation condition.^116^ Zetterlund et al.^207^ demonstrated the direct incorporation of unmodified α-lipoic acid into polystyrene, via ab initio emulsion polymerization—a promising method for aqueous processing and industrial scalability. Contrary to bulk polymerization, the copolymers P­(S-*co*-LA), containing 10 mol % lipoate units and synthesized using VA-044 or VA-057 initiators in the presence of CTAB or SDS surfactants, exhibited initial molar masses between 33,000 and 37,000 g·mol^–1^. After treatment in DMF at 100 °C under air, the polymers underwent pronounced backbone degradation, with *M*_n_ decreasing to approximately 4,000 g·mol^–1^, corresponding to a degradation efficiency of nearly 90%.

The different experimental conditions for degradable styrenic -based copolymers are gathered in Table 5.

**Table 5:**
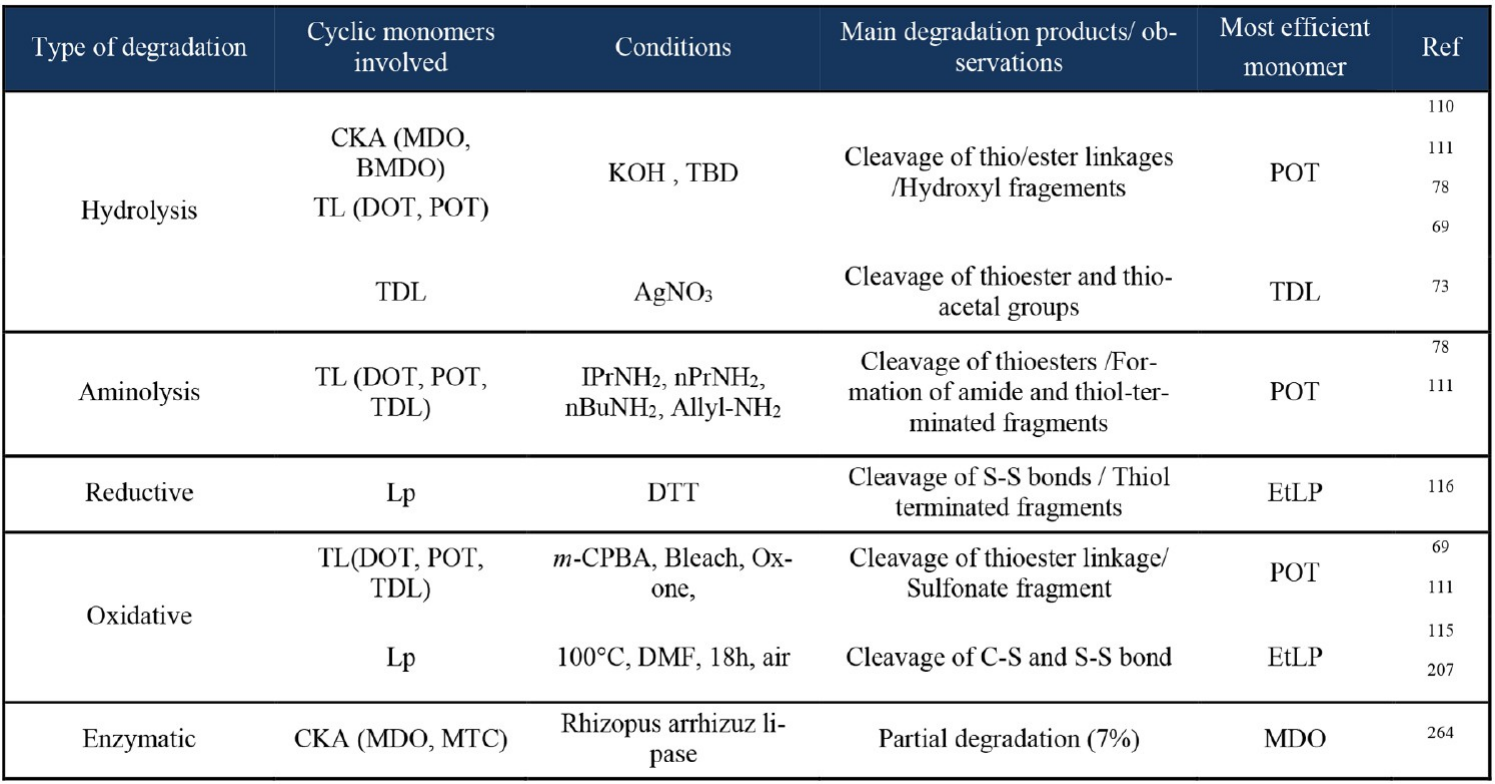
Summary Table of the Different Modes of Degradation for Styrene-Based Copolymers

#### Polyacrylates/Acrylamides

The copolymerization of CKAs and acrylates/acrylamides and analogues, such as vinyl azlactone, for example, is more favored than the one with styrene, and thus a more homogeneous incorporation of ester units is observed. Liu et al.^117^ copolymerized 2-methylene-1,3-dioxepane (MDO) with methyl acrylate (MA) via radical polymerization in solution, yielding copolymers containing 1.1 to 17.9 mol % MDO depending on the initial feed composition. The resulting number-average molar masses ranged between 1.4 × 10^5^ and 3.41 × 10^5^ g·mol^–1^. They further examined the hydrolytic stability of the MDO/MA copolymer films in phosphate buffer over 175 days at 37 °C. Whatever the amount of ester units into the copolymer, no significant changes in molar mass nor mass loss were detected, confirming high hydrolytic stability in aqueous nonenzymatic conditions for such hydrophobic copolymer.

The films were then immersed in PBS containing either worm enzymes or proteinase K for 10 to 25 days, and degradation was monitored through gravimetric mass-loss measurements combined with SEC analysis. While the PMA homopolymer did not undergo any degradation under these conditions, the P­(MDO-*co*-MA) copolymers exhibited a decrease in both *M*_n_ and *M*_w_, with faster degradation observed at higher MDO contents. In particular, P­(MDO_11_._6_-*co*-MA) showed a progressive decrease in molar mass throughout enzymatic treatment.^117^ These results demonstrate that a nondegradable PMA can become enzymatically degradable upon incorporation of MDO units within the main chain.

Poly­(butyl acrylate-*co*-BMDO) has been prepared by Matyjaszewski and co-workers.^120^ Hydrolytic degradation was conducted under acidic conditions (diluted H_2_SO_4_ in THF/butanol, 80 °C). All BMDO/nBA copolymers exhibited hydrolytic degradability, evidenced by a reduction in molecular weight during hydrolysis. Water-soluble polyacrylate based copolymers were also prepared. For example, Agarwal and co-workers^118^ copolymerized MDO with propargyl acrylate (PA) via radical polymerization in THF. The polymer was thus grafted with PEG-N3 to afford water-soluble copolymers. Hydrolytic degradation was conducted in aqueous NaOH (5 wt %) at room temperature for 24 h, resulting in a substantial decrease in molar mass, confirming cleavage of the ester linkages produced upon ring opening. Copolymers from a glucose-derived CKA (Glu-CKA) and either MA or *N*,*N*-dimethylacrylamide (DMA) was prepared using a 1:10 Glu-CKA:vinyl monomer feed ratio.^57^ The materials were subjected to base-promoted degradation using sodium methanolate (MeONa) in a CH_2_Cl_2_/MeOH mixture for 16 h. The *M*_n_ of the copolymer prepared with MA decreased from 74,700 g·mol^–1^ (*Đ* = 3.17) to 6,700 g·mol^–1^ (*Đ* = 2.80), while that synthesized with DMA decreased from 55,100 g·mol^–1^ (*Đ* = 2.73) to 11,300 g·mol^–1^ (*Đ* = 7.66), confirming efficient cleavage of inserted ester linkages.

Acrylamide-based copolymers incorporating CKA units have also been investigated as potentially degradable vinyl polymers, particularly using *N*-isopropylacrylamide (NIPAm) as a comonomer due to its good aqueous solubility and thermoresponsive behavior. Hiraguri and Tokiwa^268^ copolymerized NIPAm with MTC (up to 35/65 mol/mol), affording copolymers susceptible to enzymatic cleavage. When exposed to an aqueous medium at 37 °C containing *Rhizopus oryzae* lipase, a decrease in molar mass was observed exclusively for the copolymers, whereas PNIPAm homopolymer used as control remained stable under identical conditions. Ren and Agarwal^269^ subsequently prepared P­(NIPAm-*co*-BMDO) copolymers that underwent hydrolytic degradation in basic medium (KOH/H_2_O, 24 h, room temperature). More recently, several AAm-CKA copolymers were developed by the Nicolas group using three cyclic monomers (MPDL, MDO, and BMDO), leading to statistical or RAFT-based copolymers, fully water-soluble when AAm units were predominant.^239^ The CKA content could be finely adjusted to control both physicochemical properties and degradation kinetics. Degradation was investigated under various representative conditions including (i) enzymatic treatment in PBS (pH 7.4) containing immobilized *Candida antarctica* lipase, (ii) alkaline hydrolysis (KOH 5 wt %, 30 °C), and (iii) mild aqueous hydrolysis (PBS pH 7.4 or deionized water, 37 °C). Under physiologically relevant conditions (PBS, pH 7.4, 37 °C), these materials displayed rapid and tunable degradation, outperforming other vinyl-based polymers containing ester units but also reference polyesters such as PLA and PLGA evaluated under the same conditions (see Figure 52). Finally, acrylamide-based gels or networks were also prepared, containing either BMDO^270^ or MDO.^119^ The materials degraded in DMEM medium or in the presence of proteinase K. Figure 62 illustrates the effect of temperature on the enzymatic degradation of such a P­(NIPAM-*co*-MDO) network by proteinase K.^119^ The degradation at 22 °C occurred at a faster rate compared to that at 37 °C. The three-dimensional network of P­(NIPAM-*co*-MDO) hydrogels undergoes shrinkage at temperatures exceeding 33 °C, which complicates the action of proteinase K on the polymeric substrates.

**Figure 62:**
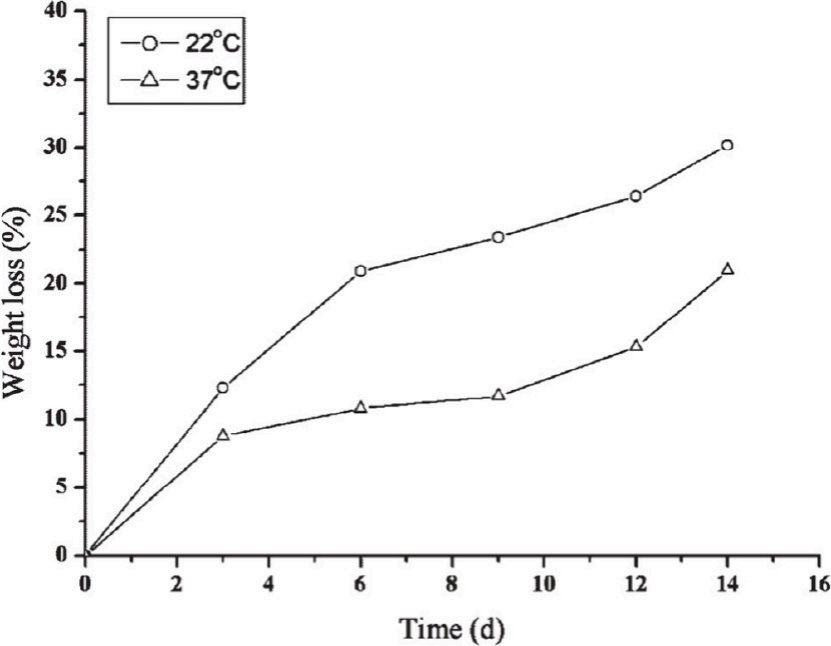
Temperature influence on the enzymatic degradation by proteinase K of P­(NIPAM-*co*-MDO) hydrogels. Reproduced from ref 119 with permission. Copyright 2003 Wiley-VCH.

Conversely, the hydrogels exhibit swelling and macroporosity at 22 °C, thereby enhancing the accessibility and catalytic activity of enzymes. In continuation of these studies on acrylate–CKA systems, Thoniyot et al.^237^ synthesized a degradable poly­(acrylic acid-*co*-MDO) copolymer by radical copolymerization of *tert*-butyl acrylate and 2-methylene-1,3-dioxepane (MDO), followed by deprotection of *tert*-butyl groups under acidic conditions (TFA/THF).

The incorporation degree of ester linkages along the main chain was 21%, and the material exhibited a number-average molar mass of 55,000 g·mol^–1^ (*Đ* = 4), which decreased to 12 000 g·mol^–1^ after deprotection. Chemical degradation was studied by basic hydrolysis (KOH) in water at room temperature for 24 h, leading to a pronounced decrease in molar mass down to approximately 350 g·mol^–1^, as confirmed by SEC and NMR analyses. Biodegradability tests performed on the resulting oligomers according to the OECD 301D protocol showed a mineralization of 27.5% after 28 days, while the poly­(acrylic acid) reference exhibited no measurable degradation under the same conditions (Figure 63).

**Figure 63:**
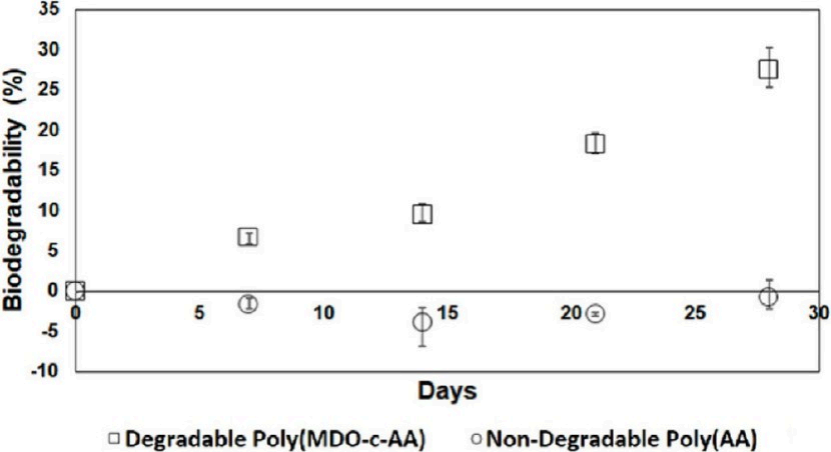
OECD 301 D Closed Bottle Test biodegradability (%) results of degradable P­(MDO-*co*-AA) and nondegradable poly­(AA) using secondary effluent from domestic wastewater treatment plant, as inoculum. Results shown are the average of the triplicates, Following the OECD Guideline for Ready Biodegradability, the test results were valid since reference compound achieves more than 60% biodegradability on Day 14. Reproduced from ref 237 with permission. Copyright 2022 Elsevier.

The results are promising; however, it is important to note that, based on the established standard, a compound is deemed biodegradable if its biodegradation rate attains 60% within 14 days. This result is the first to confirm the potential of incorporating weak bonds into polymers synthesized via radical polymerization to enhance biodegradability. This finding aligns with a recent study by Carter et al.,^271^ which confirmed the biodegradability of poly­(acrylic acid) oligomers up to a degree of polymerization of 17, noting a decrease in biodegradability with increasing chain length.

In the seminal reports from Roth et al.^36^ and Gutekunst et al.,^37^ thionolactone were shown to efficiently copolymerize with acrylate and acrylamide derivatives. Roth et al. reported efficient cleavage by aminolysis^36^ (5.8 M isopropylamine in dichloromethane overnight), whereas Gutekunst et al. proposed two distinct degradation conditions to evaluate the cleavage of thioester units.^37^ Treatment with sodium methoxide (NaOMe) of a poly­(*tert*-butyl acrylate-*co*-DOT) led to a molar mass decrease from 30 800 g·mol^–1^ (*Đ* = 1.15) to 1 750 g·mol^–1^ (*Đ* = 2.83), indicating efficient main-chain scission. A second strategy based on the addition of cysteine methyl ester yielded a comparable fragmentation, with a final *M*_n_ of 2 140 g·mol^–1^ (*Đ* = 3.24). The degradation products displayed terminal thiol groups, in agreement with the expected structure and consistent with the thioester deconstruction mechanism.^37^ In a similar manner than with styrene, Guillaneuf et al.^69^ investigated different mechanism for the degradation of a P­(IBA-*co*-POT). They found that TBD and KOH basic hydrolysis, aminolysis with isopropylamine and oxidation with *m*CPBA led to complete degradation. Roth and co-workers^126^ extended the use of DOT to water-soluble polymer via the copolymerization of various acrylamide and PEGA. In a comparison of aminolytic conditions, the authors found that thioester cleavage was favored by higher solvent polarity (dichloromethane < tetrahydrofuran ≪ methanol) and that ammonia served as a superior nucleophile compared to isopropylamine, enabling complete degradation at room temperature overnight with lower concentrations. Additionally, triethylammonium propanethiolate demonstrated even greater effectiveness than ammonia. An attempted degradation of a DMAm-DOT copolymer in 1 M aqueous HCl resulted in no degradation, whereas basic hydrolysis with 2 M aqueous NaOH at room temperature overnight resulted in complete cleavage of the backbone thioesters. Moreover, potassium persulfate (oxone) was identified as a rapid and selective agent for oxidative copolymer degradation, alongside the common nucleophiles.

The degradation behavior of DOT-PEGA copolymers was then evaluated in water, phosphate buffered saline (PBS, pH = 7.4), and in the presence of various degrading agents (Figure 64).^267^ P­(PEGA-DOT) underwent degradation with cysteine at 37 °C in PBS at pH 7.4 to replicate the physiological extracellular ionic strength and pH conditions.

**Figure 64:**
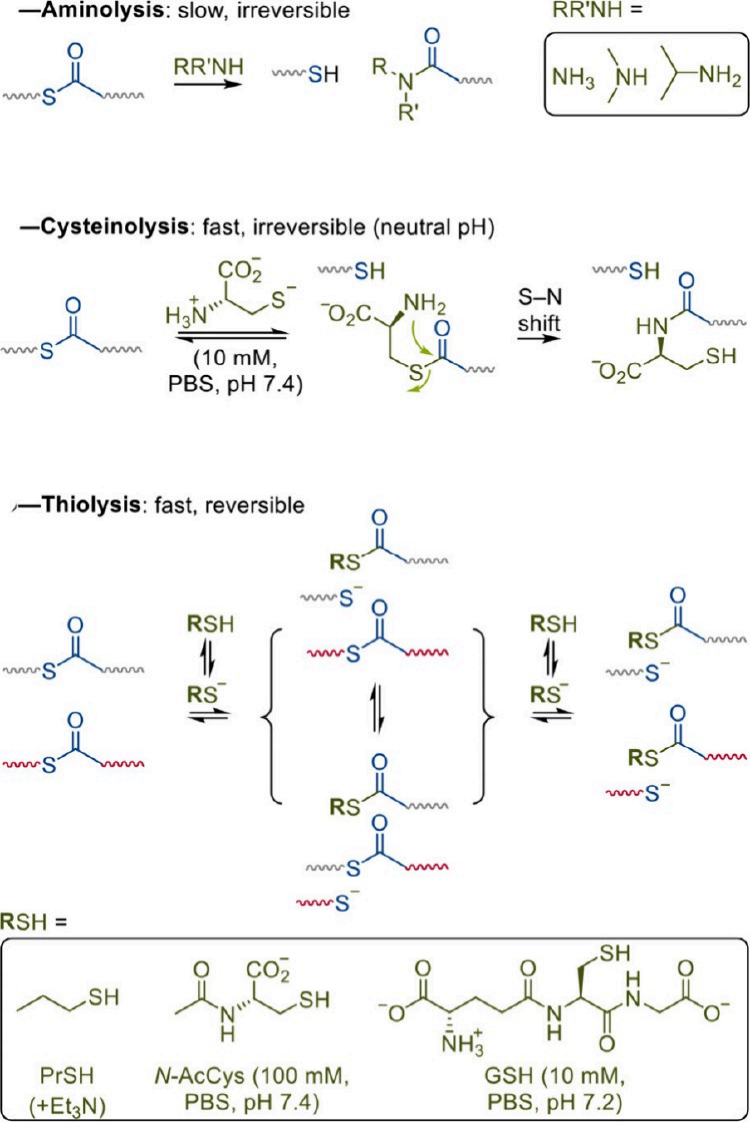
Various ecmhanisms of degradation for polyacrylate and polyacrylamide-based copolymers containing DOT units into the backbone. Reproduced from ref 267 with permission. Copyright 2022 American Chemical Society.

The degradation was assessed as complete after 1 day in both 100 mM and 10 mM cysteine solutions, corresponding to thiol/thioester ratios of 200 and 20, respectively. Treatment with *N*-acetylcysteine (100 mM) under identical conditions did not result in complete degradation of the copolymer. The observed difference in degradation efficiency between cysteine and its N-acetylated derivative is attributed to two factors. Cysteine possesses a relatively low p*K*_a_ (S–H) of 8.3, indicating that approximately 12% of molecules exist in the deprotonated reactive thiolate form at pH 7.4. *N*-Acetylcysteine, which has a p*K*_a_ (S–H) of 9.5, experiences approximately 0.8% deprotonation at pH 7.4. Second, a difference in degradation mechanism between the two cysteine derivatives was presumed. During cysteinolysis, S-acylated cysteine derivatives are known to undergo a S–N shift to form the amide-functional product. The degradation involving *N*-acetylcysteine does not include this additional step, making the degradation process (via thiol–thioester exchange, a potentially slow reaction) reversible. The expelled macromolecular thiol can substitute an S-acylated *N*-acetylcysteine residue, resulting in the formation of a new polymer–SC­(O)–polymer linkage.

The reversibility during thiolysis was particularly evident when glutathione (GSH), which also does not exhibit the irreversible S–N shift, was utilized. When P­(PEGA_218_-DOT_22_) was treated with 10 mM GSH in PBS at pH 7.2, simulating the intracellular pH and GSH concentration, a clear shift in SEC was observed after 10 min at 37 °C, but no additional changes were observed after 1 h or 2 days and the degradation was incomplete. A similar study on the aqueous degradation of water-soluble polyacrylamide containing thioester groups was carried out by Nicolas and co-workers^242^ that confirmed the previous results and that extend the degradation to commercial bleach.

First generation of SCM monomers was not able to efficiently copolymerize with acrylate/acrylamide monomers. Nevertheless, the use of second generation SCM monomers (SCM8–10) developed by Niu and co-workers, combined with a photo-RAFT process at room temperature allowed the good incorporation of such monomers into various acrylate/acrylamide monomers.^124^ Degradation was carried out in a MeOH/THF mixture using sodium methoxide (NaOMe), leading to efficient cleavage of ester linkages and formation of well-defined fragments. The degradation products, identified by ^1^H NMR, include methylated diester compounds derived from either from pure MCS or end-capped oligomers and the central diol.

The couple acrylates/acrylamides and lipoate derivatives is the more efficient to impart degradability to the copolymers. The traditional way to induce degradation is to use a reducing agent tris­(2-carboxyethyl)­phosphine (TCEP) in a solution of THF/water (4:1) at 60 °C,^34,49^ that will cleave the disulfide bond induced by diads of lipoate derivatives. Degradation using thiolates such as 2,2’-(ethylenedioxy)­diethanethiol (EDDET) in the presence of DBU (1,8-diazabicyclo[5.4.0]­undec-7-ene) as a catalyst, in THF is also reported.^218^ Boyer and Hawker^225^ showed that the use of DBU in dichloromethane allowed recovery of a part of the starting lipoate. Bates and colleagues^115^ presented a straightforward strategy to improve the recyclability of these systems by facilitating backbone degradation at reduced dithiolane loading levels, achieved through the cleavage of both S–S and S–C backbone units. Copolymers of *n*-butyl acrylate (nBA) containing small quantities of either α-lipoic acid (LA) or ethyl lipoate (ELp) dissolved in DMF demonstrated effective degradation upon heating at 100 °C in air. For instance, at just 5 mol % ELp, a high molecular weight P­(ELp-*co*-nBA) (*M*_n_ = 62 kg mol^–1^) underwent degradation to low molecular weight oligomers (*M*_n_ = 3.2 kg mol^–1^) through straightforward heating in DMF. In contrast, prolonged heating of P­(nBA) or homopolymer under identical conditions did not result in any alteration of molecular weight or cleavage of the C–C backbone.

The different experimental conditions for degradable acrylic-based copolymers are gathered in Table 6.

**Table 6:**
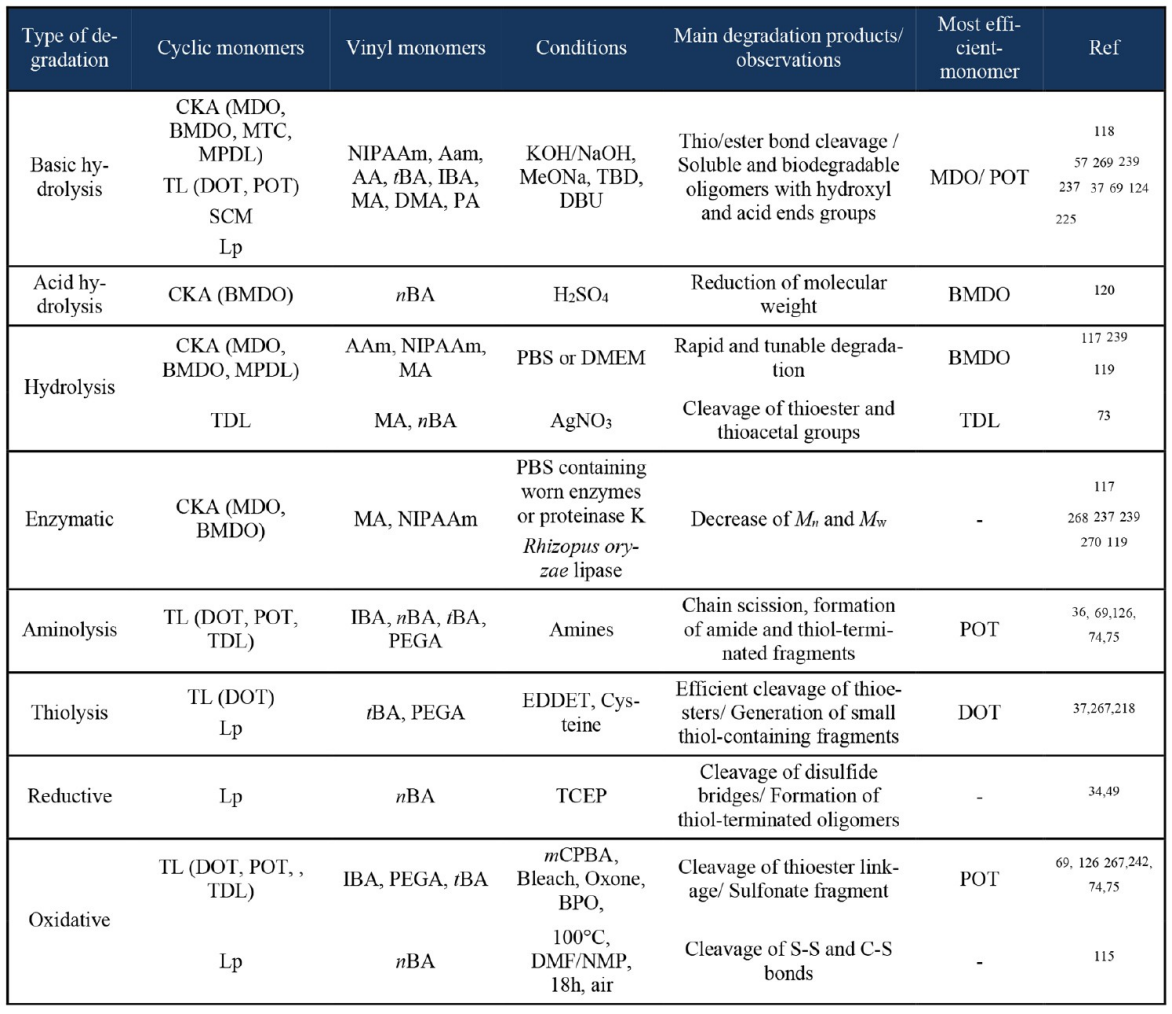
Summary Table of the Different Modes of Degradation for Acrylate/Acrylamide-Based Copolymers

#### Polymethacrylates/Methacrylamides

The methacrylate family is one of the most often utilized in copolymerization with CKA. Methyl methacrylate (MMA) was copolymerized with MDO, allowing the incorporation of up to 30% ester units in the polymer backbone.^137^ After 4 h of treatment with KOH (5 wt % in methanol), the SEC signal became undetectable, indicating complete degradation of the copolymer. The same copolymerization system was also used to prepare elastomeric block copolymer, and a similar degradation was observed.^146^ In PMMA-MPDL copolymers, Harrison, Nicolas et al.^52^ demonstrated a rapid and controllable alkaline degradation in a THF/MeOH mixture containing 5 wt % KOH at room temperature, monitored by SEC over 1 h. The copolymers exhibited *F*_MPDL_ = 0.03, 0.06, 0.14, and 0.29, with initial *M*_n_ values around 20–30 kg mol^–1^. The hydrolytic kinetics showed a direct relationship between the density of ester units and the rate of *M*_n_ decrease, with a 59% reduction observed for *F*_MPDL_ = 0.03, whereas *F*_MPDL_ = 0.29 resulted in more than 98% degradation within 5 min, reaching *M*_n_ approximately 0.5 kg mol^–1^. These results clearly highlight a quantitative dependence between the CKA incorporation level and the degradation efficiency, enabling fine-tuning of material stability through feed composition. Thin films of such PMMA-MPDL copolymers were prepared and their long-term hydrolytic degradation investigated in PBS at 37 °C.^272^ The copolymers exhibited slow degradation in PBS, with degradation kinetics that were slower than those of PCL. The erosion of these films indicates that the interplay between copolymer hydrophobicity and rigidity inhibits bulk erosion, resulting in a gradual surface erosion as evidenced by SEM and AFM analyses. MPDL was also copolymerized with syringyl methacrylate to prepare high *T*_g_ polymethacrylate materials.^273^ Basic hydrolysis for 3–72 h using KOH in THF/dioxane/MeOH was performed, and the residual was analyzed by SEC and MALDI-TOF.

Even if the chain end could not be determined, the two analyses confirmed a complete degradation of the ester groups coming from the MPDL opened units and the production of oligomers of poly­(syringyl methacrylate) with *n* = 6–7. Regarding PMMA–BMDO copolymers, Agarwal et al.^143^ investigated P­(HEMA-*co*-BMDO) films containing up to 43 mol % BMDO, processed as 1 mm-thick compression-molded films. Under alkaline conditions (5 wt % KOH, 37 °C), a mass loss higher than 50% after 17 h, followed by approximately 80% after 48 h, was observed, together with a decrease of *M*_n_ to around 2 kg mol^–1^, confirming degradation of the polymer backbone. In the same study, degradation of the films was evaluated in the presence of J774A macrophages, leading after 14 days to a 35–54% mass loss depending on cell density, along with a multimodal SEC evolution, indicating partial degradation under biologically relevant conditions.

Other methacrylate monomers were also copolymerized with CKA. Ionomeric P­(MDO-*co*-MMA-*co*-DMAEMA) quaternized with ethyl bromine containing approximately 40 mol % MDO exhibited environmentally driven degradation, in contrast to purely alkaline or enzymatic pathways. Agarwal and Ren^140^ reported that approximately 0.1 mm-thick films buried in industrial compost at 60 °C showed visible perforations and physical disintegration after 2 weeks, demonstrating a clear degradation of the material under composting conditions. Beyond hydrophobic systems, amphiphilic and/or water-soluble copolymers were developed through PEGMA incorporation or PEO-based macroinitiator, improving solubility, aqueous dispersibility, and potential biomedical applicability while retaining degradability. An illustration of this system was presented by Lutz et al.^139^ that introduced BMDO into P­(OEGMA) and proved its basic and moderate enzymatic (immobilized *Candida antarctica* lipases) aqueous degradation. Water-soluble P­(OEGMA-*co*-MPDL) copolymers exhibited considerable degradation during prolonged hydrolysis in PBS.^272^ The degradation kinetics were precisely adjusted by varying the MPDL content, resulting in degradation performances that lie between those of PLA and PCL (Figure 65), without leading to a dramatic drop of pH as for PLGA and PLA, thereby constituting a significant result. Nevertheless, the degradation of P­(OEGMA-*co*-MPDL) copolymers by enzymes was only moderate.

**Figure 65:**
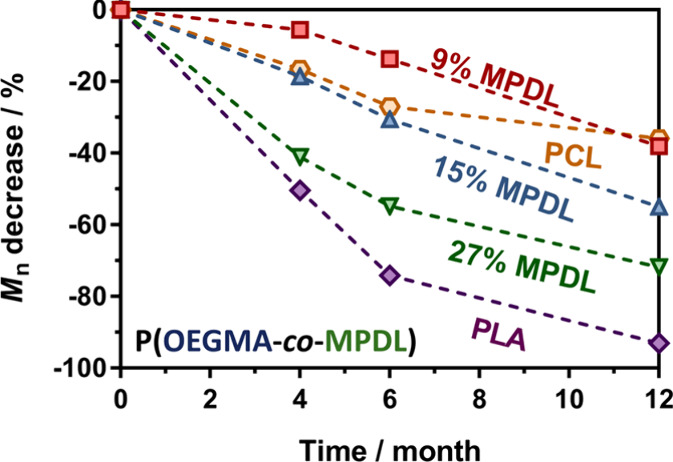
Evolution of the number-average molar mass, *M*_n_, with time of different P­(OEGA-*co*-MPDL) copolymers, PLA and PCL during the hydrolytic degradation in PBS (0.1 M, pH 7.4, 37 °C). Reproduced from ref 272 with permission. Copyright 2018 American Chemical Society.

This is likely attributed to the high hydrophobicity of MPDL units, the potential conformation of the copolymer chain due to hydrophobic interactions, and the steric repulsion of OEG side chains, which collectively hinder optimal enzymatic cleavage.^272^ Lastly, Agarwal and co-workers^142^ described P­(MDO-*co*-PEGMA-*co*-CMA) copolymers forming self-assembled micelles, showing alkaline degradation (1 wt % KOH, 24 h, room temperature) as well as lipase-mediated degradation (PBS 0.1 M, pH 7.4, 37 °C), demonstrating that micellar assemblies can be degraded under both alkaline and enzymatic conditions. This trend was further confirmed in 2013. Indeed copolymers prepared using a PEG-macroinitiator, and the comonomer pair MDO/DMAEMA and later quaternized with ethyl bromine (containing up to 51 mol % MDO), retained measurable alkaline degradability (5 wt % KOH, 24 h, room temperature) while significantly improving aqueous solubility and enabling biological use, demonstrating that hydrophilicity does not suppress CKA-induced degradability.^141^

As already stated, copolymerization of thionolactones and methacrylate derivatives are rather scarce. Johnson and co-workers^70^ developed a new thionolactone that reacted efficiently with MMA. Degradation was evaluated for such copolymers containing 2.5 to 10 mol % of thioester units. Following basic treatment with DBU (5 vol %) in propylamine at 50 °C for 24 h, a marked decrease in molar mass was observed, confirming the effective cleavage of thioester linkages. The resulting oligomers were end-functionalized with thiol groups, as expected for this selective deconstruction strategy. Later, Guillaneuf et al.^274^ prepared PMMA containing thioester units via the terpolymerization of methacrylate derivatives and DOT using *N*-phenyl maleimide as auxiliary comonomer. Due to the presence of *N*-phenyl maleimide, the copolymers were degraded using an ammonia solution in methanol that selectively cleaved the thioester bonds without impacting the imide group. They also observed that thermally triggered degradation could occur at 180 °C, that is driven by the presence of *N*-phenyl maleimide-DOT diads or triads.^274^

Concerning the first-generation SCM, the comonomers of choice are methacrylate derivatives (see below). Hawker and colleagues^41^ revisited such structures and combined the SCM unit with various degradable moieties embedded in the monomer structure (SCM 5–7, Figure 9). The copolymerization of such SCMs with vinyl comonomers such methyl methacrylate (MMA), 2-hydroxyethyl methacrylate (HEMA) and *N,N*-dimethylaminoethyl methacrylate (DMAEMA) allowed the synthesis of copolymers incorporating ester and/or thioester bonds, as well as disulfide or silyl ether bridges along the polymer backbone. These reactive functionalities provide access to multiple and selective degradation pathways, adaptable to diverse chemical or biological environments (Figure 66).

**Figure 66:**
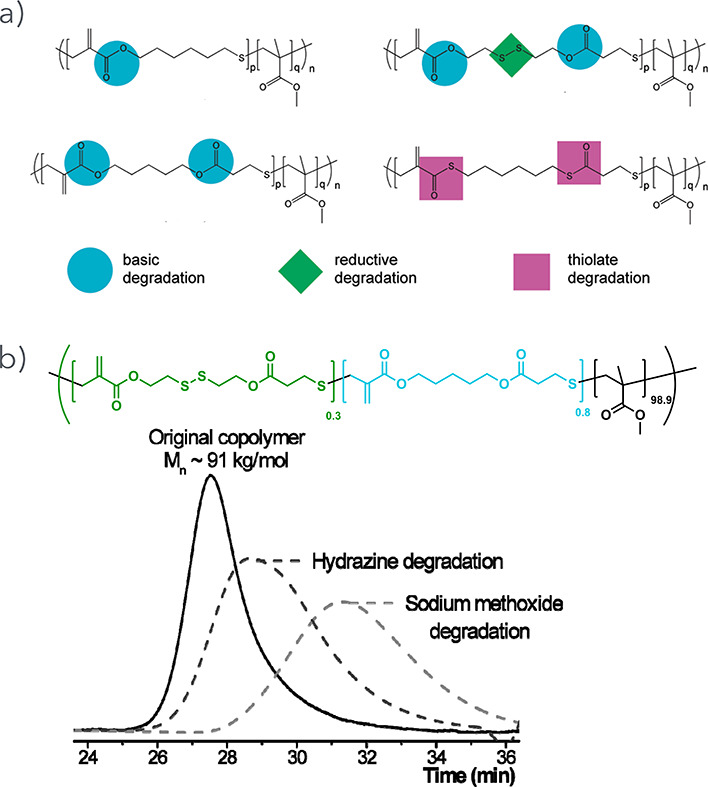
a) Various degradation conditions for polymethacrylate derivatives containing SCM5–7 monomers. b) SEC traces of the PMMA-based copolymer containing 0.3% of SCM7 and 0.8% of SCM5 and its products after stepwise degradation. Adapted from ref 41 with permission. Copyright 2009 American Chemical Society.

Disulfide bonds present in these copolymers were efficiently reduced, notably using hydrazine in organic solvents (THF or methanol), or by sodium methoxide (NaOMe), with full reaction occurring within 30 min at room temperature. Thioester linkages could also be selectively cleaved by NaOMe or sodium thiomethoxide (NaSMe). For DMAEMA-based copolymers, rapid degradation was observed under basic conditions following NaOMe treatment (30 wt % in THF/methanol), with complete degradation within 30 min at ambient temperature. In parallel, for HEMA-based copolymers, transesterification under acidic conditions (MeOH/H_2_SO_4_ mixture) also enabled efficient breakdown, with the decrease in molar mass correlating to the amount of cyclic monomer incorporated. Finally, an enzymatic route was explored for DMAEMA-containing copolymers: partial degradation was observed after 4 h of incubation at 37 °C in the presence of pig liver esterase (PLE), highlighting the potential of these materials for biodegradable applications.^41^

On the similar first-generation SCM structure, Roth and co-workers^275^ tuned the rate of degradation by preparing six lactones exhibiting diverse functions at the 2-position of the ring (H, ethyl, decyl, furyl, and phenyl groups (Figure 67). Since these groups are not located close to the SCM functionality, the reactivities of these monomers are rather similar, but the ester group is more or less protected by different hydrophobic groups. Copolymerization with biocompatible methacrylate OEGMA along with two methacrylamides (*N*-isopropylmethacrylamide (NIPMAm) and *N*-(2hydroxypropyl)­methacrylamide (HPMAm)) were performed and the rate of accelerated degradation determined (8 mmol NaOH in water–methanol). Differences were observed in the degradation rates of the three copolymers, with the Et-SCM species exhibiting the slowest degradation and the Ph-SCM species the fastest. While other research has indicated that the degradation rate is primarily influenced by hydrophobicity, as demonstrated by Pesenti et al.^168^ with CKAs, these findings suggest that these rates correspond to the order of p*K*_a_ values of primary (H-SCM), secondary (Et-SCM), and benzylic (Ph-SCM) alcohols. This indicates that the stability of the alcoholate leaving group primarily may dictate the rate of backbone ester hydrolysis.

**Figure 67:**
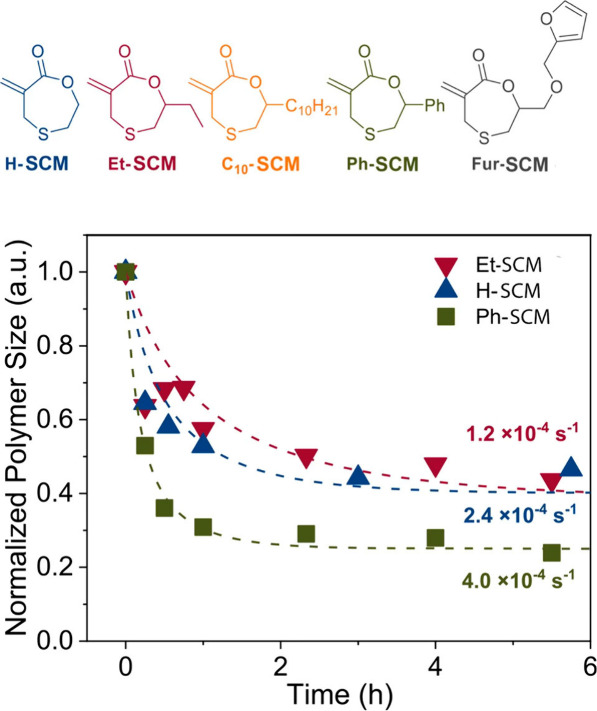
Change of SEC-measured molar mass (normalized to intact species) versus time during the hydrolysis (in 8 mM NaOH in water–methanol) of three p­(R-SCM-*co*-HPMAm) copolymers. Values are the fitted rates of degradation for each copolymer. Adapted from ref 275 with permission. Copyright 2025 American Chemical Society.

Lastly, second-generation SCM monomers developed by Niu et al.^150^ were also compatible with methacrylate derivatives and some degradation with strong bases such as NaOMe led to a large reduction of the *M*_n_, confirming the good incorporation of the SCM units in the polymer backbone.

The different experimental conditions for degradable methacrylic-based copolymers are gathered in Table 7.

**Table 7:**
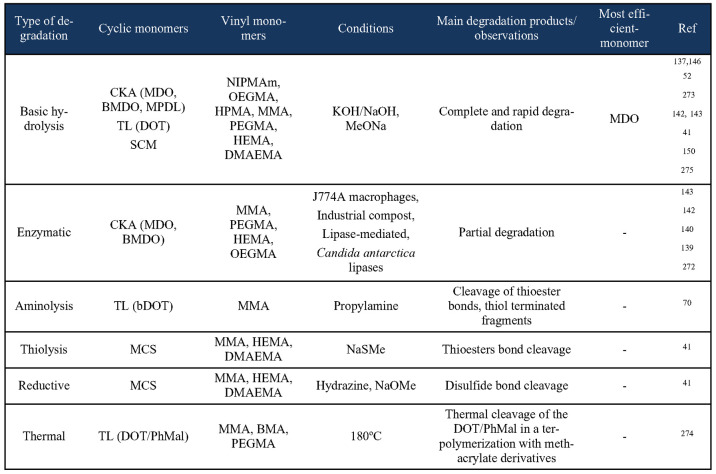
Summary Table of the Different Modes of Degradation for Methacrylate-Based Copolymers

#### Nonstabilized Monomers

Bailey and colleagues^26^ were the first to document the copolymerization of MDO and VAc, a topic subsequently explored further by the groups of Agarwal,^152^ Albertsson,^153^ and Dove^159^ due to a rather similar reactivity of the two monomers. The Carter group^188^ also reported on the emulsion polymerization of MDO and VAc, leading to the synthesis of a backbone degradable VAc latex, achieving a notable 90% incorporation of MDO in the VAc backbone.

It was observed that films coated with poly­(VAc-*co*-MDO) latex particles, designed for biodegradable food container coatings, experienced a 50% weight loss when subjected to pH 10 water over a period of 100 days.^188^

The hydrolytic degradation observed suggests that these polymers and microparticles possess the potential for rapid biodegradation in water, which is essential for environmentally friendly products such as controlled release particles utilized in personal and consumer care. Thoniyot and co-workers^200^ focus on random copolymerization and investigated first the accelerated hydrolytic degradability through alkali hydrolysis, leading to the formation of oligomeric poly­(vinyl alcohol) degradation products, end-capped by OH and COOH groups. The PVAc is usually a precursor to the hydrosoluble poly­(vinyl alcohol) that could be a good alternative to other hydrophilic part in surfactant, Nevertheless, it was not possible to prepare PVA containing ester units via deacetylation of the PVA moiety even under mild conditions. To tackle this challenge, Thoniyot et al.^200^ employed an alternative method by synthesizing poly­(VA-*co*-MDO) through the selective cleavage of the chloroacetyl ClAc group from its precursor, P­(VClAc-*co*-MDO).

In a second study, they also prepared P­(VA-*b*-MDO) by RAFT copolymerization.^199^ In the OECD 301 D Closed Bottle Test conducted over a 28-day period, P­(VA-*co*-MDO) containing 12% MDO units demonstrated a biodegradability of up to 89%. In contrast, the block copolymer comprising 35 mol % poly­(VA) and 65 mol % P­(MDO) blocks, as well as the degradable poly­(VA-*b*-MDO) with 64 mol % P­(VA) and 36 mol % P­(MDO) blocks, achieved biodegradability rates of only 78% and 33%, respectively (Figure 68).

**Figure 68:**
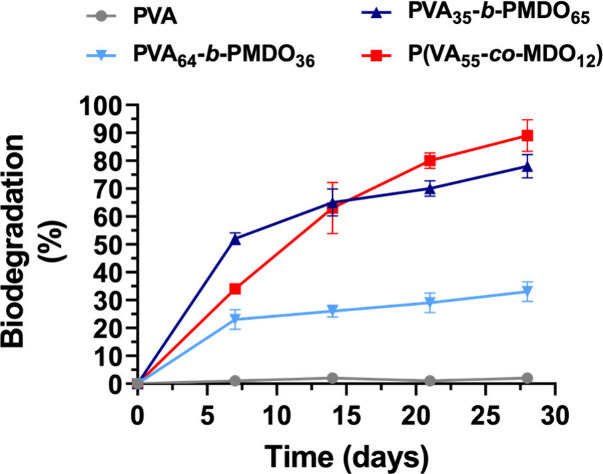
OECD 301 D Closed Bottle Test biodegradability (%) results of solid sample of nondegradable PVA (*M*_n_ = 5,100 g·mol^–1^), degradable P­(VA-*co*-MDO) (88% VA units, *M*_n_ = 1,700 g·mol^–1^) and nanoparticles of degradable P­(VA-*b*-MDO) (35% VA units, *M*_n_ = 7,300 g·mol^–1^) and degradable P­(VA-*b*-MDO) (64% VA units, *M*_n_ = 12,000 g·mol^–1^) using secondary effluent from domestic wastewater treatment plant, as inoculum. The results shown are the average of the triplicates. Adapted from refs 199 and 200 with permission. Copyright 2023 Elsevier.

The results indicate that the inclusion of degradable ester linkages improves the biodegradability of P­(VA-*co*-MDO), that may be attributed to the periodic distribution of these degradable ester units within the polymer.

Copolymers of vinyl acetate (VAc) were also synthesized with the more hydrophilic MTC. The P­(MTC-*co*-VAc) copolymer, with a molar mass of 13 000 g·mol^–1^ (*Đ* = 3.1), underwent enzymatic hydrolysis using *Rhizopus arrhizus* lipase, resulting in a partial solubilization of 15%.^264^

*N*-Vinylpyrrolidone (NVP)-based polymers are extensively utilized in the pharmaceutical industry. Furthermore, the inhibition of adhesion and the capacity for dispersion render NVP-based polymers important additives in high-performance home-care formulations. Various P­(CKA-*co*-NVP) were prepared by Coughlin et al.^163,164^ as substrates for parallel degradation studies under different degradation conditions. They employed a chemical base, various enzymes, and activated sludge sourced from a wastewater treatment facility. Samples of P­(MTC-*co*-NVP) or P­(BMDO-*co*-NVP) containing 75 mol % NVP were subjected to alkaline degradation using KOH/MeOH at pH 11.

The methanolysis products were analyzed via ESI-MS and DOSY NMR. The analysis identified oligomers containing NVP repeat units varying from 1 to 7 whatever the CKA structure. The degradability of CKA copolymers was assessed using an enzymatic assay, incorporating various enzymes and a pH indicator (bromothymol blue) for monitoring the release of carboxylic acid as a product of hydrolysis.^163^ A compostable polyesterurethane, certified in accordance with EIN 13432, was evaluated under identical conditions. The CKA homopolymer poly­(MTC) is subject to rapid hydrolysis by the various enzymes examined (Figure 69). The copolymers of CKA and NVP exhibited reduced enzymatic hydrolysis kinetics, probably attributable to the decreased density of backbone esters. The copolymers containing BMDO exhibited a more rapid enzymatic degradation compared to MTC-NVP copolymers with equivalent CKA incorporation levels and comparable molecular weights. This suggests that adjusting the backbone amphiphilicity through the inclusion of hydrophobic moieties, such as aromatic rings, enhances enzymatic hydrolysis. The kinetics of degradation is influenced by the classes of enzymes involved with cutinases being one of the more efficient enzymes to have an efficient degradation.

**Figure 69:**
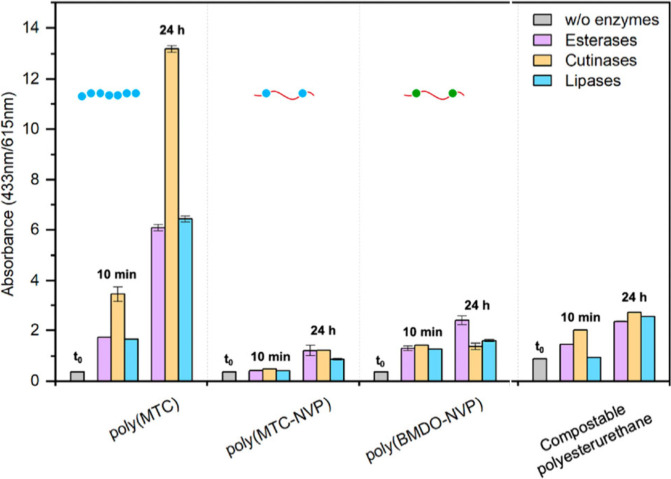
Enzymatic hydrolysis test results for CKA-NVP polymers. Adapted from ref 163 with permission. Copyright 2024 Elsevier.

The study examined the biodegradation of polymers using activated sludge from a wastewater treatment facility, following to OECD guidelines. The OECD 301F test assesses the readiness of a polymer to biodegrade under aerobic conditions at a low concentration. In contrast, the OECD 302B test examines the inherent biodegradability of the polymer at a higher concentration, indicating its potential for biodegradation under optimal conditions rather than immediate environmental compatibility. The homopolymer poly­(MTC), despite its lack of water solubility, demonstrated significant biodegradability, achieving over 60% degradation within 28 days in accordance with OECD 301F guidelines. In contrast, the copolymer poly­(MTC-*co*-NVP) exhibited minimal biodegradation (less than 10% over 28 days) under the same protocol.

The biodegradation test of the MTC-*co*-NVP copolymer was then conducted in accordance with a modified OECD 302B protocol that was less stringent.^163^ In the test bottle, 574 mg DOC/L of MTC-*co*-NVP copolymer and 2 g/L of activated sludge were introduced, with periodic openings to facilitate aeration. Biodegradation in this instance achieved 27% after 28 days (Figure 70a). The degree of biodegradation, while limited, was not negligible. Upon completion of the test, the residuals were analyzed (Figure 70). A degradation pathway was proposed based on the analysis of end-groups. The clear identification of species with different end-groups linked to MTC strongly suggests that the MTC segments of the copolymer likely played a role in the biodegradation process. No signals indicative of enzymatic or microbial activity on NVP repeat units were observed, including amine, carboxy-, or hydroxy-pendant groups. The findings indicate that the limited biodegradability observed for the MTC-*co*-NVP copolymer under modified OECD 302B conditions is primarily attributable to the mineralization of MTC fragments.

**Figure 70:**
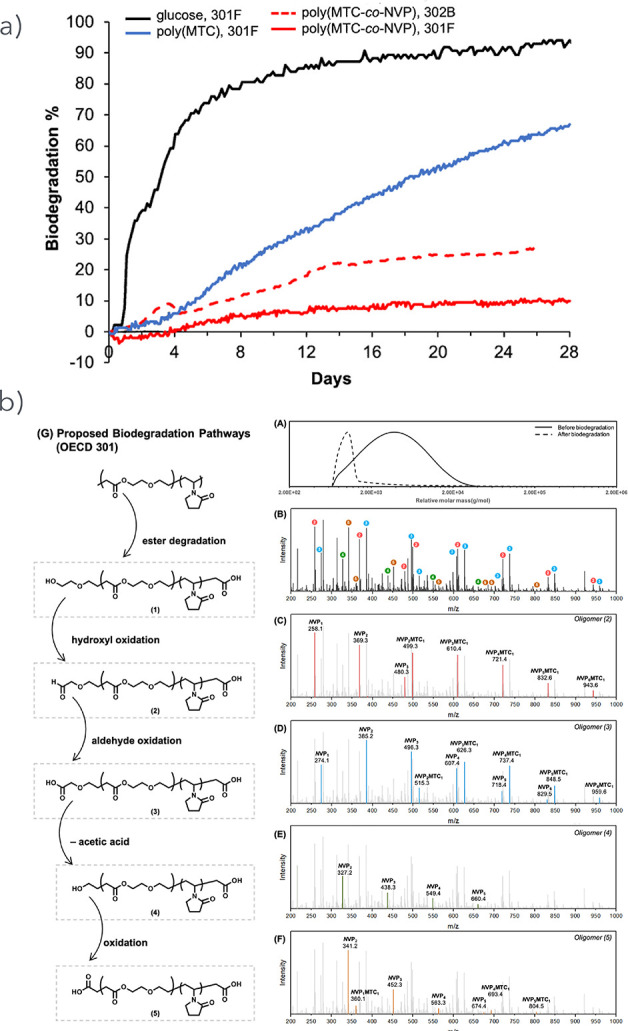
a) Biodegradation curves of CKA-based polymers following OECD 301F (solid lines) and modified OECD 302B (dashed line) protocols. b) SEC analysis of poly­(MTC-*co*-NVP) before and after modified OECD 302B biodegradation test. The assigned mass peak sets for species comprising one MTC unit from the spectrum (B) are listed in spectra (C–F), corresponding to oligomer structures (2)–(5). (G) Proposed biodegradation pathways, based on mass spectrometry analysis. Adapted from ref 163 with permission. Copyright 2024 Elsevier.

In contrast, the NVP oligomeric segments appear to have not been internalized by the cells and, consequently, were not metabolized. The degradation products were also subjected to the OECD 301F test, showing an oxygen consumption limited to 24%, confirming the absence of biodegradability in NVP oligomeric segments with a degree of polymerization less than 6.^163^

Another important copolymerization system is the CKA–vinyl ether comonomer pair, developed by Guillaneuf and Nicolas.^157,170^ P­(MDO) bearing PEG side chains were prepared via the copolymerization of various vinyl ethers followed or not by postmodification reactions. The copolymers underwent successful degradation under accelerated (THF with 1% NaOH in methanol), hydrolytic (PBS 1×, pH = 7.4, 37 °C), or enzymatic conditions (*Candida antarctica* at 37 °C). Hydrophobic copolymers exhibited degradation kinetics in PBS comparable to that of PCL, with complete degradation (−95% in *M*_n_ decrease) observed in the presence of enzymes (lipases). To tune the degradation profile, Nicolas et al.^168^ prepared a library of copolymers based on vinyl ethers, replacing MDO or BMDO by MTC a more hydrophilic comonomer. The insertion of MTC units was demonstrated to accelerate the hydrolytic degradation of the corresponding hydrophobic copolymers under accelerated (0.05 wt % NaOH in MeOH:THF 1:1 v/v) and physiologic conditions (PBS, pH 7.4, 37 °C), in comparison to those derived from traditional CKAs such as MDO and BMDO (Figure 71). This indicates that MTC units can effectively enhance water uptake and solvation of the ester groups, even in the presence of highly hydrophobic VE units. When a hydrophilic vinyl ether is used, no difference was observed, demonstrating that MTC did not enhance the cleavage of ester groups relative to MDO for these amphiphilic copolymers. Hydrolytic degradation can thus be accelerated through the use of MTC as a hydrophilic CKA or by employing a hydrophilic vinyl monomer.

**Figure 71:**
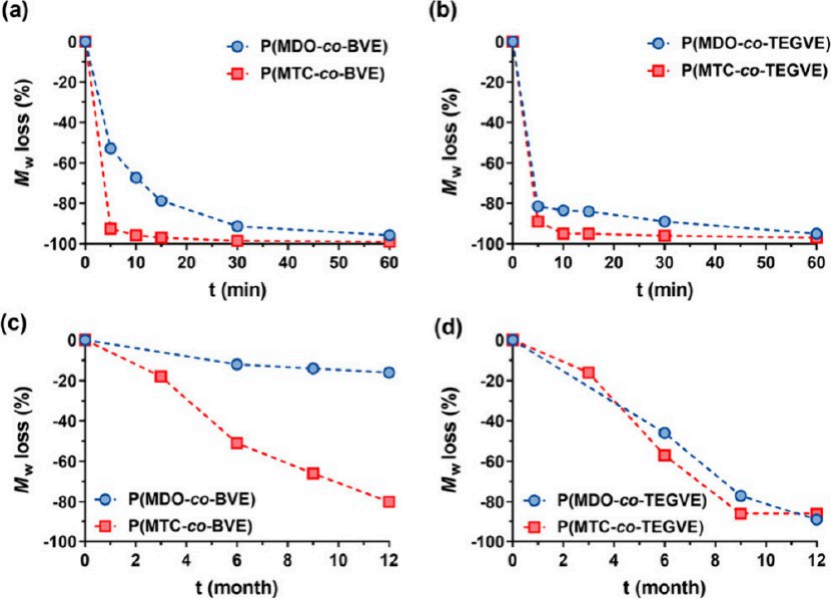
Evolution of the weight-average molar mass (*M*_w_) during the hydrolytic degradation under accelerated conditions (0.05 wt % NaOH MeOH:THF 1:1 v/v) (a, b) or under physiological conditions (PBS, pH 7.4, 37 °C) (c, d) of P­(MDO-*co*-BVE) and P­(MTC-*co*-BVE) copolymers, and P­(MDO-*co*-TEGVE) and P­(MTC-*co*-TEGVE) copolymers. Adapted from ref 168 with permission. Copyright 2023 American Chemical Society.

Lastly for CKA, Gapud and Bailey^252^ synthesized in 1985 some copolymers of ethylene and MDO, with MDO contents varying from 2 to 10 mol %. Initial biodegradation assessments indicate that these copolymers exhibit biodegradability in a soil model containing microbiota.

As discussed below, the groups of Destarac^72,171^ and Guillaneuf^71^ proposed independently unsubstituted thionolactones such as thionocaprolactone to impart degradability into polyvinyl ester derivatives. Both authors reported the degradation of the polymer by aminolysis using isopropylamine. When polymerized in bulk, Destarac and co-workers observed the introduction of the thionolactone into its ring-opening thioester group or into a thioacetal moiety occurring from 1,2 addition, the amount depending on the thionolactone ring size.^171^ Due to the occurrence of two different thionolactone insertion mechanisms, Destarac et al.^276^ proposed to use the orthogonal reactivities of the two functions to degrade polyvinyl esters via a stepwise reaction using aminolysis for thioester moieties and peroxides for thioacetal moieties. The use of bleach is also proposed as a universal agent for the polymer degradation (Figure 72).

**Figure 72:**
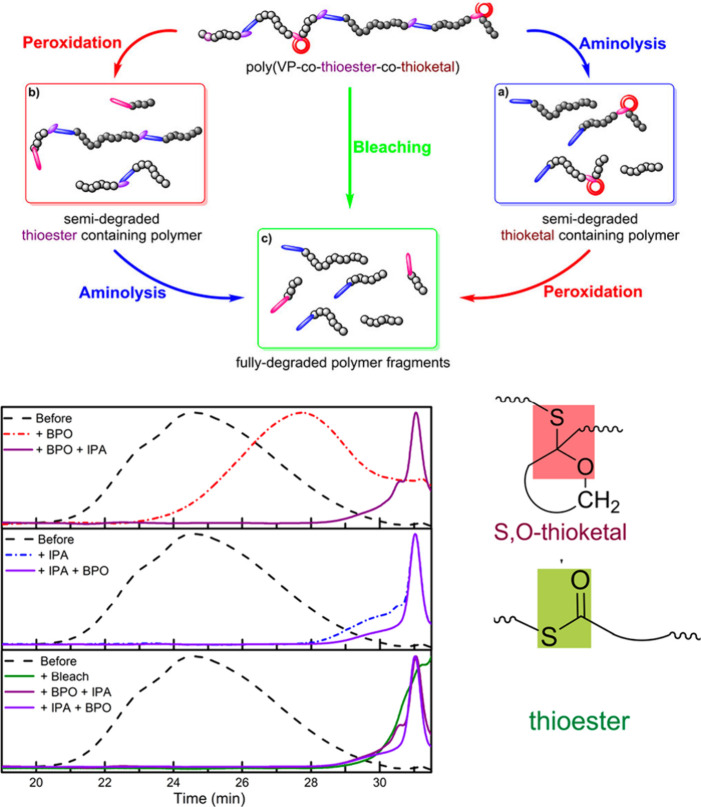
Schematic representation of the two-step (peroxidation/aminolysis) and one-step (bleach) degradation processes for P (vinyl pivalate-*co*-thionocaprolactone). Experimental SEC chromatograms before and after degradation: P­(TCL_0.68_-*co*-VP_0.32_) with benzyl peroxide (BPO) and subsequent addition of *N*-isopropylamine (IPA); IPA and subsequent addition of BPO; and bleach in comparison to the two-step degradation. Adapted from ref 276 with permission. Copyright 2023 American Chemical Society.

The different experimental conditions for degradable non stabilized monomers-based copolymers are gathered in Table 8.

**Table 8:**
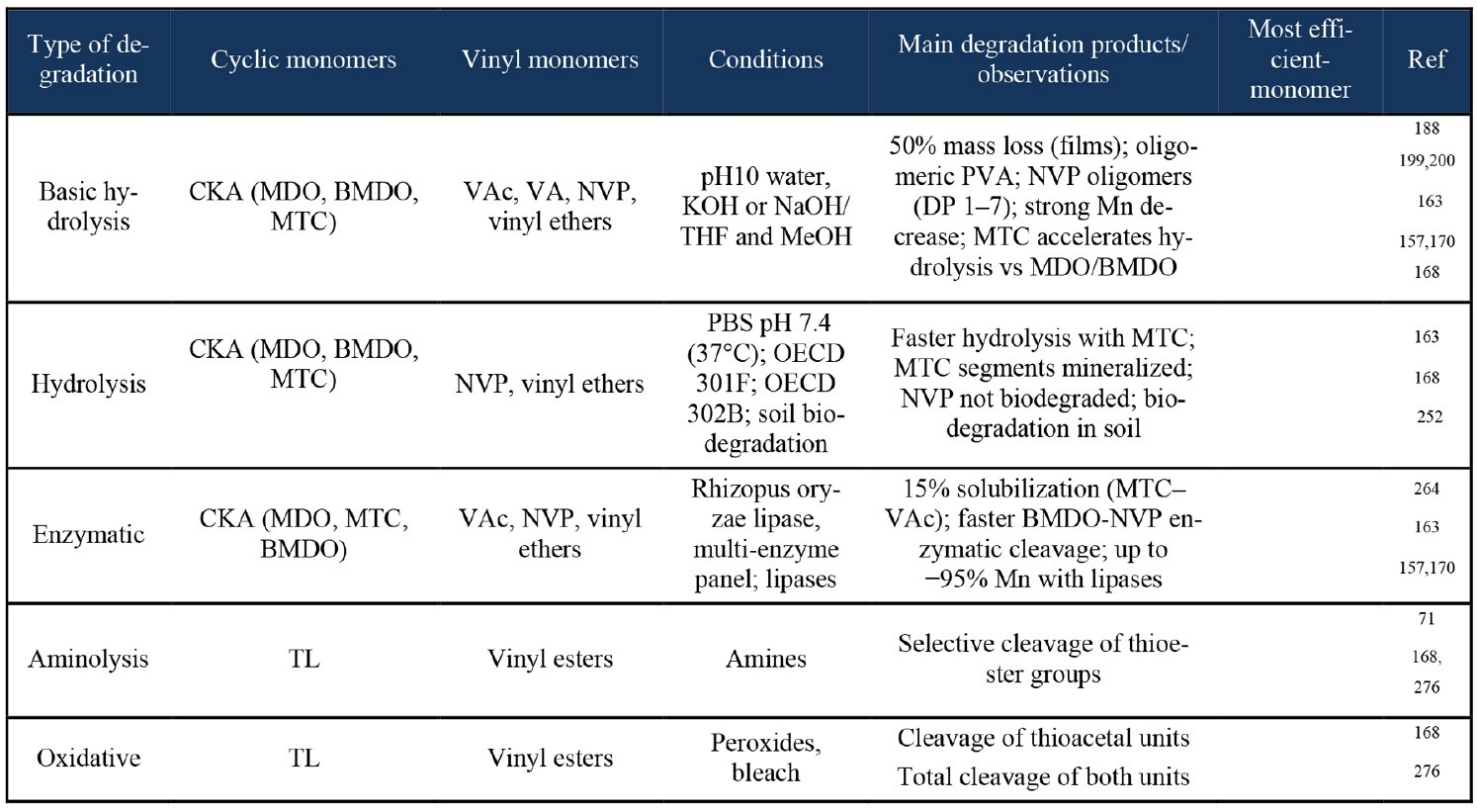
Summary Table of the Different Modes of Degradation for Non-Stabilized Monomers-Based Copolymers

## Recycling

The establishment of a circular material economy is a rational and optimal strategy to tackle the end-of-life issue of waste plastics, mitigate environmental damage, and decrease our dependency on limited fossil resources.

Chemical recycling, also known as tertiary recycling, is a flexible and effective approach to tackling the global plastic issue.^277^ Since the introduction of weak bonds into the polymer backbone led after degradation to telechelic oligomers, it could be worthwhile to use their end-chain functionality to perform recycling.^17^

The first example was reported by Johnson et al.^78^ that established a general chemical recycling strategy for polystyrene through the incorporation of thioester functionality via the copolymerization of styrene and DOT, followed by controlled deconstruction into α,ω-difunctional oligostyrenes using nucleophiles such as cysteamine·HCl/DBU in DMF at room temperature for 22 h. The resulting dithiol-terminated fragments (4.5–9.1 kDa) could then be repolymerized by mild oxidation (I_2_/pyridine, CH_2_Cl_2_, 1 h, RT), yielding recycled polystyrene with molar masses and dispersities comparable to those of the original polymer (example: rPS (11), *M*_n_ = 12.3 kDa, yield 89%) (Figure 73). Johnson et al.^78^ detailed also theoretically that the repolymerized oligomers should have a similar *M*_n_ and dispersity than the pristine copolymers and thus there is a “molecular weight memory effect” when repolymerizing such degraded oligomers.

**Figure 73:**
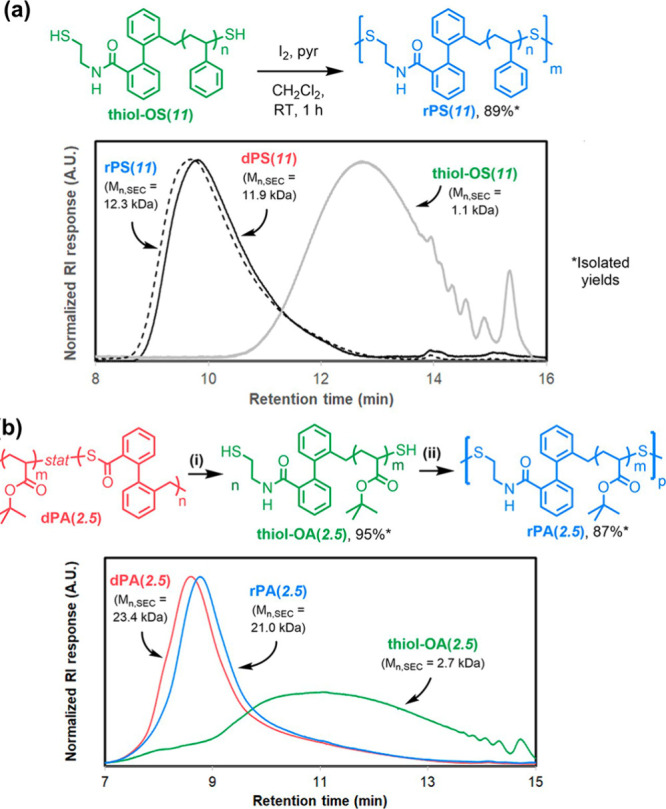
a) Synthetic scheme illustrating the deconstruction and recycling of high-molar-mass PS. SEC traces for the deconstruction/recycling cycles of P­(S-*co*-DOT). b) Application of the deconstruction/reconstruction strategy to an acrylic copolymer; SEC traces for the deconstruction/recycling cycles of P­(*n*BA-*co*-DOT). Reproduced from ref 78 with permission. Copyright 2022 American Chemical Society.

This process relies on the reversibility of the thiol/disulfide couple: disulfide bonds can be reduced (e.g., with LiAlH_4_) and subsequently reformed by oxidation, allowing multiple recycling cycles. Moreover, this approach was also extended to acrylic copolymers, for which closed-loop chemical recycling was similarly demonstrated, highlighting the generality and versatility of the method for deconstructible vinyl materials.

Pursuing this design for recyclable polymers, recent research has extended the concept of chemical recycling to lipoate-acrylate-type copolymers, particularly those used in pressure-sensitive adhesives (PSAs). Bates et al.^130^ designed degradable poly­(acrylates) by copolymerizing *n*-butyl acrylate with derivatives of α-lipoic acid and ethyl lipoate, enabling the introduction of reversible disulfide linkages along the polymer backbone. These materials, exhibiting high molar masses (*M*_n_ ≈ 143–198 kg·mol^–1^), retained mechanical and viscoelastic properties comparable to those of commercial poly­(acrylates). Exposure to mild reducing agents such as tris­(2-carboxyethyl)­phosphine (TCEP) or dithiothreitol (DTT) induced a drastic decrease in molar mass (*M*_n_ ≈ 175 → 13 kg·mol^–1^) through disulfide bond cleavage. The resulting thiol-terminated oligomers could then be reoxidized (I_2_/pyridine) to regenerate a high-molar-mass polymer (*M*_n_ ≈ 142 kg·mol^–1^), demonstrating closed-loop recycling without significant loss of performance (Figure 74).

**Figure 74:**
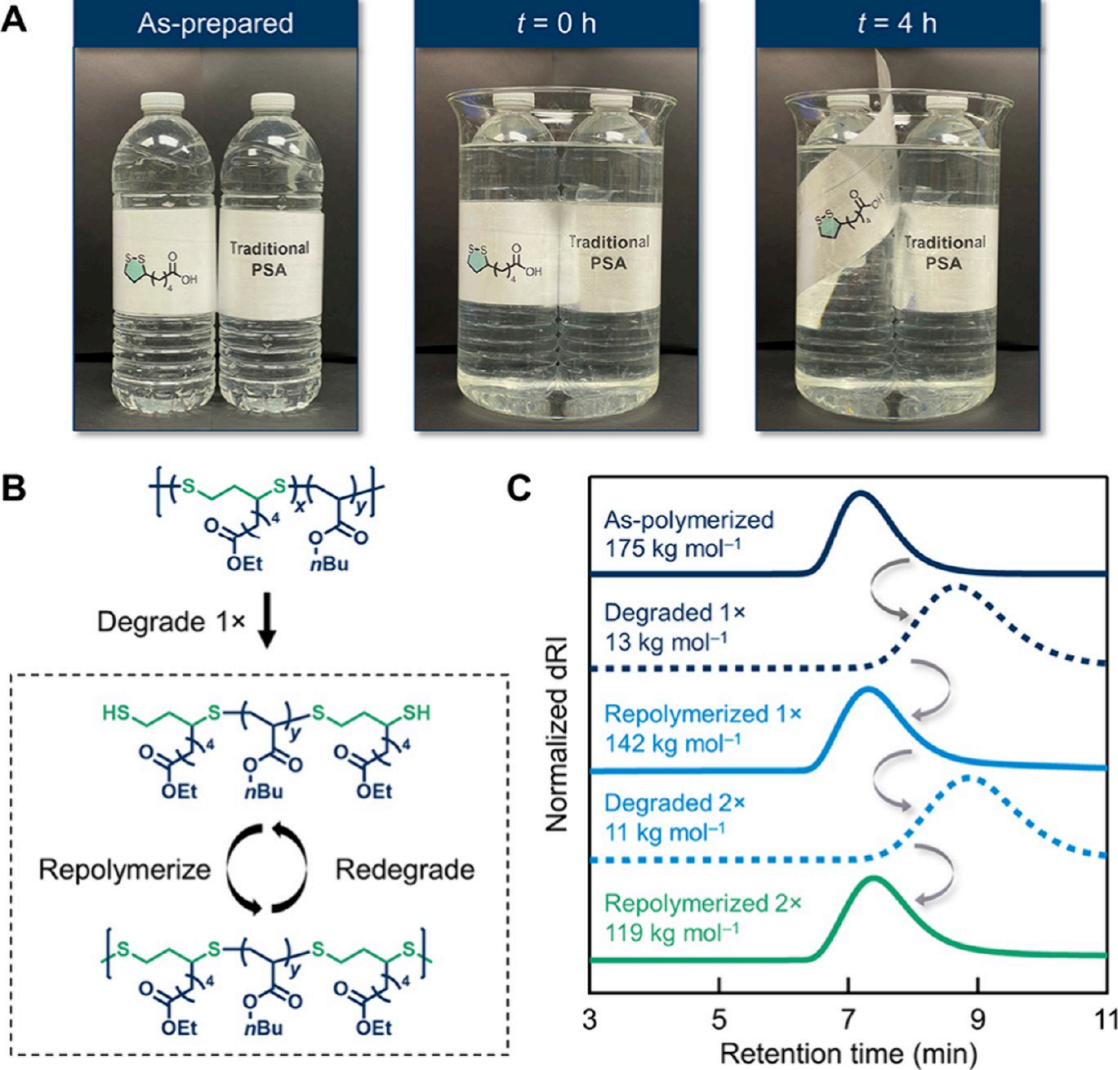
A) Removal of label adhesive (αLA-ELp-nBA and nBA-AA) attached to recyclable plastic bottles. B) Model adhesive (ELp-nBA) with functional chain-ends produced after degradation can undergo repeated oxidative repolymerization and reductive degradation for closed-loop recycling, as evidenced by c) size-exclusion chromatography analysis. Reproduced from ref 130 with permission. Copyright 2023 American Chemical Society.

In continuation of these developments, Hawker and Bates et al.^278^ introduced a simple and modular strategy that bridges the approaches previously developed for acrylate and styrenic systems. Their method relies on the copolymerization of vinyl monomers such as *n*-butyl acrylate, styrene, or siloxanes with ethyl lipoate (ELp), enabling the incorporation of disulfide linkages along the main chain. Upon reduction of these disulfide bridges by tris­(2-carboxyethyl)­phosphine (TCEP), the resulting copolymers are α,ω-dithiol telechelic blocks with number-average molar masses tunable between 2 and 32 kg·mol^–1^ depending on the initial composition. These blocks can subsequently be reconnected by mild oxidation (I_2_/pyridine) to form random multiblock copolymers of high molar mass (68–95 kg·mol^–1^), exhibiting a single *T*_g_ and a homogeneous morphology, indicating complete chemical compatibility between the styrenic and acrylate segments (Figure 75).

**Figure 75:**
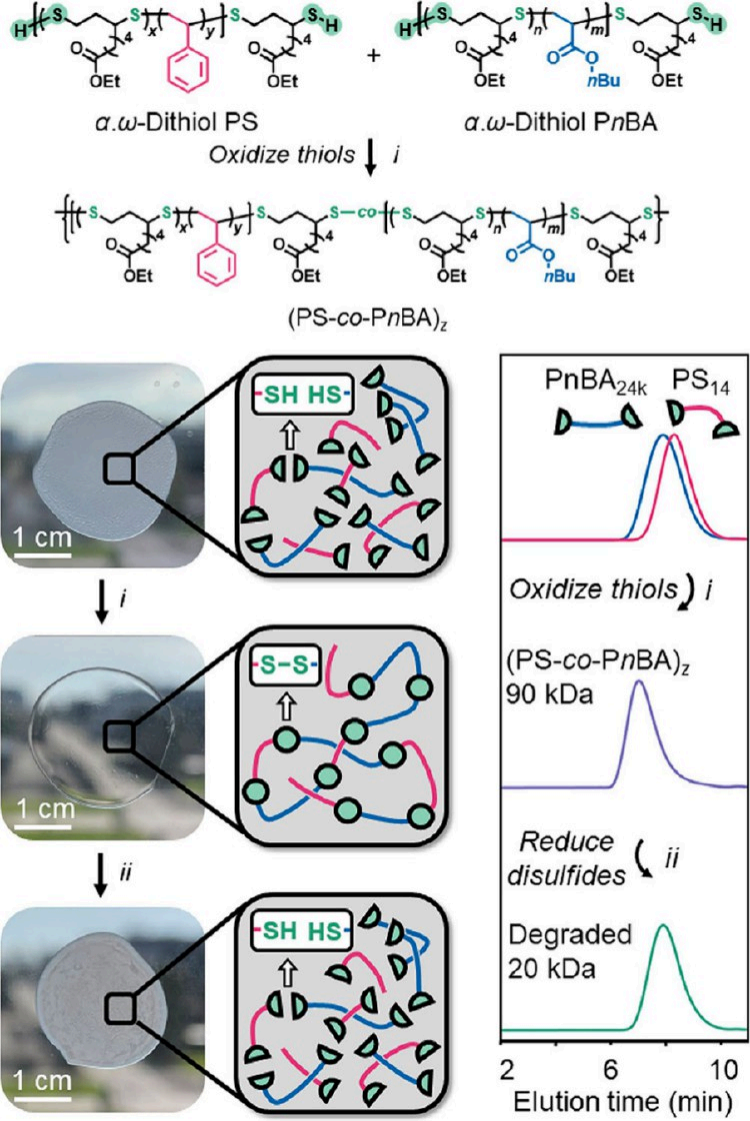
Closed-loop recycling of PS-PnBA multiblock copolymers containing disulfide linkages. (i) Mild oxidation (I_2_/pyridine) reconnects α,ω-dithiol PS and PnBA blocks into high-molar-mass copolymers; (ii) reductive treatment (TCEP) cleaves the disulfide bonds. Reproduced from ref 278 with permission. Copyright 2025 Wiley-VCH.

This innovative approach illustrates a closed-loop recycling pathway, connecting for the first time two major families of traditionally incompatible vinyl polymers while maintaining excellent mechanical and thermal properties. It thus highlights the potential of lipoate-based systems for the design of recyclable and reconfigurable polystyrene–polyacrylate materials, offering precise control over composition, molar mass, and final material properties.

Besides linear polymers, a similar study has been performed on polyacrylate networks by Kopec et al.^217,218^ They first studied^217^ the regelation of degradation productions obtained by RAFT polymerization of *n*-butyl acrylate, 1 mol % of hexanediol diacrylate (HDDA) as cross-linker and DOT (3–5 mol %) and later degraded by cysteamine and DBU as the catalyst. The regelation was obtained after heating in air at 30 °C with pyridine to form disulfide linkages. All three samples successfully regelled into a solid disc. After going through to regelation, the PBA-DOT networks exhibit disulfide linkages in place of the initial thioesters; the second degradation was then carried out using EDDET, and all samples successfully underwent regelation following this second degradation (Figure 76). A sample comprising 4 mol % DOT and 1 mol % HDDA was synthesized using traditional FRP and then degraded via the cysteamine/DBU process previously outlined.

**Figure 76:**
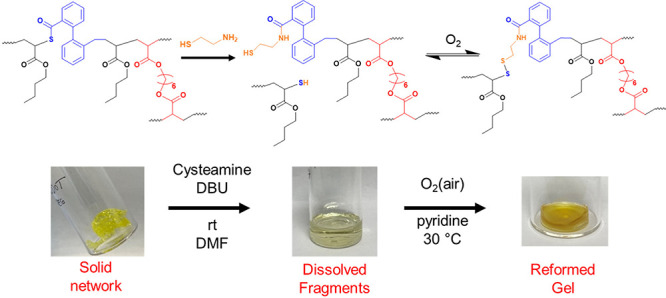
Degradation and regelation scheme for PBA-DOT networks prepared by RAFT polymerization. Reproduced from ref 217 with permission. Copyright 2023 Royal Society of Chemistry.

The fragments could not be regelled anymore whatever the conditions. The more homogeneous network topology in RAFT-produced gels is likely essential for achieving efficient reversibility.^217^

The same authors^218^ extended this work to lipoic acid and its ester ethyl lipoate as a additive to impart degradability to polyacrylate networks. In that case, loading of 15 mol % of ethyl lipoate and RAFT polymerization led to the best results. Neither the FRP-made gel could successfully establish a stable regenerated network. Unexpectedly, the reformation of the PBA-αLA and PBA-ELp gels synthesized using RAFT polymerization proved to be more challenging than the one obtained using DOT. The sole successful sample to completely transition to a solid network was the PBA-15% ELp-RAFT gel that led to the higher dispersity of the degraded oligomers. The authors supposed that “uniform distribution of the cleavable groups in the backbone, which is typically a sought-after feature that improves degradation by producing low dispersity fragments, might be detrimental if the network is designed to also reform after deconstruction”.^218^

While the introduction of labile, weak, or cleavable bonds into the backbone of commodity thermoplastics represents a well-established and conceptually promising strategy to enable chemical degradation, typically under accelerated conditions such as concentrated basic media, the actual end-of-life performance of these materials remains insufficiently characterized and requires substantial further development. Although preliminary results highlight the potential of incorporating such bonds into radically polymerized structures to confer biodegradability, comprehensive structure–biodegradability relationships across diverse polymer architectures are still lacking, and standardized biodegradation assessments under relevant laboratory and environmental (field) conditions are urgently needed, as emphasized in recent reviews. Concurrently, advancing a circular materials economy through enhanced recycling pathways appears to be the most logical and sustainable approach for collectable plastics, yet only limited proof-of-concept studies exist, underscoring the necessity for scaled-up feasibility investigations. In this context, water-soluble polymers employed in home care, personal care, detergency, and adhesive applications have emerged as particularly suitable early candidates for implementing cleavable comonomer chemistries, given the practical impossibility of collection and recycling combined with the scarcity of fully biodegradable alternatives in these sectors. Overall, bridging the current gaps between promising laboratory demonstrations and robust, application-specific degradation and recycling data will be essential to translate this approach into meaningful environmental impact reduction.

## Conclusion

Radical ring-opening polymerization (rROP) is now emerging as an essential strategy for the design of degradable vinyl polymers, addressing the environmental and societal challenges posed by the persistence of conventional polymers. This tutorial review has provided a structured overview of major advances in this growing field, from the historical foundations of rROP to recent developments aimed at concrete applications.

The analysis of different families of monomers, CKAs, SCMs, thionolactones, and lipoates highlighted their unique structures, synthesis methods, and distinct behavior in both homopolymerization and copolymerization. The reactivity of these monomers toward the most common vinyl monomers (e.g., styrene, acrylates, methacrylates, and less-activated monomers) has consistently demonstrated that solutions exist to effectively introduce heteroatoms and weak bonds into polymers that are otherwise considered nondegradable.

Finally, the wide range of applications presented, ranging from latexes and surface coatings to biomaterials, adhesives, and packaging, illustrates the growing maturity of rROP as an effective design tool. These contemporary and exciting results pave the way for a new generation of materials that combine performance, functionality, and a controlled end-of-life fate.

This review is intended as a practical guide to help orient the choice of monomers and polymerization strategies best suited for different contexts. Future developments will certainly need to focus further on overcoming current reactivity and compatibility limitations in order to democratize the use of degradable vinyl polymers in large-scale industrial applications.
